# Light-microscopy-based connectomic reconstruction of mammalian brain tissue

**DOI:** 10.1038/s41586-025-08985-1

**Published:** 2025-05-07

**Authors:** Mojtaba R. Tavakoli, Julia Lyudchik, Michał Januszewski, Vitali Vistunou, Nathalie Agudelo Dueñas, Jakob Vorlaufer, Christoph Sommer, Caroline Kreuzinger, Bárbara Oliveira, Alban Cenameri, Gaia Novarino, Viren Jain, Johann G. Danzl

**Affiliations:** 1 Institute of Science and Technology Austria, Klosterneuburg, Austria; 2 Google Research, Zürich, Switzerland; 3 Google Research, Mountain View, CA USA

**Keywords:** Super-resolution microscopy, Neural circuits, Cellular neuroscience, 3-D reconstruction, Confocal microscopy

## Abstract

The information-processing capability of the brain’s cellular network depends on the physical wiring pattern between neurons and their molecular and functional characteristics. Mapping neurons and resolving their individual synaptic connections can be achieved by volumetric imaging at nanoscale resolution^1,2^ with dense cellular labelling. Light microscopy is uniquely positioned to visualize specific molecules, but dense, synapse-level circuit reconstruction by light microscopy has been out of reach, owing to limitations in resolution, contrast and volumetric imaging capability. Here we describe light-microscopy-based connectomics (LICONN). We integrated specifically engineered hydrogel embedding and expansion with comprehensive deep-learning-based segmentation and analysis of connectivity, thereby directly incorporating molecular information into synapse-level reconstructions of brain tissue. LICONN will allow synapse-level phenotyping of brain tissue in biological experiments in a readily adoptable manner.

## Abstract (web summary)

A technique called LICONN (light-microscopy-based connectomics) allows mapping of brain tissue at synapse level and simultaneous measurement of molecular information, thus enabling quantification of cellular properties and multimodal analysis of brain tissue.

## Main

The brain is made up of an incredibly dense, complex and fine-grained arrangement of neurons with support cells, which together constitute a functional network that enables brain function. Imaging approaches are uniquely positioned to decode the spatial organization of the brain. Determining how neurons are connected and reconstructing the circuitry that underlies information processing—that is, determining connectomes—demands accurate tracing of cellular circuit components including axons and dendritic spines, resolving synaptic connections and assigning them to specific neurons.

Light microscopy holds considerable potential for unifying synapse-level circuit reconstruction with in-depth molecular characterization. However, its resolution is conventionally limited to several hundred nanometres (best case 200–300 nm laterally, and typically substantially worse (around 1,000 nm) along the optical (*z*) axis)—much too coarse to distinguish densely labelled cellular structures. Electron microscopy (EM), with its nanometre-scale resolution and comprehensive structural contrast, is at present the only technology that allows dense connectomic analysis (that is, comprehensive reconstruction of cellular circuit components^1–3^), and enormous strides have been made in using EM to map connectivity in organisms as diverse as worms^4^, flies^5–7^, mice^8^ and humans^9,10^. These advances were facilitated by technological progress in automated data collection and deep-learning analysis, which has made the challenge of densely annotating all cellular structures tractable^2,3^. EM sample preparation and readout are not directly compatible with visualizing specific molecules in circuit reconstruction, and require correlation with light microscopy to obtain molecular information^11–13^. EM reconstructions allow connectivity through chemical synapses to be inferred from structural features^14^. However, synapses cannot be further differentiated molecularly, and information related to signalling between cells, such as the distribution of receptor molecules that have key roles beyond classical synaptic transmission, remains lacking.

Super-resolution optical imaging offers resolution beyond the diffraction limit by increasing instrument resolution^15^ or by expanding samples to increase distances between features^16^, but it has been limited mostly to sparse subsets of cells or molecule distributions devoid of cellular context. For example, multicolour ‘Brainbow’ labelling with expansion and synaptic-marker detection allowed the connectivity of cellular subsets to be inferred^17^. To visualize living brain tissue comprehensively, fluorophores have been applied extracellularly, casting super-resolved cellular ‘shadows’ when imaged with stimulated emission depletion (STED) microscopy^18^ in super-resolution shadow imaging (SUSHI)^19^. Combining this with two-stage machine learning enabled reconstruction at three-dimensional (3D) nanoscale resolution with LIONESS (live information-optimized nanoscopy enabling saturated segmentation)^20^. Fixation-compatible extracellular labelling with CATS (comprehensive analysis of tissues across scales)^21^ visualized tissue architecture using STED or expansion microscopy (ExM). However, LIONESS and CATS have not provided the traceability and accuracy required for synapse-level circuit reconstruction. ExM^16^ increases effective resolution by embedding tissue in a swellable hydrogel, disrupting the tissue’s mechanical cohesiveness and expanding it. Expansion factor (exF) and corresponding resolution enhancement have been increased from around fourfold^16,22^ to around eight-to-tenfold^23–27^ in single-step approaches, and to around 16× and beyond with iterative application^28–30^ of two swellable hydrogels. Indiscriminate (‘pan-’) labelling for protein density using amine-reactive fluorophore derivatives, such as *N*-hydroxysuccinimidyl (NHS) esters, revealed cellular ultrastructure with single-step^25,31^ and iterative^30^ expansion and visualized the complexity of brain tissue^25,32^. However, it has not been possible to achieve light-microscopy imaging at the resolution and signal-to-noise ratio that are required for dense connectomic reconstruction.

**Fig. 1:**
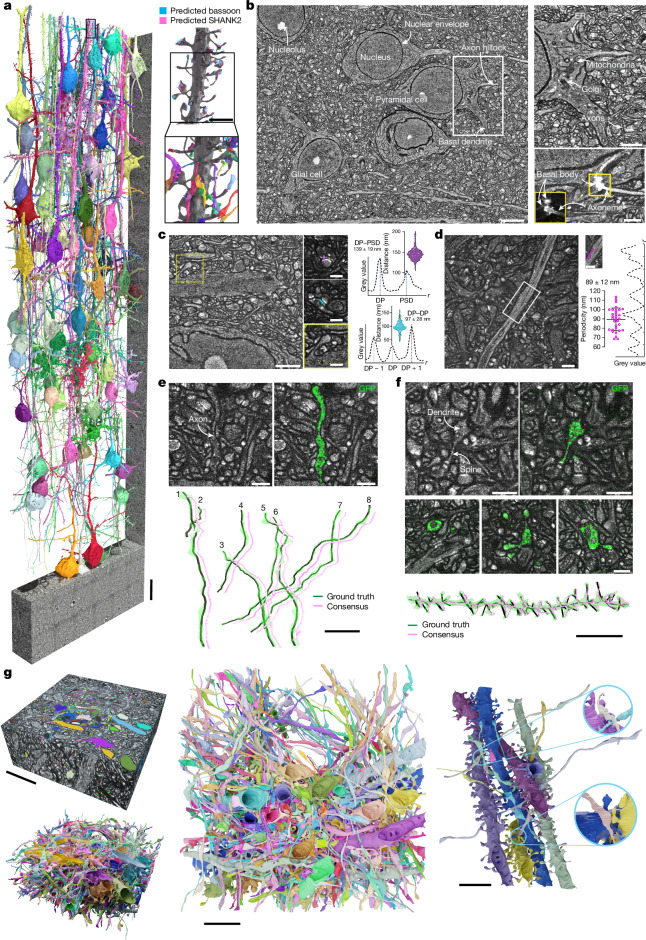
Dense connectomic reconstruction of mammalian brain tissue with light microscopy. **a**, LICONN volume of around 1 × 10^6^ μm^3^ (native tissue scale) of mouse primary somatosensory cortex (layers II/III–IV, 396 × 109 × 22 µm^3^ original tissue scale, 0.95 × 10^6^ µm^3^ before and 3.5 × 10^9^ µm^3^ after hydrogel expansion of approximately 16×). Seventy-nine example cells from dense reconstruction with FFN. Right, dendrite from pyramidal neuron (box at top of left panel), with deep-learning predictions of the synaptic molecules bassoon (cyan, pre-synapses) and SHANK2 (magenta, excitatory post-synapses) and synaptically connected axons (bottom). Scale bars, 15 μm (left); 2 μm (right). Length scales and scale bars refer to biological size before expansion throughout. **b**, Subregion (single plane) of **a**. Enlarged views: top, intracellular structure of pyramidal neuron; bottom, primary cilium. Spinning-disk confocal imaging data with contrast-limited adaptive histogram equalization (CLAHE) (for comparison of raw versus CLAHE, see Supplementary Fig. 12). Scale bars, 5 μm (left); 2 μm (top right); 1 μm (bottom right). **c**, Single plane (with CLAHE) of dendrite with spine (box) and cell nucleus with nuclear pores (bright densities, bottom). Small panels: top, excitatory synapse with presynaptic protein-rich punctate features and bar-like feature at post-synapse (without CLAHE). Line: direction of intensity profiles (top right), measuring distance between pre- and postsynaptic features (DP: dense projection; mean ± s.d.) with violin plot. *r*: coordinate along line profile. Middle, single bouton contacting two spines and DP–DP distance (mean ± s.d.). Bottom, highlighted spine. Scale bars, 2 μm (main image); 100 nm (right images). **d**, Periodic, protein-dense structure at circumference of neurite subset (without CLAHE). Example line profile and periodicity (mean ± s.d.). Scale bars, 1 μm. **e**, Manual axon tracing. Top left, single plane (magnified) of LICONN volume (around 19 × 19 × 19 µm^3^) from primary somatosensory cortex in a *Thy1-eGFP* mouse with cytosolic eGFP expression in an axon (arrow). Top right, overlay with immunolabelling for eGFP. Scale bars, 1 μm. Bottom, renderings (green) and skeletons (black) of eight eGFP-expressing axons (ground truth, based on eGFP and structural LICONN channels), and skeletons generated by two annotators blinded to eGFP signal (magenta, consensus, offset for clarity). For additional datasets, see Supplementary Figs. 13 and 14. Scale bar, 5 μm. **f**, Manual dendrite tracing. Top, single plane (magnified) from LICONN volume (around 19 × 19 × 19 µm^3^, hippocampus, CA1; *Thy1-eGFP* mouse) with eGFP-expressing dendrite (arrow) and overlay with eGFP (green). Middle, cross-sections of eGFP-expressing dendrite with spines. Scale bars, 1 μm. Bottom, dendrite skeleton (black) generated from eGFP and structural channels (ground truth), within 3D rendering (green). Additional skeleton (magenta) generated from structural channel by two annotators blinded to eGFP. For additional datasets, see Supplementary Fig. 15. Scale bar, 5 μm. **g**, Left, single-tile LICONN volume (hippocampus, CA1) with manual cellular annotations (colour, 658 structures) and 3D rendering. Middle, top view. Right, neurites and magnified synaptic connections (different camera position). Scale bars, 5 μm (left); 3 μm (middle and right). Source Data

Here we present a technology that can be used to densely reconstruct brain circuitry with light microscopy at synaptic resolution. We engineered a high-fidelity iterative hydrogel expansion scheme paired with protein-density staining and high-speed diffraction-limited readout that enables manual neuronal tracing and deep-learning-based cellular segmentation (Fig. 1a). We show traceability of the finest neuronal structures, including axons and dendritic spines; simultaneous molecular measurement; deep-learning prediction of molecule locations; and connectivity analysis at single-synapse resolution. We validate the technology using independent ground truth from sparse positive labelling and quantification of spine traceability. We furthermore provide comparisons of statistical data on neuronal connectivity with previous EM measurements, a method that has been used to cross-validate EM datasets^33^. This technology, which we term light-microscopy-based connectomics (LICONN), offers molecularly informed reconstruction of brain tissue with broad accessibility.

## Expansion of brain tissue for connectomics

Our strategy for dense light-microscopy-based connectomics achieves an increase in resolution through hydrogel expansion rather than optical super-resolution, exploiting the speed, optical sectioning and availability of standard, diffraction-limited, spinning-disk confocal microscopes. Hydrogel embedding^34^ and expansion homogenize the refractive index to that of water. This facilitates the acquisition of volumes extended both laterally and along the *z* axis, which is notoriously difficult with other super-resolution techniques because of aberrations, scattering and photobleaching. It also allows facile multicolour readout of specific molecules at a resolution essentially identical to that of the structural channel.

To achieve high-fidelity tissue preservation and neuronal traceability, we developed an iterative expansion technology based on independent, interpenetrating hydrogel networks including tailored chemical fixation, retention of cellular proteins and hydrogel chemistry that obviated hydrogel cleavage and signal handover steps. Available single-step or iterative approaches provided insufficient performance and straightforward modifications to increase exF resulted in unstable hydrogels (Supplementary Figs. 1–3).

We transcardially perfused mice with hydrogel monomer (acrylamide, AA)-containing fixative solution, equipping cellular molecules with vinyl residues, which subsequently co-polymerized with the hydrogel. Optimization of perfusion, with a lower monomer concentration (10% AA) than that used previously^22,32^, improved cellular preservation (Supplementary Fig. 4), probably reflecting osmotic effects. Chemical fixation does not preserve the precise shape of extracellular space^19,33^. However, LICONN maintains low-signal-intensity regions around cells, which is advantageous for tracing and segmentation, similar to extracellular-space-preserving protocols^35^ in EM connectomics. We collected and sliced brains, and exploited the broad reactivity of multi-functional epoxide compounds^36,37^—specifically, glycidyl methacrylate (GMA) and glycerol triglycidyl ether (TGE, bearing three epoxide rings)—to functionalize proteins more broadly with acrylate groups for hydrogel anchoring than common amine-reactive compounds, and to further fix and stabilize biomolecules, respectively. Alternatively, amine-reactive anchoring^38,39^ using *N*-acryloxysuccinimide (NAS)^27^ resulted in traceable datasets (Supplementary Figs. 2, 5, 6), but epoxide use improved cellular ultrastructure and emphasized synaptic features.

We polymerized an expandable acrylamide–sodium acrylate hydrogel, integrating functionalized cellular molecules into the hydrogel network, and disrupted mechanical cohesiveness using heat and chemical denaturation^22^ (Supplementary Fig. 7). After around fourfold expansion, we optionally applied immunolabelling to visualize specific proteins. A non-expandable stabilizing hydrogel prevented shrinkage during the application of a second swellable hydrogel intercalating with the first two hydrogels. To achieve structural preservation and homogeneous expansion, we optimized the composition of the hydrogels (Supplementary Figs. 8, 9), and found that chemically neutralizing unreacted groups after each polymerization step improved high-fidelity expansion by abolishing cross-links between hydrogels, ensuring their independence. Finally, protein-density (‘pan-protein’) staining with fluorophore NHS esters comprehensively visualized cellular structures, mapping (primary) amines that were abundant on proteins.

These triple-hydrogel–sample hybrids yielded an expansion of around 16-fold and were mechanically robust, facilitating handling and extended imaging (exF = 15.44 ± 1.68, mean ± s.d., *n* = 4 technical replicates across *n* = 3 mice; Supplementary Fig. 10; unless otherwise stated, we give length measures in original tissue size by scaling measured post-expansion lengths with this exF). Expansion-induced distortions were similar to those in previous work^28,29,32^ (Supplementary Fig. 10). Spinning-disc confocal imaging with a high-numerical-aperture (NA = 1.15) water-immersion objective lens in the green spectral range yielded an expected resolution of around 280 nm laterally and around 730 nm axially. With an exF of around 16, this translated into effective resolutions of around 20 nm and and around 50 nm, respectively, demanding an effective voxel size of about 10 × 10 × 25 nm^3^ for adequate sampling. Overall, this workflow was robust and provided the resolution, contrast and throughput required for connectomic tissue reconstruction.

We analysed a tissue volume of around 1 × 10^6^ µm^3^ (native tissue scale, 396 × 109 × 22 µm^3^, 0.95 × 10^6^ µm^3^ pre-expansion, effective voxel size 9.7 × 9.7 × 25.9 nm^3^, 3.5 × 10^9^ µm^3^ post-expansion; Fig. 1a), spanning layers II/III–IV of the primary somatosensory cortex (Supplementary Fig. 11). A total of 132 partially overlapping subvolumes were imaged (arranged on a 6 × 22 grid) and an automated algorithm^40^ (scalable optical flow-based image montaging and alignment; SOFIMA) was used for seamless volume fusion. With high parallelization in spinning-disc confocal microscopy (around 300 focal points in our system), the approximately 4.2 × 10^9^-µm^3^ post-expansion size (0.47 teravoxels, including tile overlap) was imaged within 6.5 h. This corresponded to an effective voxel rate of 17 × 10^6^ voxels per second (17 MHz), including overhead from sample stage movement and tile overlap. Individual neurons with their axons and dendrites were clearly delineated from each other in densely packed neuropil and showed rich subcellular structures (Fig. 1b–d and Supplementary Fig. 12), including mitochondria, Golgi apparatus and primary cilia. When we inspected dendrites and their spines—postsynaptic structures that are typical of excitatory synapses (Fig. 1c)—we found putative synaptic transmission sites highlighted by protein-rich, high-intensity features, akin to postsynaptic densities (PSDs) in EM data on chemically fixed specimens^14^. Similarly, presynaptic sites exhibited protein-dense nanoscale features, arranged in a lattice-like pattern spaced at 97 ± 28 nm, at a distance of 139 ± 19 nm from PSDs (192 synapses, 3 technical replicates across *n* = 2 mice) (Fig. 1c), again similar to features seen in EM. We also observed prominent ring-like periodic patterns at the circumference of a subset of neurites (Fig. 1d and Supplementary Fig. 12). The periodicity of 89 ± 12 nm (32 distance measurements, *n* = 2 mice) was highly suggestive of the actin and β-spectrin cytoskeletal lattice that organizes specific proteins^41^ below the plasma membrane. Periodicity was consistent with the value of 182 nm that was previously reported when labelling only one component^41^. Together, these results indicate that our expansion and imaging procedure reports the cellular constituents of brain tissue with high fidelity from the tissue scale to the nanoscale.

## Tracing neuronal structures in LICONN

Thin, tortuous axons in dense neuropil and dendritic spines with their thin necks are among the most challenging structures for connectomic tracing. To evaluate the reliability of manual tracing, we compared human consensus skeletons with sparse fluorescent labelling. Specifically, we obtained ‘ground-truth’ neurites from cytosolically expressed enhanced green fluorescent protein (eGFP, detected by immunolabelling) in a subset of neurons in *Thy1-eGFP* mice. We compared those with independently traced skeletons of the same objects, manually generated exclusively from the LICONN structural channel (Fig. 1e,f). After training (Methods), 2 tracers received 12 LICONN datasets with 37 axon stretches (880 µm cumulative length, *n* = 3 technical replicates across *n* = 2 mice, cortex), with a seed point in each eGFP-expressing axon. Tracers were blinded to the eGFP signal itself. They independently traced the indicated axons, compared results and found consensus at locations of disagreement (Fig. 1e). Of 37 axons analysed, the consensus skeletons followed a wrong path in one case, compared with eGFP ground truth (1.1 errors per mm; Fig. 1e and Supplementary Figs. 13, 14). In a similar analysis of eGFP-expressing dendrites in the hippocampus, the blinded tracers correctly identified 259 out of 289 spines (Fig. 1f and Supplementary Fig. 15; 90%, *n* = 3 technical replicates across *n* = 2 mice).

**Extended Data Fig. 1:**
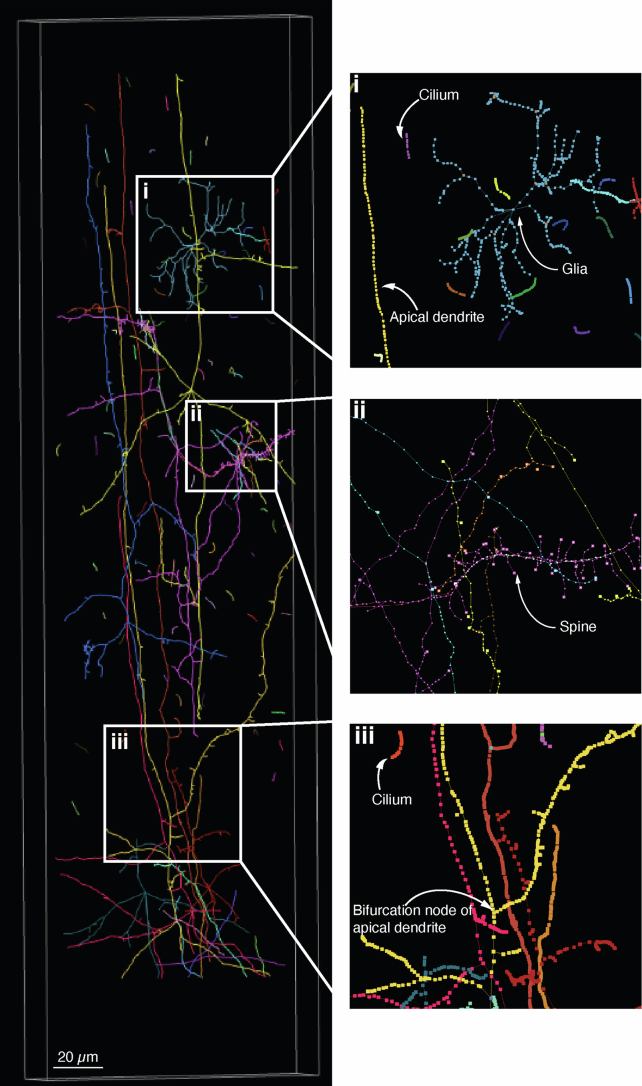
Tracing of neuronal structures in the cortex. Manually generated skeletons in the dataset in Fig. 1a, showing traceability of axons and dendrites across borders of fused imaging tiles. Magnified views indicate examples of glial cells, primary cilia used in further analysis (Fig. 5) and dendrites. Spines of a particular dendrite were only comprehensively traced in the right part of the dendrite indicated in the magnified panel ii.

Encouraged by the consistency between LICONN-derived skeletons and their eGFP ground truth, we returned to the 1 × 10^6^-µm^3^ cortical dataset in Fig. 1a. We validated the traceability of axons and dendrites across tile borders (Extended Data Fig. 1). We further sought to exclude the possibility of sizeable numbers of non-traceable spines, and used an exhaustive tracing analysis of local volumes. We sampled 38 subvolumes of 2 × 2 × 2 µm^3^ at random locations. An expert annotator marked all spine heads on the basis of morphology and PSDs; spine density was 1.0 ± 0.3 per µm^3^ (mean ± s.d.), consistent with previous cortical data^1^. The annotator then manually attached spine heads to parent dendrites. Of 306 spine heads, 285 (93.1%) were unambiguously traced to a dendrite. Overall, this confirmed the high traceability of LICONN data and excluded the presence of a large population of non-traceable ‘orphan’ spines.

To test whether LICONN enabled volumetric annotation, we manually reconstructed neuronal structures in a 19.3 × 19.3 × 8.1-µm^3^ volume (imaged at an effective voxel size of 9.7 × 9.7 × 13.0 nm^3^) from the hippocampal CA1 stratum oriens (Fig. 1g and Supplementary Video 1). We reconstructed 658 structures, revealing their complex shapes and interwoven arrangement. This showed that LICONN is suitable for detailed volumetric annotation. However, manual reconstruction scales poorly, and would be difficult to apply comprehensively for the volume in Fig. 1a.

## Automated segmentation with flood-filling networks

Having manually validated traceability and segmentability, we analysed larger volumes by adopting deep-learning segmentation algorithms from EM connectomics. Specifically, we trained flood-filling networks (FFNs)^42^, which have achieved state-of-the-art segmentation accuracy on diverse connectomic datasets.

**Fig. 2:**
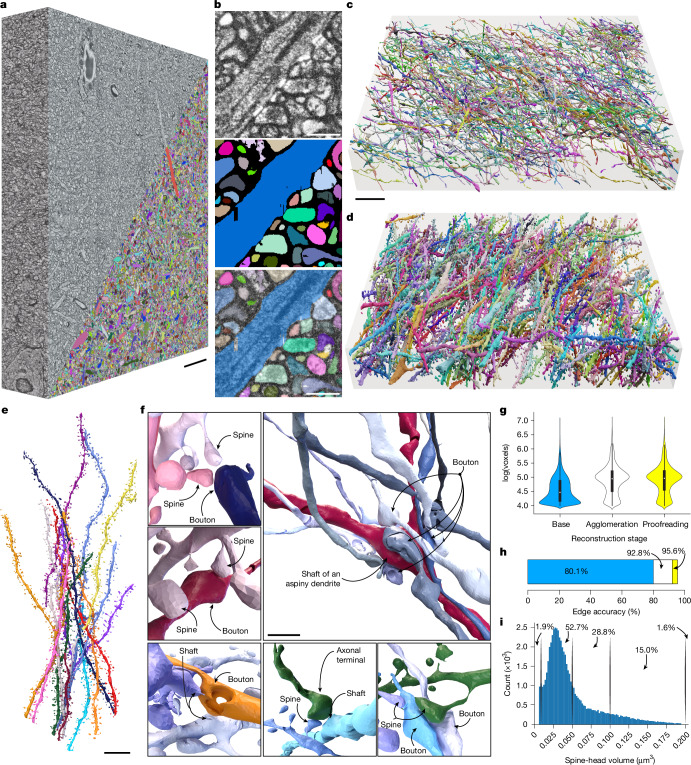
Deep-learning-based segmentation. **a**, Rendering of 85 × 69 × 14-µm^3^ LICONN volume (native tissue scale) from hippocampal neuropil in CA1, overlaid with dense FFN-based segmentation of neuronal structures in the bottom corner. Neuronal structures were comprehensively proofread in this volume by correction of split and merge errors (without any manual painting of voxels; see https://neuroglancer-demo.appspot.com/#!gs://liconn-public/ng_states/expid82.json for original data and proofread segmentation). Scale bar, 10 μm. **b**, Magnified view from a single plane with (top to bottom) raw structural data (CLAHE applied), dense segmentation after proofreading and overlay. Scale bars, 1 μm. **c**, Rendering of 5.8% of axons contained in the volume in **a** (see https://neuroglancer-demo.appspot.com/#!gs://liconn-public/ng_states/expid82_fig2_axons.json for browsable data). Scale bar, 10 μm. **d**, Rendering of 27.3% of dendrites and a small number of axons (see https://neuroglancer-demo.appspot.com/#!gs://liconn-public/ng_states/expid82_fig2_dends.json for browsable data). **e**, Rendering of example dendrites. Scale bar, 10 μm. **f**, Spatial arrangement of selected axonal and dendritic structures, highlighting various types of contact. Scale bar (top right), 2 μm. **g**, Segment size (number of voxels on a logarithmic scale) of neuronal structures for the base FFN segmentation (blue), after automated agglomeration (white) and after full manual proofreading of the automated agglomeration (yellow) for the dataset in **a** (*n* = 1 dataset). Vertical bar, lower and upper quartiles; dot, median; vertical lines, 1.5× interquartile range. **h**, Edge accuracy for the base segmentation (blue), after automated agglomeration (white) and after manual proofreading (yellow). **i**, Distribution of spine-head volumes in the same segmentation volume, analysed for 59,332 spines. Percentage numbers refer to the intervals indicated by the vertical lines (<0.01, 0.01–0.05, 0.05–0.1, 0.1–0.2, >0.2 µm^3^). Source Data

**Extended Data Fig. 2:**
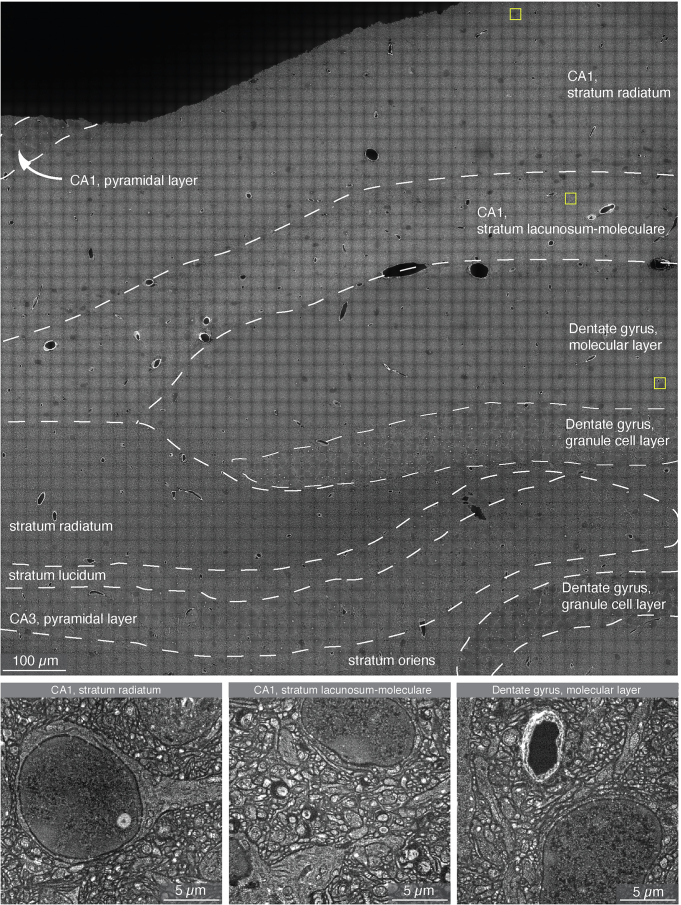
Hippocampal architecture analysed with LICONN. Hippocampal overview composed of 2,969 high-resolution tiles from spinning-disc confocal microscopy with delineation of subregions and layers. Yellow boxes indicate the regions magnified in the bottom panels.

We imaged a 109 × 74 × 22-µm^3^ region in the hippocampal CA1 (Fig. 2a and Extended Data Fig. 2) in a 4 × 6 tile arrangement with an effective voxel size of 9.7 × 9.7 × 13.0 nm^3^. Within this volume, we chose an 83,825-µm^3^ bounding box (85 × 69 × 14 µm^3^, 68.6 gigavoxels at native imaging resolution) and produced ground-truth annotations by iterative model predictions and manual proofreading (Methods).

An FFN was trained on the ground-truth annotations, applied to the whole bounding box (Fig. 2) and evaluated on 99 manually skeletonized neurites (69 axons, 1.8 mm cumulative path length, 30 dendrites with 1,041 dendritic spines; Supplementary Figs. 16, 17). The mean spine density along skeletonized dendrites (1.6 ± 0.3 per µm, nine dendrite stretches) was similar to that in previous EM data^43^. We optimized the FFN base segmentation (Methods) to minimize merge errors, as confirmed by comparison with the skeletons (0 mergers, 413 splits, 80.1% edge accuracy; see ref. ^42^ for metric details). We applied automated agglomeration^42^, which increased the edge accuracy to 92.8% (Fig. 2h), reducing splits by 92.5% (from 413 to 31), with some misattached spines but no mergers between axons or dendrites.

We then attempted to eliminate remaining errors in the automated reconstruction through comprehensive manual proofreading of the entire 83,825-µm^3^ volume (correcting object-level split and merge errors; Methods). We labelled objects in the proofread segmentation as axons, dendrites or glia using an automated classifier (Methods) and found 18,268 axons with a cumulative length of 342.3 mm, of which 5.8% (by length) are shown in Fig. 2c (segmentation: https://neuroglancer-demo.appspot.com/#!gs://liconn-public/ng_states/expid82_fig2_axons.json), and 1,643 dendrites with a cumulative length of 119.1 mm (Fig. 2d,e), of which 27.3% are shown in Fig. 2d (segmentation: https://neuroglancer-demo.appspot.com/#!gs://liconn-public/ng_states/expid82_fig2_dends.json), with 71,269 spines. Using the manually generated skeletons, we evaluated the contributions of base segmentation, automated agglomeration and manual proofreading to increasing segment size and edge accuracy (Fig. 2g,h). The proofread reconstruction yielded an edge accuracy of 95.6% with one morphological merger, 29 incorrectly attached spines (2.8% of spines), 14 uncorrected spine splits (1.4%) and zero splits involving axons or dendrite trunks. We did not process glial segments and blood vessels further, because the FFN models were trained exclusively on neuronal structures. Dense segmentation (browsable at https://neuroglancer-demo.appspot.com/#!gs://liconn-public/ng_states/expid82.json) thus revealed the complex 3D arrangements of neuronal structures in nanoscale detail (Fig. 2e,f and Supplementary Video 2).

Visual inspection suggested that signal-containing regions that were not covered by segments corresponded mainly to slight deviations in segment shape. Areas that were not captured in automated segmentation contained mostly intracellular regions or spines. We therefore quantified the traceability of spines in the volume, randomly sampling 40 subvolumes of 2 × 2 × 2 µm^3^. An annotator traced 281 of 301 spines (93.4%) to a parent dendrite, using only raw imaging data. The spine density was 0.9 ± 0.3 per µm^3^, similar to previous reports in CA1 (ref. ^43^). We next compared ground-truth skeletons with the FFN segmentation, in which the remaining error was dominated by spines that were not labelled by the FFN (83 of 1,041 spines in the skeletons, 8.0%). Nearly all spine necks in the automated segmentation were attached to a dendrite; occasionally the FFN neglected to segment voxels at spine necks, but agglomeration and proofreading were mostly still able to attach spine heads to the correct dendrite (1,000 of 1,041 spines on the manually generated skeletons). Measured spine-head volumes (Fig. 2i; 53% within 0.01 and 0.05 µm^3^) were consistent with EM data^44^.

Overall, LICONN enabled FFN-based segmentation with automated accuracy comparable to state-of-the-art EM results^7,42^ and manual correction of remaining errors using standard connectomic proofreading workflows.

## Molecularly annotated connectomics

We next sought to take advantage of the ability of light microscopy to visualize specific molecules, to directly verify cellular, subcellular and synaptic identities and place molecules in the context of the tissue’s 3D architecture and connectivity. Post-expansion immunolabelling (applied here after the first expansion) avoided extra tissue processing before expansion and promoted epitope accessibility^22,29^ in the expanded, molecularly decrowded tissue–hydrogel hybrid. This approach also renders the displacement of fluorophores from biological targets irrelevant by effectively ‘shrinking’ antibodies from their physical size of around 10 nm to less than 1 nm. Although certain epitopes are denatured during expansion, our limited screen identified a range of commercial antibodies that are compatible with LICONN. Iterative expansion with multicolour readout in standard spinning-disc confocal microscopes directly provided super-resolution measurements of structural and molecular channels, which is difficult when correlating light microscopy with EM connectomics (Supplementary Fig. 18).

**Fig. 3:**
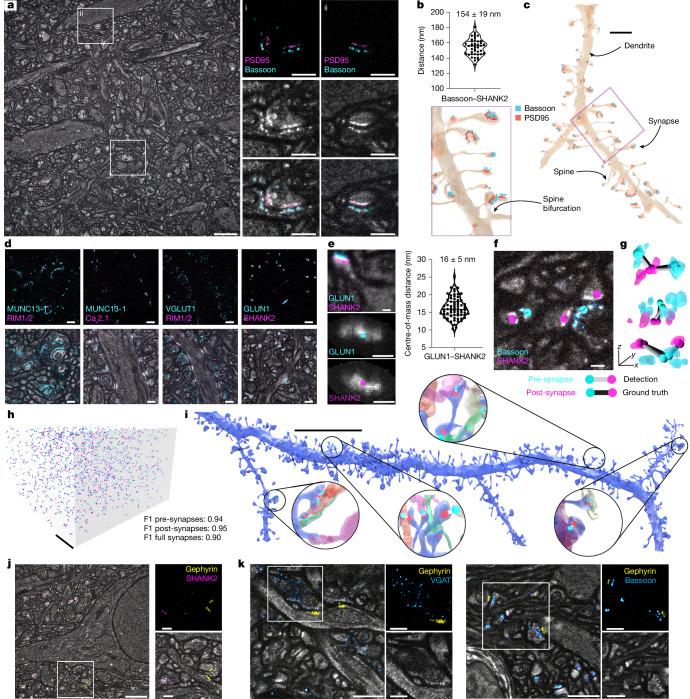
Molecular labelling and synapse detection. **a**, Immunolabelling for presynaptic bassoon (cyan) and postsynaptic PSD95 (magenta) in LICONN volume (somatosensory cortex). Right, magnified views of synapses (boxes in left panel) showing immunolabelling and structural channels separately and overlaid. Scale bars, 2 μm (left); 500 nm (right). **b**, Distance between bassoon and SHANK2 signals (mean ± s.d., violin plot including median and quartiles, 106 synapses). **c**, Three-dimensional renderings of bassoon and SHANK2 immunolabelling mapped onto a dendrite from FFN segmentation of the volume in **a**. Scale bar, 2 μm. **d**, Top, LICONN with immunolabelling for synaptic markers. Bottom, overlay with structural channel. Single planes from volumes in hippocampal CA3 (stratum lucidum, leftmost panel) and CA1. Scale bars, 500 nm. **e**, Left, dendritic spine with SHANK2 (magenta) and GLUN1 (cyan) immunolabelling, showing maximum intensity projections of respective immunolabellings (points: centres of mass). Scale bars, 100 nm. Right, mean ± s.d. and violin plot of centre-of-mass distances, including median and quartiles (184 synapses). **f**, Illustration of excitatory-synapse detection through bassoon (cyan) and SHANK2 (magenta) immunolabellings, converted to point annotations. Examples include 1:1 and 1:2 presynaptic to postsynaptic connections. Scale bar, 100 nm. **g**, Immunolabelling renderings with detected synapses (2:1, 1:1 and 1:2 presynaptic to postsynaptic connections). Cyan and magenta balls, pre- and post-synapses; grey and black bars, computationally detected connections and manually generated ground truth. **h**, Ground-truth (proofread) immunolabelling-based excitatory-synapse detections in a 913-µm^3^ volume (hippocampus, CA1, stratum radiatum). Scale bar, 2 μm. **i**, Three-dimensional rendering of dendrite (hippocampus, FFN segmentation) with excitatory synapses (bars), detected through bassoon (pre-synapses, cyan) and SHANK2 (post-synapses, red) immunolabelling. Magnified views include synaptically connected boutons. Scale bar, 10 μm. **j**, LICONN with immunolabelling for gephyrin (yellow, inhibitory post-synapses) and SHANK2 (magenta, excitatory post-synapses), with immunolabelling shown separately and overlaid for the boxed region. Inhibitory post-synapses show less pronounced structure than excitatory post-synapses. Scale bars, 2 μm (left); 500 nm (right). **k**, Left, LICONN with immunolabelling for VGAT (cyan, inhibitory pre-synapses) and gephyrin (yellow), with channels shown separately for the boxed region. Right, similar measurement with immunolabelling for bassoon (cyan, excitatory and inhibitory pre-synapses) and gephyrin (yellow). Scale bars, 1 μm (main); 500 nm (enlarged boxes). Source Data

**Extended Data Fig. 3:**
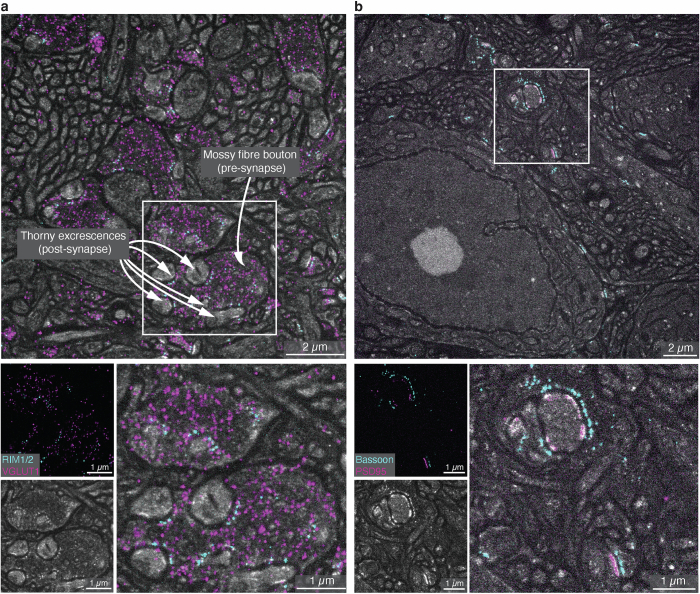
Synaptic labelling in mossy fibre boutons in the CA3 stratum lucidum. **a**, Mossy fibre boutons forming synapses with complex spines (thorny excrescences) at proximal dendrites of CA3 pyramidal neurons in hippocampus, interspersed with bundles of mossy fibres (non-myelinated axons of DG granule cells). Single plane of a LICONN imaging volume including the structural channel (grey) and immunolabelling for VGLUT1 (vesicular glutamate transporter 1, magenta), a synaptic vesicle marker in excitatory synapses, and the active-zone marker RIM1/2 (cyan). Bottom: Immunolabelling and structural channels displayed separately and as overlay for the two mossy fibre boutons and their postsynaptic partners in the box in the top panel. **b**, Similar measurement with immunolabelling for presynaptic bassoon and the postsynaptic scaffolding protein PSD95, present at excitatory synapses. Immunolabelling for RIM1/2 and VGLUT1 is representative of *n* = 2 technical replicates and bassoon/PSD95 of *n* = 3 replicates across *n* = 2 mice.

We first focused on synaptic proteins by immunolabelling bassoon, a presynaptic active-zone scaffolding protein, and PSD95, a postsynaptic scaffolding protein at excitatory synapses, and visualizing them in structural context in triple-colour measurements (Fig. 3a). At the high 3D resolution achieved here, bassoon labelling revealed lattice-like arrangements of nanoscale spots, recapitulating the protein-dense features in the structural channel (Fig. 1c). Both PSD95 and SHANK2, another postsynaptic scaffolding protein, were arranged in more compact, disc-like arrangements mirroring PSDs in the structural channel. Distance measurements between bassoon and SHANK2 yielded 154 ± 19 nm (Fig. 3b; mean ± s.d., 106 synapses, *n* = 2 mice), comparable to previous measurements from cultured neurons^45^. Applying the FFN-segmentation model from Fig. 2a–f located the synaptic molecular machinery to the respective neuronal structures, such as spiny dendrites (Fig. 3c). We labelled additional synaptic proteins in LICONN’s super-resolved structural framework (Fig. 3d and Extended Data Fig. 3). As expected, the active-zone markers MUNC13-1 and RIM1 and RIM2 (RIM1/2) spatially overlapped at pre-synapses. We also visualized P/Q-type Ca^2+^-channels (Ca_V_2.1) at pre-synapses. Labelling for the vesicular glutamate transporter VGLUT1 highlighted synaptic vesicles, and co-labelling with RIM1/2 concomitantly demarcated active zones. The NMDA glutamate receptor GLUN1 overlapped with SHANK2 at post-synapses, with the centres of mass of their 3D distributions spaced 16 ± 5 nm apart (185 synapses, *n* = 1 mouse; Fig. 3e).

## Molecularly inferring synaptic connectivity

Proximity between neurites is a weak predictor of synaptic connectivity^1^. We therefore used molecular information as ground truth for connectivity and developed an automated synapse identification pipeline based on immunolabelling (Fig. 3f–h and Supplementary Fig. 19). We first computationally annotated pre-synapses and excitatory post-synapses as defined by the presence of bassoon (excitatory and inhibitory pre-synapses) and SHANK2 (excitatory post-synapses), respectively (Fig. 3f); sampling intensity in the structural channel facilitated distinguishing synaptic signal from unavoidable immunolabelling background (Methods). We then automatically matched corresponding pre- and post-synapses to full synapses, including both one-to-one and one-to-many connections (Fig. 3g,h). Unpaired pre-synapses corresponded to inhibitory synapses, missed SHANK2 detections (for example, owing to low copy number or partial epitope degradation resulting in sub-threshold signal) or infrequent synapses lacking SHANK2 (but identifiable as excitatory because of a prominent PSD). We therefore revisited unpaired pre-synapses and classified neighbouring post-synapses as excitatory if a prominent PSD was present in the structural channel. When comparing purely automated synapse detections with manually validated synapse locations in a test dataset (1,059 excitatory synapses), we obtained 95% accuracy for the detection of pre- and post-synapses, and 90% for fully assembled synapses (Supplementary Fig. 19) (913 µm^3^ volume, F1: range 0–1, combining precision and recall; pre-synapses: F1 = 0.94; post-synapses: F1 = 0.95; full synapses: F1 = 0.90). We found detection to be robust against variation in imaging parameters and applicable in both hippocampus and cortex (Supplementary Fig. 19). Finally, by integrating FFN-based neuron segmentation with automated synapse detection, we inferred excitatory axonal inputs onto a specific dendrite (Fig. 3i). LICONN thus allowed us to map molecularly defined synaptic connectivity onto automated morphological reconstructions.

## Identification of excitatory and inhibitory synapses

We then used immunolabels to molecularly identify and distinguish excitatory and inhibitory synapses. To identify inhibitory synapses, we labelled gephyrin, a postsynaptic scaffolding protein. Gephyrin-positive, inhibitory post-synapses were less conspicuous structurally than their excitatory, SHANK2-positive counterparts, and these molecules were mutually exclusive at individual synapses (Fig. 3j). As a consistency check, we also co-labelled vesicular GABA transporter (VGAT) and gephyrin, identifying pre- and postsynaptic compartments in the same inhibitory synapses. As expected, presynaptic bassoon and postsynaptic gephyrin were closely juxtaposed (Fig. 3k). Molecular labelling thus distinguished excitatory and inhibitory synapses in LICONN volumes.

## Connectivity analysis

**Fig. 4:**
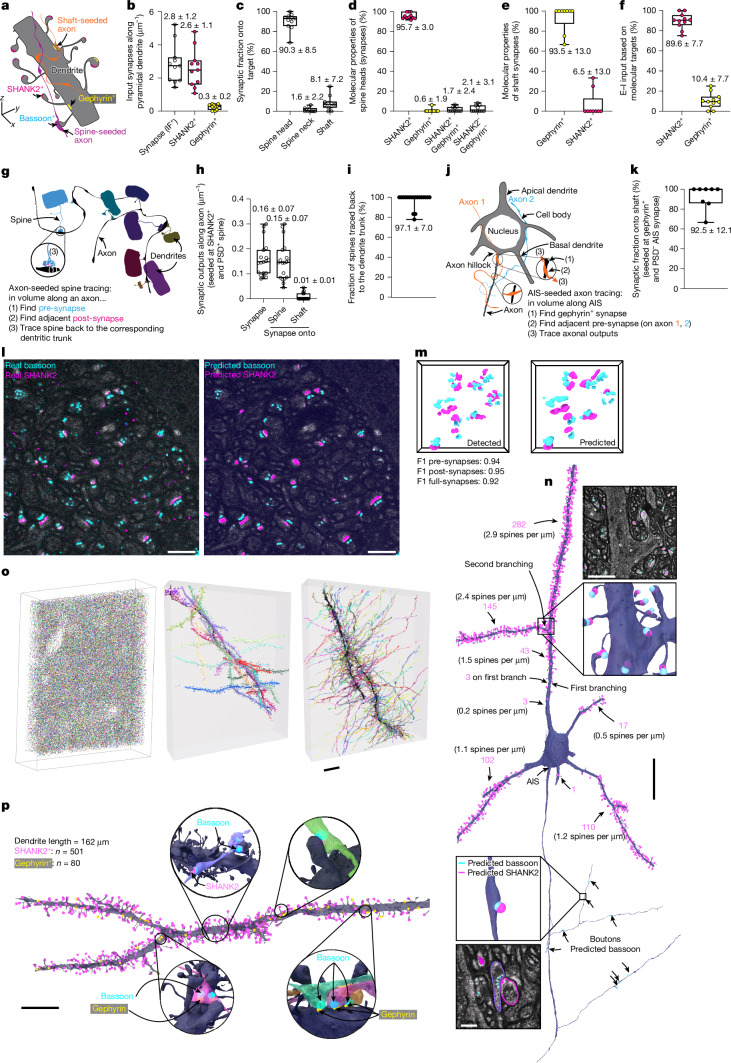
Connectivity analysis and deep-learning-based synapse detection. **a**, Spiny dendrite with SHANK2-expressing excitatory (magenta) and gephyrin-expressing inhibitory post-synapses (yellow), connected axons (presynaptic bassoon: cyan) and seed locations. **b**, Input density onto spiny dendrites (primary somatosensory cortex), defined by immunolabelling: excitatory (SHANK2^+^); inhibitory (gephyrin^+^); IF^+^: total immunofluorescence. Numerical values: mean ± s.d. throughout; box plots: median; lower and upper quartiles; whiskers: minimum and maximum (throughout). Data points: individual dendrites (11; 123 µm total, 351 IF^+^ synapses, **b**–**f**). **c**, Synapse (SHANK2^+^ or gephyrin^+^) target locations. **d**, Molecular properties of spine heads, with synapses identified by immunolabelling or PSDs. **e**, Molecular properties of shaft synapses. **f**, Excitatory (E) versus inhibitory (I) synapses onto dendrites. **g**, Spine seeding by excitatory axon outputs. **h**, Output density of spine-seeded axons (19 axons (data points) with two or more outputs, total length 990 µm). **i**, Spine fraction traced to parent dendrite for spines contacted by the same 19 axons (128 spines, mean ± s.d. over individual axon). Data points: axons. **j**, Inhibitory axons seeded at an AIS (gephyrin^+^, no PSD). **k**, Output fraction onto shafts of AIS-seeded axons (eight axons with three or more outputs). **l**, Deep-learning prediction of bassoon and SHANK2 location from structural LICONN channel. Single plane from LICONN volume (CA1, stratum radiatum, not included in training), comparing prediction with immunolabelling. Scale bars, 1 μm. **m,** Corresponding volumetric renderings. **n**, Excitatory input and output for a pyramidal neuron from the dataset in Fig. 1a. Synapse detections through bassoon and SHANK2 prediction mapped onto FFN segmentation. Magenta numbers: detected synapses between indicated branch points. Magnified views: structural channel, molecule predictions and cellular segments (partial proofreading, eliminating false-positive detections without adding missed detections). Scale bars, 2 μm (top); 20 μm (middle); 500 nm (bottom). **o**, Connectivity prediction in hippocampal dataset from Fig. 2a. Left, rendered synapse predictions. Middle, axon (black) with connected dendrites. Right, dendrite (black) with connected axons. Scale bar, 10 μm. **p**, Integration of structural, immunolabelling and deep-learning analysis. Dendrite (primary somatosensory cortex) with immunolabelling-based detection of excitatory (501) and inhibitory (80) post-synapses. Insets: presynaptic partners identified by deep-learning bassoon prediction. Scale bar, 10 μm. Source Data

Next, we related molecular properties to neurite tracings for characterizing fundamental parameters of synaptic wiring, including inhibition and excitation. We imaged two LICONN volumes in primary somatosensory cortex (143 × 20 × 24 µm^3^ and 179 × 20× 24 µm^3^, one mouse), with immunolabelling for excitatory (SHANK2) and inhibitory (gephyrin) post-synapses (Fig. 4 and Supplementary Figs. 20, 21). We manually analysed 11 spiny dendrites (Fig. 4a; total length 123 µm, 322 spines) and found 2.8 ± 1.2 (mean ± s.d. throughout) synaptic inputs per µm length (Fig. 4b), with a higher density of SHANK2-positive (2.6 ± 1.1 per µm) than gephyrin-positive (0.3 ± 0.2 per µm) inputs. Synapses were found mostly on spine heads (90.3%), whereas 1.6% and 8.1% located to spine necks and shafts, respectively (Fig. 4c). As expected, the vast majority of spine heads were positive only for SHANK2 (95.7 ± 3.0%); on rare occasions, they had only a gephyrin-positive connection (0.6%) or a gephyrin-positive connection in addition to the usual excitatory input (1.7%). A similarly small number of spine heads (2.1%) had neither SHANK2- nor gephyrin-positive connections, but had a PSD in the structural channel, with SHANK2 either not expressed or not labelled (Fig. 4d). Shaft synapses were overwhelmingly inhibitory (gephyrin-positive, 93.5%), whereas 6.5% were positive for SHANK2. As expected, these molecules were mutually exclusive at any given synapse (Fig. 4e). Immunolabelling directly yielded the balance of excitatory versus inhibitory inputs to dendrites (90% versus 10%, respectively; Fig. 4f). The results were consistent when the analysis was repeated using structural information—that is, the locations of synapses in axon outputs (shaft, spine head or neck)—to classify axons into excitatory or inhibitory, as is commonly done in EM-based connectomics (Supplementary Fig. 21).

We next classified axons as excitatory according to structural and molecular criteria: we selected a spine and identified an axon synaptically connected to it (‘spine-seeded’), by requiring (i) a PSD in the structural channel and (ii) SHANK2 at the synaptic seed site. When we manually traced 19 such axons (total length 990 µm), they exhibited 0.16 ± 0.07 synaptic outputs per µm length (Fig. 4g,h), with 0.15 ± 0.07 per µm onto spines and 0.01 ± 0.01 per µm onto shafts, recapitulating the known preference of excitatory outputs for spines^10^. We used these axons as seeds for a further independent test of spine traceability (‘axon-seeded’ spine tracing), again allowing us to trace the vast majority of spine necks (96%, 123 out of 128) to the parent dendrite (Fig. 4i).

**Fig. 5:**
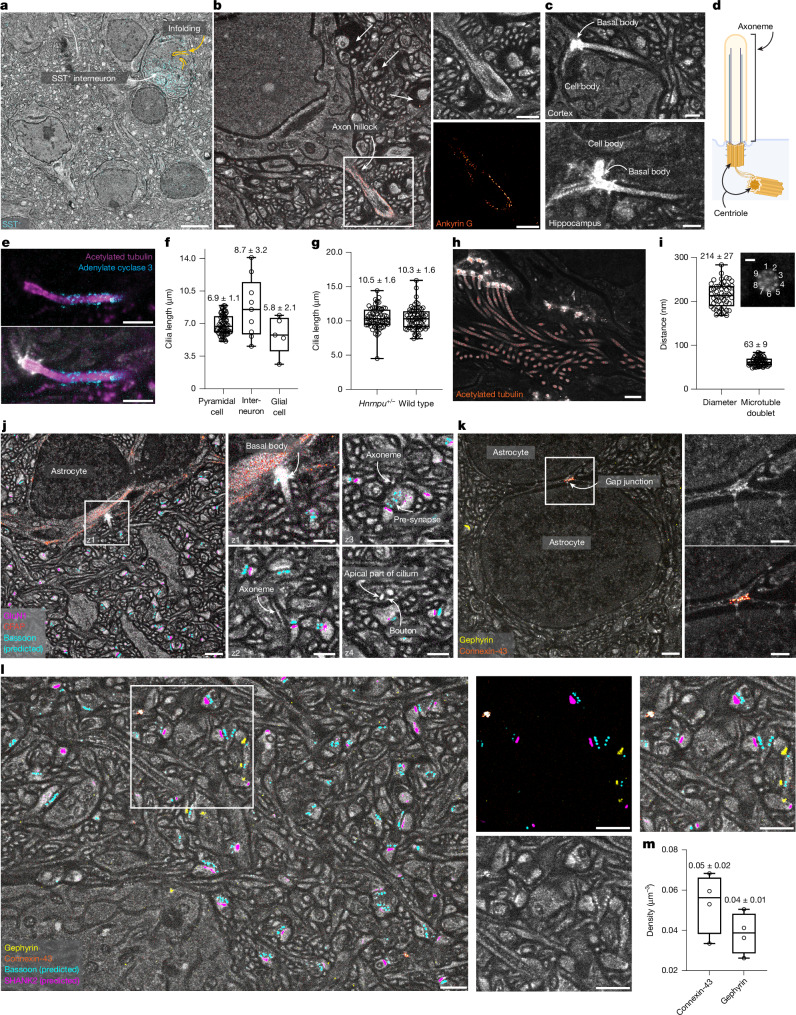
Applications of LICONN beyond synaptic connectivity. **a**, Molecular identification of interneurons through somatostatin immunolabelling (SST^+^, cyan). Overview plane of LICONN volume (cortex), with maximum intensity projection of immunolabelling. Nuclear infoldings: orange. Scale bar, 5 μm. **b**, Ankyrin G immunolabelling (red) (cortex), with periodic protein-density modulation in structural channel, highlighting the AIS. Magnified images: channels shown separately. Low-protein-density voids (straight arrows) around axons indicate myelination (Extended Data Fig. 5). Scale bars, 1 μm. **c**,**d**, Primary cilia in LICONN (cortex, hippocampus; **c**) with intensely labelled centrioles (schematic; **d**) in the basal body. Scale bars, 500 nm. **e**, Immunolabelling for acetylated tubulin (magenta) and adenylate cyclase 3 (cyan) at a primary cilium (hippocampus, CA1) and overlay with the structural LICONN channel, with the membrane-bound adenylate cyclase signal ensheathing axoneme. Scale bars, 500 nm. **f**, Length of primary cilia (mean ± s.d. throughout) according to cell type in dataset from Fig. 1a (78 cells; box plots: median; lower and upper quartiles; whiskers: minimum and maximum (throughout); points: individual cells). **g**, Length of primary cilia in wild-type and *Hnrnpu*^*+/−*^ mice (hippocampus, CA1, pyramidal layer; Supplementary Fig. 25). **h**, LICONN volume (border of corpus callosum and alveus; Extended Data Fig. 9) with immunolabelling for acetylated tubulin (red), revealing multi-ciliated cells. Scale bar, 1 μm. **i**, Cross-section of cilium with ninefold symmetry in protein density, probably reflecting microtubule doublets, with ring diameter and doublet distance. Scale bar, 100 nm. **j**, GFAP (red, astrocytes) and glutamate receptor GLUN1 (magenta) immunolabelling with deep-learning bassoon prediction (cyan) in LICONN volume (hippocampus). Magnified images: astrocytic primary cilium at different *z*-planes, apposed to synaptic boutons. Scale bars, 1 μm (main image); 500 nm (right images). **k**, Immunolabelling for the astrocytic gap-junction protein connexin-43 (orange) and the inhibitory-synapse marker gephyrin (yellow) in LICONN (cortex). Magnified images, gap junction between astrocytes. Scale bars, 1 μm (main image); 500 nm (right images). **l**, ‘Virtual’ five-colour measurement (cortex). LICONN with connexin-43 (orange) and (gephyrin, yellow) immunolabelling, deep-learning prediction of pre-synapses (bassoon, cyan) and excitatory post-synapses (SHANK2, magenta). Channels shown separately for boxed region. Scale bars, 1 μm. **m**, Density of gephyrin-positive inhibitory synapses and connexin-43-positive gap junctions (four volumes; cortex, hippocampal CA1). Source Data

For inhibitory axons (defined by (i) seeding from a synapse at a dendritic shaft; (ii) lack of PSD; and (iii) presence of gephyrin), we found an inverse target preference (Supplementary Fig. 21i,j). Finally, we selected three cells in which the entire axon initial segment (AIS) was contained in the imaging volumes (Fig. 4j). AISs were characterized by a pronounced periodic labelling pattern in the structural channel, akin to the actin- and spectrin-induced lattice in neurites (Figs. 1d, 5a), extending over 43.8 ± 1.3 µm. We detected 0.25 ± 0.15 gephyrin-positive inputs per µm AIS. We selected axons that provided inhibitory input to AISs (gephyrin-positive, no PSD) and traced their outputs. The 8 axon stretches analysed with 3 or more outputs formed 44 synapses at dendrites (synapses onto soma or AIS not analysed), with a strong preference for shafts (92.5%; Fig. 4k).

LICONN thus provided a natural means to integrate structural and molecular information to derive fundamental neuronal network parameters related to excitatory and inhibitory connectivity.

## Deep-learning-based prediction of synapse locations

To overcome limitations in microscopy hardware and imaging time from adding colour channels for further molecular targets, we used the correspondence between molecular and structural features to predict, rather than measure, the locations of synaptic molecules. We restricted prediction to excitatory synapses, which have more pronounced structural features than inhibitory synapses. Using deep learning, we predicted both bassoon at presynaptic sites and SHANK2 at excitatory post-synapses (Fig. 4l). Instead of human annotation, measurements of bassoon and SHANK2 (16,699 µm^3^, around 16,250 excitatory synapses), paired with the structural LICONN channel, were used to train neural networks^21,46^ that predicted the locations of molecules from the structural channel. When evaluated on datasets not included in training, predicted signals were highly consistent with ground-truth immunolabelling data (Fig. 4l,m). We converted molecule predictions to presynaptic and postsynaptic annotations and implemented post-processing (similar to that used in immunolabelling-based detection; Methods) to increase accuracy. Comparison of deep-learning-based synapse prediction with human ground-truth annotations yielded F1 scores higher than 0.9 for detecting synapses (hippocampus, CA1, stratum radiatum; pre-synapses: F1 = 0.94; post-synapses: F1 = 0.95; full synapses: F1 = 0.92, using 14,207 µm^3^ (around 13,650 synapses) for training and 180 µm^3^ for testing). We obtained a similar accuracy on test datasets with varied imaging parameters (*z*-step size) or brain region (cortex) (Supplementary Fig. 19). These results show that the locations of excitatory synapses can be predicted with high fidelity from structural LICONN data.

We then applied deep-learning-based synapse prediction to map the synaptic input field of an identified neuron in a dataset devoid of immunolabelling. We chose a pyramidal neuron from the FFN-based segmentation of the cortical dataset in Fig. 1a and related both pre-synapse (bassoon) and excitatory post-synapse (SHANK2) predictions to the cellular segmentation (Fig. 4n and Supplementary Video 3). The imaging volume contained 821 µm of this cell’s neurites. On dendrites (454 µm), we detected 705 excitatory synaptic inputs (1.6 per µm, 475 on apical and 230 on basal dendrites). The volume also contained 367 µm of the neuron’s axonal output, with eight predicted pre-synapses. We next applied the prediction of bassoon and SHANK2 to map excitatory synaptic connectivity onto the densely proofread neuronal segmentation in the hippocampus from Fig. 2a–f. We detected 79,291 pre-synapses (0.95 per µm^3^) and 71,976 excitatory post-synapses (0.86 per µm^3^) and mapped them onto individual neurite segments (Fig. 4o). Visualizing one axon with its synaptic partners shows rich connectivity, with 31 synapses onto 28 different dendrites. Similarly, focusing on a single spiny dendrite, 284 axons established 303 synaptic contacts with it (267 single connections, 16 axons with 2 synapses and one with 4 synapses; Fig. 4o).

**Extended Data Fig. 4:**
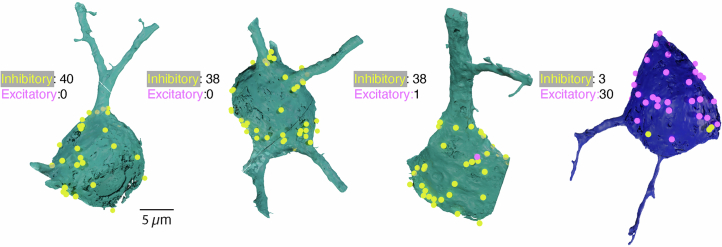
Excitatory and inhibitory synaptic inputs mapped onto reconstructed cell somata. Reconstruction of four example cell somata from LICONN datasets in somatosensory cortex with immunolabelling for SHANK2 and gephyrin. Excitatory (magenta) and inhibitory (yellow) synaptic inputs are mapped onto cell body reconstructions. Synapses were classified as excitatory if (1) a typical presynaptic structure, (2) a PSD and (3) SHANK2 were present. Synapses were classified as inhibitory if (1) a presynaptic structure was present and (2) a PSD was missing, with or without gephyrin. For each cell body, the number of excitatory and inhibitory connections are indicated, showing a much higher number of inhibitory inputs. The one cell on the right displayed a reversed pattern with 30 SHANK2-positive synapses. We analysed 13 cell bodies (contained ≥50% within the imaging volume) across 8 datasets and counted 48.7 ± 19.8 (mean ± s.d.) inhibitory synapses per soma (excluding the one cell with reversed input preference displayed on the right). Excitatory synapses were sparse, with most somata receiving 0 and one soma receiving 1 excitatory input.

We further created a ‘virtual’ four-colour connectomic volume combining the LICONN structural channel, deep-learning bassoon prediction (for detecting pre-synapses) and super-resolved immunolabelling of gephyrin and SHANK2 (for molecular synapse differentiation), again in the somatosensory cortex (subset of dataset in Fig. 4a–k). We found 501 excitatory and 80 inhibitory synapses on a dendrite reconstructed over a 162-µm length (Fig. 4p). By contrast, on cell somata, inhibitory connections were dominant (Extended Data Fig. 4). We further verified the robustness of deep-learning synapse prediction by replacing measured SHANK2 locations with predicted SHANK2 on the same dendrites and axons as in Fig. 4a–f. In this virtual four-colour volume (structure, measured gephyrin, predicted bassoon and predicted SHANK2), we obtained similar connectivity (Supplementary Fig. 22) to that found with SHANK2 immunolabelling, and quantifications of the balance of excitation and inhibition were in line with previous EM connectomics data^8,10,47^.

## Identification of cell types and subcellular structures

We now sought to use both structural and molecular information in LICONN to characterize cell types and subcellular specializations. Similar to EM connectomics, analysing cell shape enabled an expert to classify cells in the cortical dataset in Fig. 1a (78 cells analysed) as pyramidal neurons (64 cells, 82.1%), interneurons (9 cells, 11.5%), and glial cells (5 cells, 6.4%). The relative proportions were comparable to those in EM data from the mouse visual cortex^48^. Adding specific immunolabelling molecularly identified cell types, including inhibitory interneurons expressing somatostatin (Fig. 5a and Supplementary Fig. 23) or the voltage-gated potassium channel K_V_3.1b (Supplementary Fig. 24).

**Extended Data Fig. 5:**
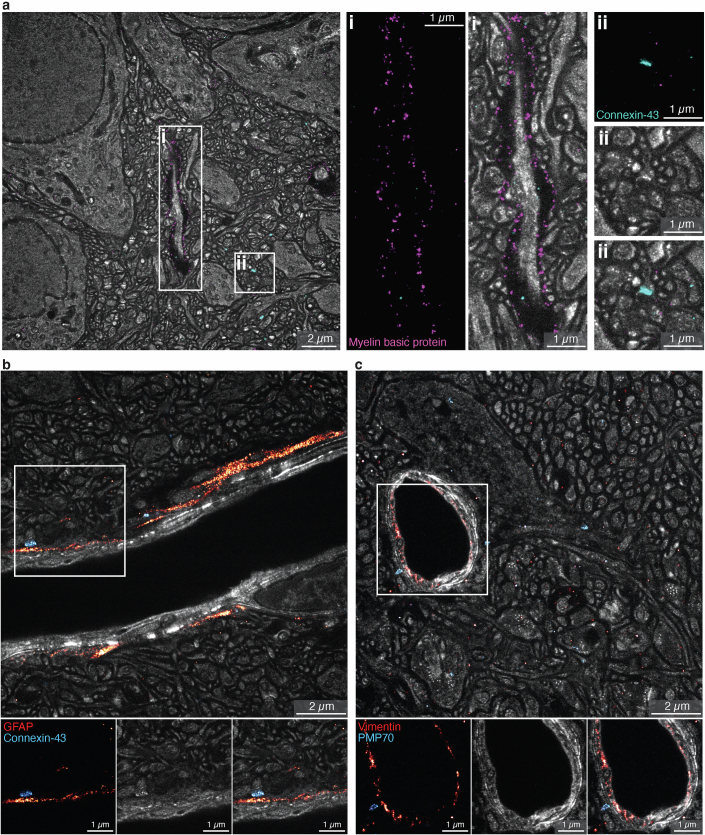
Molecular identification of cellular and subcellular structures. **a**, Single plane of a LICONN imaging volume in the hippocampal CA1 stratum radiatum, including the structural channel (grey) and immunolabelling for myelin basic protein (magenta) and the astrocytic gap-junction protein connexin-43 (cyan). Magnified views highlight structures expressing the respective molecular markers: (i) Immunolabelling for myelin basic protein identifies myelin sheaths, where the regions largely devoid of signal in the structural LICONN channel correspond to lipid-rich myelination. (ii) Connexin-43 indicates gap junctions. **b**, Single plane from LICONN imaging volume in hippocampus, showing the structural channel (grey) and immunolabelling for GFAP (orange) and connexin-43 (cyan). GFAP is an intermediate filament protein, commonly used as astrocyte marker. Note the GFAP signal in the immediate vicinity of the capillary traversing the image, and the cell nucleus on the right in the periphery of the blood vessel. Panels in the bottom show the immunolabelling and structural channels separately, as indicated by the box. **c**, Single plane from a LICONN imaging volume in the CA3 stratum lucidum, with additional immunolabelling for vimentin (orange, here highlighting the endothelial lining of a blood capillary) and the peroxisomal marker PMP70 (70 kDa peroxisomal membrane protein). Note that peroxisomes do not produce pronounced contrast in the structural (protein density) channel but can be identified by specific labelling. The displayed combinations of immunolabellings for MBP/Cnx-43 were technically replicated *n* = 1 time, RIM1/2 and VGLUT1 *n* = 2 times, GFAP/Cnx-43 *n* = 2 times, vimentin/PMP70 *n* = 1 time.

We then used molecular labelling to clarify the identity of subcellular structures and characterize their structural appearance. Labelling for ankyrin G molecularly highlighted AISs. We discovered that ankyrin G showed a lattice-like pattern at AISs, mirrored by a similar pattern in the protein-density map (Fig. 5b). This differentiated AISs from dendrites, which were mostly devoid of protein-density stripes near the soma. Similarly, labelling for myelin basic protein (Extended Data Fig. 5) clarified the identity of halo regions of low protein density around certain axons (Fig. 5b), unambiguously identifying them as myelinated axons.

Primary cilia (Figs. 1b, 5c–g) are key signalling hubs on many neuronal and non-neuronal cells, and their connectivity and function in the brain have been investigated^49,50^. We corroborated their identity by immunolabelling for acetylated tubulin, a common post-translational modification of stable microtubules, and adenylate cyclase 3 (Fig. 5e). We quantified cilia length according to cell type (Fig. 5f) from manually generated skeletons in 78 cells in the cortical dataset in Fig. 1a (mouse aged 2 months, 64 pyramidal neurons: 6.9 ± 1.1 µm; 9 interneurons: 8.7 ± 3.2 µm; 5 glial cells: 5.8 ± 2.1 µm). Ciliary function is essential for many aspects of brain development. In humans, mutations in the heterogeneous nuclear ribonucleoprotein U (*HNRNPU*) gene cause early-onset epilepsy, autistic features and intellectual disability. In the developing mouse brain, HNRNPU has been localized on primary cilia^51^. In addition, changes in the expression of genes associated with cilia organization have been observed in a human model system of induced-pluripotent-stem-cell-derived neurons from patients with *HNRNPU* mutations^52^. We hypothesized that mutations in *Hnrnpu* affect primary cilia length, and tested this in a haploinsufficient (*Hnrnpu*^*+/−*^) mouse model. We skeletonized primary cilia of pyramidal neurons in the hippocampal CA1 (Supplementary Fig. 25) in both *Hnrnpu*^*+/−*^ (80 cells, 7 technical replicates across *n* = 3 mice) and wild-type (78 cells, 7 technical replicates across *n* = 3 mice) mice (Fig. 5g), and concluded that, at least for hippocampal neurons, *Hnrnpu* mutations do not result in obvious defects in primary cilia (length (wild type): 10.3 ± 1.6 µm, length (mutant): 10.5 ± 1.6 µm, no statistically significant difference (*t*-test, *P* = 0.64)).

**Extended Data Fig. 6:**
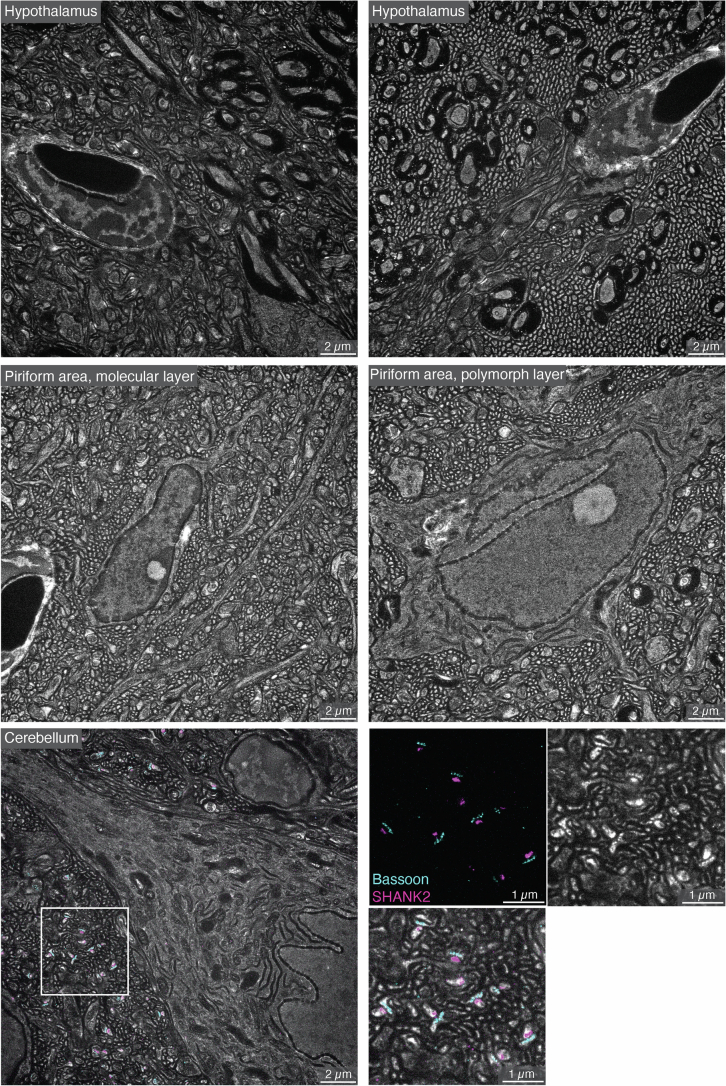
LICONN analysis in diverse brain regions. Single example planes from LICONN imaging volumes in hypothalamus, piriform area and cerebellum. In the cerebellum, additional immunolabelling for bassoon and SHANK2 was applied.

**Extended Data Fig. 7:**
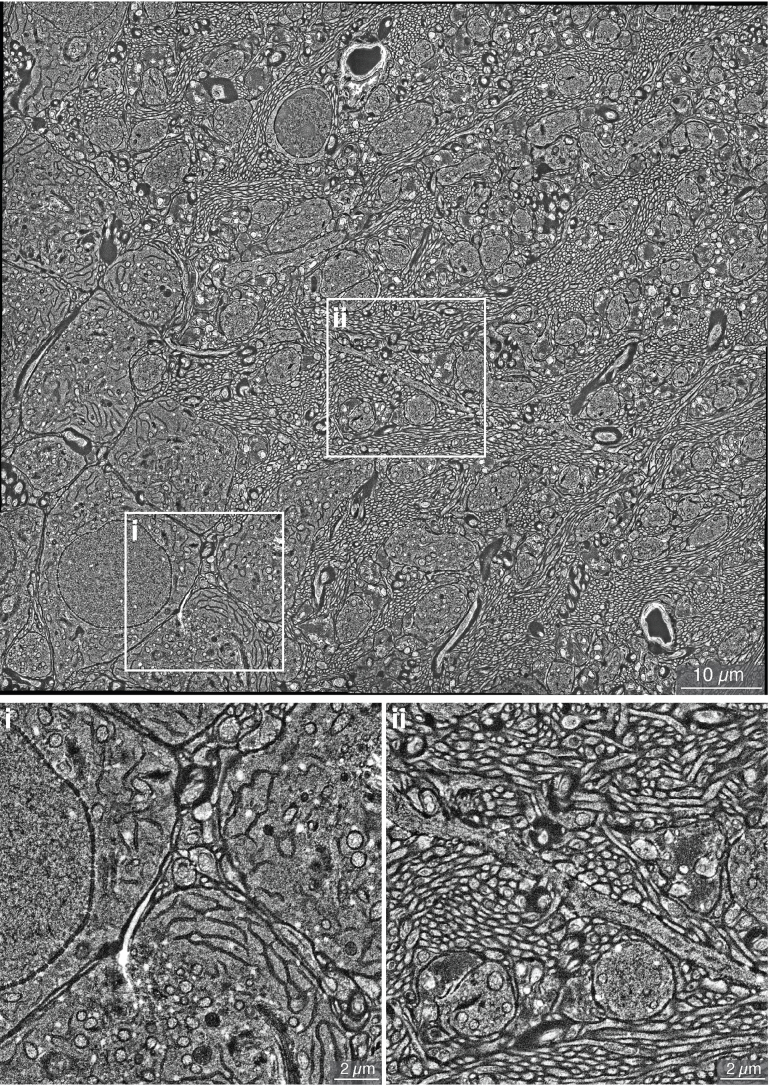
LICONN imaging in the hippocampal CA3 stratum lucidum. Single-plane overview image with magnified views, illustrating the complex arrangement of cellular structures including a primary cilium and intracellular organelle diversity in (i) and mossy fibre bundles and an example of multiple shaft synapses in (ii). Overview imaging in CA3 stratum lucidum was performed in *n* = 2 technical replicates, single tile measurements were performed in multiple technical replicates.

**Extended Data Fig. 8:**
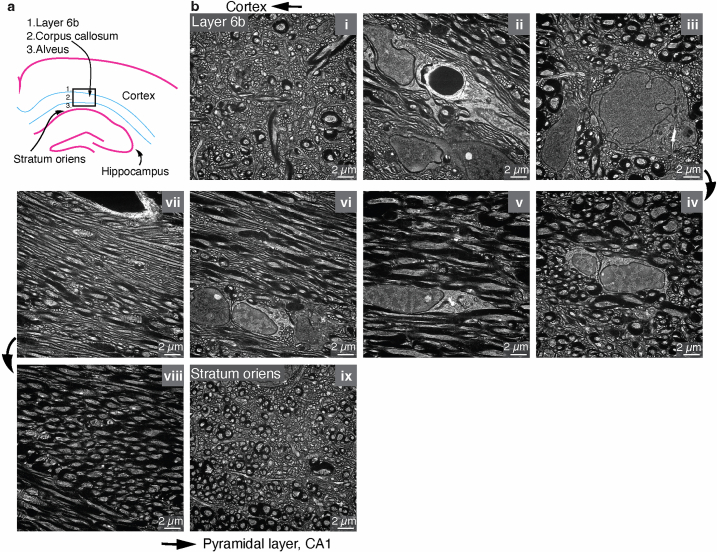
LICONN imaging in white matter. **a**, Schematic of a mouse coronal section and the region of imaging (box). **b**, Single planes from individual LICONN imaging volumes spanning deep cortex, corpus callosum, alveus and hippocampus according to the schematic, revealing the distinct organization in the various regions. Imaging of the various layers was performed in *n* = 1 mouse.

**Extended Data Fig. 9:**
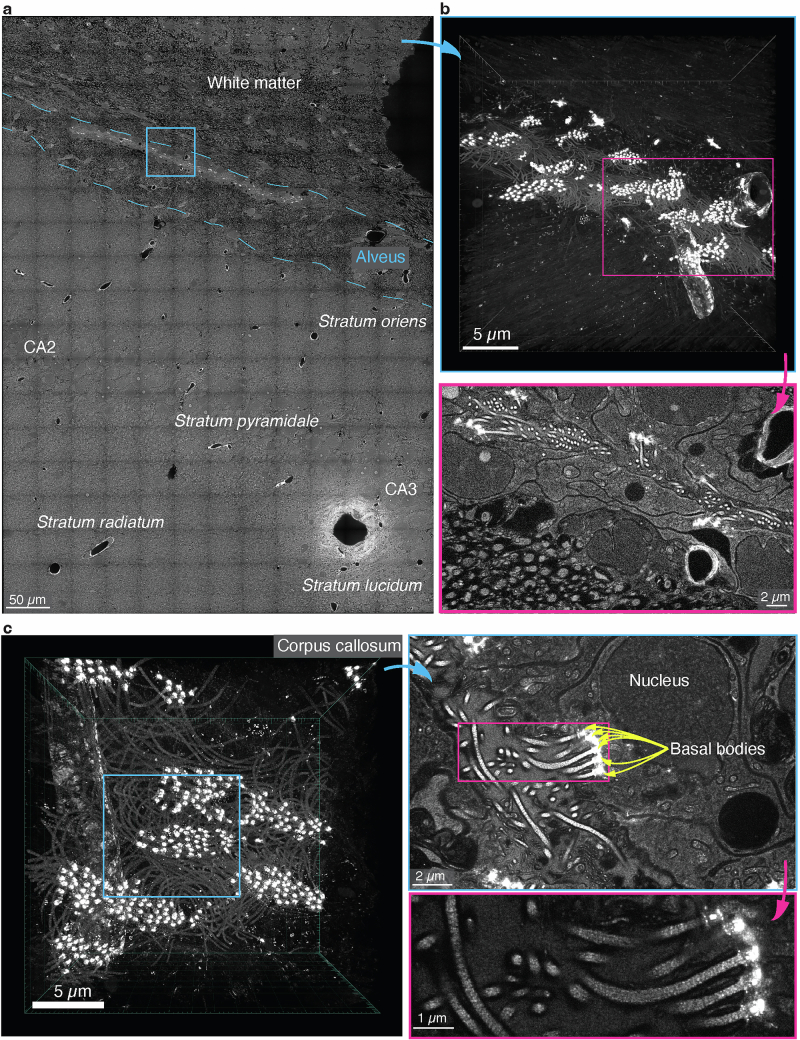
Multi-ciliated cells at the transition between the corpus callosum and the alveus. **a**, Regional overview image. **b,** 3D rendering of a LICONN imaging volume (top, recorded with a 20× objective lens) of the region indicated by the box in the overview, highlighting multi-ciliated cells. The bright structures correspond to basal bodies. Bottom: Single imaging planes from the same volume as indicated by the magenta box in the upper panel. Intensity lookup tables in the individual panels are adjusted to either emphasize the intensely labelled basal bodies or the local cellular architecture. **c**, Left: Rendering of a LICONN imaging volume recorded with a high-NA objective lens in the same region from a different specimen. Right: Single imaging plane showing multi-ciliated cells and magnified view of cilia with intensity lookup table adjusted to visualize the axonemes of cilia. Representative of replicates in *n* = 2 mice.

Finally, we applied LICONN more broadly throughout the brain to assess tissue characteristics beyond synaptic connectivity. We confirmed its applicability in various regions, including the hypothalamus, piriform area and cerebellum (Extended Data Fig. 6), and imaged the characteristic organization of the hippocampal CA3 stratum lucidum (Extended Data Fig. 7). When analysing white matter, including the corpus callosum—a key structure connecting the two hemispheres—and the alveus, we found layers with a high density of myelinated axons (Extended Data Fig. 8). We were intrigued by nearby clusters of cells with multiple prominent processes (Fig. 5h and Extended Data Fig. 9). Investigating their ultrastructure (Fig. 5i), we realized that they corresponded to multiple cilia, originating from basal bodies, again with ninefold internal symmetry. Cilia were prominently highlighted when immunolabelling for acetylated tubulin (Fig. 5h). The microtubule ring had a diameter of 214 ± 27 nm (mean ± s.d.), with individual doublets spaced 63 ± 9 nm apart (Fig. 5i).

## Molecular identification of gap junctions

The ability to map electrical connectivity through gap junctions would be especially useful. This information is typically missing from connectomics datasets, because reliable detection of gap junctions requires particularly high resolution in EM^53^. These intercellular channels contribute to functional network properties in addition to chemical synapses. We immunolabelled connexin-43, a gap-junction protein expressed in astrocytes^54^, and identified astrocytes by the expression of glial fibrillary acidic protein (GFAP) (Fig. 5j,k). LICONN visualized both the electrical connections and the cellular partners. GFAP labelling also molecularly assigned cellular structures bordering capillaries to astrocytes (Extended Data Fig. 5). Immunolabelling of connexin-43 and gephyrin visualized electrical and inhibitory connections. Complementing this with excitatory synaptic connections through deep-learning prediction of bassoon and SHANK2 (Fig. 5l) enabled simultaneous mapping of excitatory, inhibitory and electrical connectivity in the same circuit. Similarly, specific immunolabelling allowed us to determine the density of inhibitory (0.039 ± 0.010 per µm^3^, gephyrin) and electrical (0.054 ± 0.015 per µm^3^, astrocyte-associated connexin-43) connections (four imaging volumes across hippocampus and cortex, two each) (Fig. 5m).

## Lossless axial extension of imaging volumes

**Extended Data Fig. 10:**
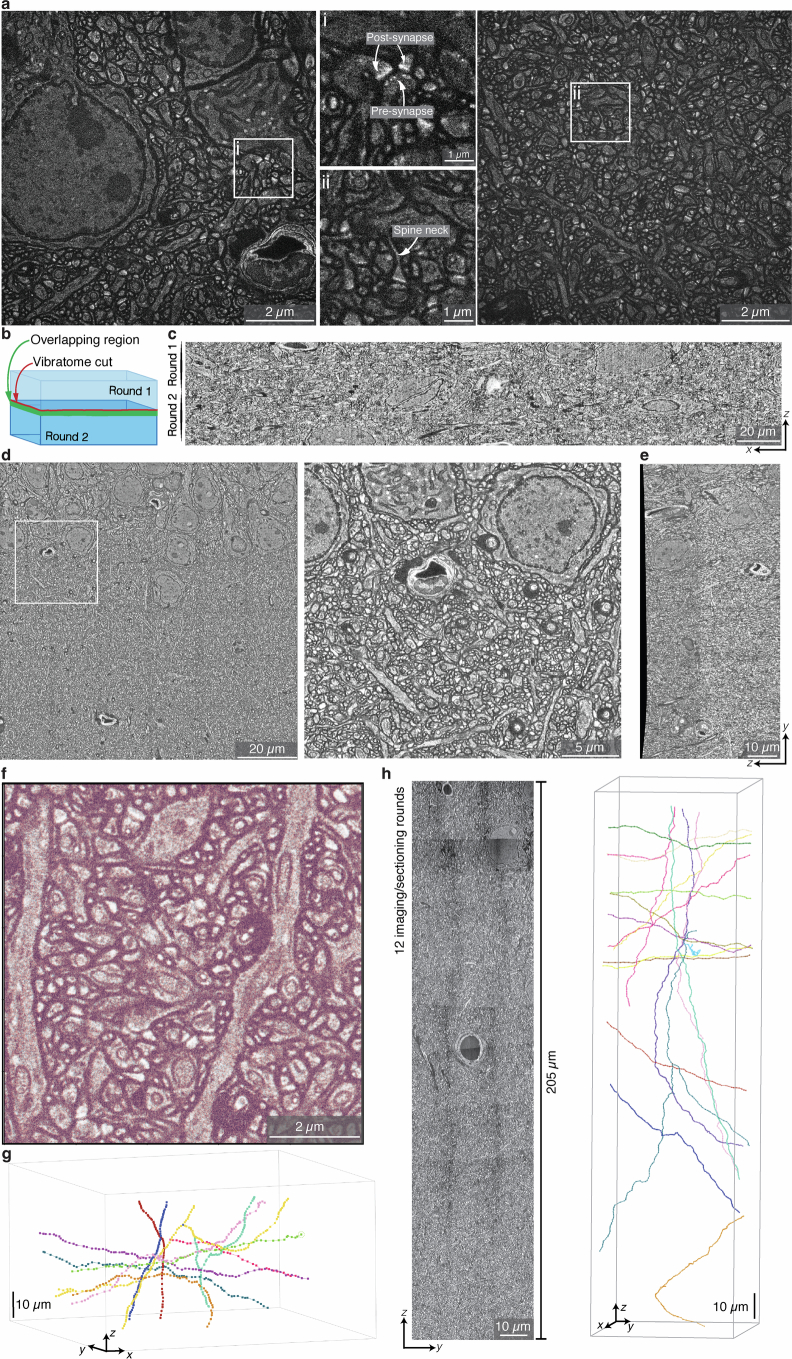
Scaling up in axial direction through iterative imaging and sectioning of LICONN tissue–hydrogel hybrid. **a**, LICONN in 300 µm-thick brain slices (hippocampus, CA1, stratum radiatum). Single imaging planes in subregions of multi-tile volume, with data quality equivalent to thinner tissue sections. Boxed regions: magnified views. **b**, Schematic of iterative block-face imaging/sectioning of LICONN tissue–hydrogel hybrid. In round 1, a multi-tile volume is acquired, then most of the imaged hydrogel layer is removed with a vibratome, and in round 2 a second multi-tile imaging volume deeper in the tissue is acquired. A region of continuous overlap recorded in both imaging rounds enables lossless stitching and fusion of imaging volumes across rounds. **c**, Axial (*xz*-)view of the LICONN volume of panel a, fused from 72 subvolumes arranged on a 6x6x2 3D grid. Data were acquired in two imaging rounds at increasing depth (data displayed after coarse stitching). Axial extent, round 1: 466 µm (30.2 µm native tissue scale) with vibratome cut 320 µm below hydrogel surface. Axial extent, round 2: 353 µm (23 µm native scale). Total multi-round acquisition time: 3 h 36 min (imaging round 1: 2 h 04 min, sample handling and vibratome cutting: 15 min, imaging round 2: 1 h 17 min). **d**, Single-plane overview of the same volume in lateral (*xy*-)direction (109x109 µm^2^ native scale) after volume fusion. Magnified view (boxed region): region with tile borders, showing seamless fusion (region adjacent to view in left image in panel a). **e**, Single-plane axial (*xz*-)view after volume fusion in the same dataset, with seamless fusion across the two imaging rounds. **f**, Single-plane lateral (*xy*-)view of a subarea in the axial overlap region between imaging rounds, after volume fusion. Data from rounds 1 and 2 are colour coded in red and green. Homogeneous colouring indicates voxel-exact matching from individual imaging rounds after fusion. **g**, 3D-illustration of manually traced axons in the final stitched and fused volume, demonstrating traceability across borders between imaging rounds. **h**, Single axial plane of LICONN volume fused from 108 subvolumes arranged on 3x3x12 grid in 3D, acquired in 12 consecutive block-face imaging/sectioning rounds, and 3D-illustration of manually traced axons. Axons extend across tile borders in 3D, from axon entering to axon leaving the imaging volume. Axial extent of final fused volume: 205 µm original tissue scale (7926 optical sections, 400 nm *z*-steps in post-expansion volume). Original data (CLAHE-processed) of final fused volume: https://neuroglancer-demo.appspot.com/#!gs://liconn-public/ng_states/multiround_fusion.json.

To axially extend LICONN volumes beyond the imaging depth accessible with high-NA objective lenses (0.6-mm working distance, in our case), we implemented a block-face imaging method to obtain LICONN volumes built from partially overlapping subvolumes arranged on a 3D grid, allowing seamless fusion without gaps (Extended Data Fig. 10). We first imaged partially overlapping volumetric tiles arranged in two dimensions (2D) to obtain a first slab of LICONN data. We then used a conventional vibratome to slice off most of the imaged hydrogel layer, while placing the cut within the imaged slab. In a subsequent imaging round, we obtained an axially offset multi-tile volume situated more deeply in the tissue, featuring a continuous region of axial overlap with the first multi-tile volume. We then computationally fused the individual imaging volumes in 3D in a voxel-exact manner (Methods), enabling tracing of neuronal structures, including thin axons, across imaging slabs (Extended Data Fig. 10; original data for fused volume: https://neuroglancer-demo.appspot.com/#!gs://liconn-public/ng_states/expid146.json). To demonstrate the potential for further scaling, we iteratively applied this block-face imaging and sectioning approach across 12 rounds, with 108 volumetric imaging tiles arranged on a 3 × 3 × 12 grid in 3D. After voxel-exact fusion, the resulting volume covered 205 µm axially and allowed tracing of neuronal structures across tile borders in 3D (Extended Data Fig. 10 and Supplementary Video 4; see https://neuroglancer-demo.appspot.com/#!gs://liconn-public/ng_states/multiround_fusion.json).

In summary, LICONN is a straightforward technology that directly provides integrated structural and molecular characterization across brain regions, cell types and spatial scales.

## Discussion

The development of connectomics methods has been driven by the ambition of simultaneously achieving dense 3D reconstruction of neurites, synapse-level connectivity, diverse molecular annotations and cost-effective scaling to large volumes (cubic millimetres or more). Successive generations of EM-based technologies have enabled enormous progress^2^, but important limitations remain, particularly in the ability to extract molecular details. By contrast, current light-microscopy techniques for visualizing cellular tissue architecture^19–21,25,32^, including LIONESS^20^ and CATS^21^, do not reach the accuracy and traceability required for connectomic reconstruction. Here we introduce LICONN, which, like EM, enables reliable manual tracing of dendrites, axons and spines (Fig. 1), high-accuracy automated reconstruction of those structures (edge accuracy 92.8%; Fig. 2) and reliable detection of chemical synapses (F1 > 0.9 for immunolabelling- or deep-learning-based detection; Figs. 3, 4). However, unlike EM, LICONN enables direct and simultaneous measurement of spatially resolved molecular information, including specific proteins that reveal chemical synapse subtypes (Fig. 3), electrical synapses and key subcellular features (Fig. 5).

We used LICONN to acquire volumes of up to roughly 1 × 10^6^ μm^3^ (native tissue scale; Fig. 1a), which are similar volumes (albeit with more limited axial extent) to those of previous EM-based connectomics datasets, on which biological analysis of neural circuits has been successfully performed^1,10,47^. We achieved reasonable acquisition rates (6.5-h acquisition time for around 1 × 10^6^  μm^3^ native scale, 0.39 teravoxels, 17 × 10^6^ voxels per s (17 MHz) effective voxel rate including overhead from tile overlap and sample stage positioning) even without optimizations for imaging speed^20^. Within the axial range of the working distance of the objective lens (here 600 µm), simple optical sectioning replaced the ultrathin (nanometre to tens of nanometres) physical sectioning or milling in EM reconstruction. However, the largest EM datasets span a cubic millimetre, and a major long-term goal is mapping the hundreds of cubic millimetres of an entire mouse brain^55^. One promising strategy for scaling LICONN volumes includes hydrogel sectioning to parallelize readout. LICONN hydrogels are mechanically robust, facilitating handling and sectioning. We typically expanded 50-µm-thick slices for experimental convenience, but this does not constitute a fundamental limitation. We expect that scaling LICONN expansion to larger samples will be possible by adjusting denaturation, polymerization and labelling parameters. For example, with slight adaptations for 300-µm-thick sections (Methods and Extended Data Fig. 10), we obtained LICONN imaging volumes that appeared, judged from the structural channel, equivalent in resolution, signal-to-noise ratio and structural preservation to datasets used for extensive validation of traceability. We further showed that consecutive, overlapping imaging volumes in the axial direction can be acquired and computationally fused by removing slabs of the expanded LICONN hydrogel already imaged. Such axial extension of volumes will be required for comprehensive analysis of cellular connectivity, whereas lateral tiling is more straightforward (for example, around 3,000 tiles, approximately 1-mm^2^ native scale in Extended Data Fig. 2).

We have also shown the detection of specific proteins by immunolabelling. Using post-expansion labelling, LICONN benefits from improved epitope access^22,29^ and avoids fluorophore-to-epitope linkage error. Further refinement will expand LICONN’s molecular information content. For example, hydrogel-compatible spatial transcriptomics methods have emerged^56–59^ that measure gene expression directly in tissue. By integrating connectivity with in situ molecular information from individual cells, LICONN presents a viable path towards multimodal descriptions of mammalian brain cells, including morphology, connectivity (including electrical connections), physiology and gene expression^60^.

We further applied LICONN to quantify cellular properties beyond synaptic connectivity, focusing on cilia. Analysing primary cilia in a model of neurodevelopmental disease exemplifies how LICONN can be used to study genotype-to-phenotype relationships and cellular alterations in diseased brains. LICONN was developed to reconstruct arguably the most complex tissue structure, specifically identifying and tracing the finest neuronal processes, such as axons and spines, in the brain; we expect the technology to be broadly useful in other organs and systems in which high-resolution tissue analysis is desirable.

Finally, we note that LICONN is highly accessible. Acquisition is driven by broadly available conventional light-microscopy hardware (here, spinning-disc confocal), and, although LICONN sample preparation introduces new strategies to achieve high-fidelity tissue expansion, the protocol is not fundamentally more complex than previous expansion techniques that have been widely adopted. Deep-learning-based analysis used FFNs and other deep-learning frameworks that have previously been applied in neuronal segmentation and synapse prediction. These and the custom code we developed here are available open source. In conclusion, LICONN forms a technological basis for the routine adoption of connectomic studies in non-specialized neuroscience labs, as well as enabling high-resolution studies in organs other than the brain.

## Methods

### Mice

Animal procedures were performed in accordance with national law (BGBLA 114 and Directive 522), European Directive 2010/63/EU and institutional guidelines for animal experimentation, and were approved by the Austrian Federal Ministry for Education, Science and Research (authorizations BMBWF-V/Sb: 2020-0.363.126, 2021-0.550.199, 2021-0.842.237, 2022-0.121.445 and 2023-0.930.355).

Mice were housed in groups of three to four under controlled laboratory conditions (12:12-h light–dark cycle with lights on at 07:00; 21 ± 1 °C; 55 ± 10% humidity) with food (pellets, 10 mm) and autoclaved water ad libitum. Mice were housed in commercially available individually ventilated cages made from polysulfone with a solid cage floor, dust-free bedding (woodchips) and nesting material.

For all experiments, male and female mice were used interchangeably to demonstrate the technology. Adult mice (aged typically two to three months, unless otherwise noted) were used as indicated with the following genotypes: C57BL/6J wild-type mice, *Thy1-eGFP* (STOCK Tg(Thy1-eGFP)MJrs/J mice, 007788, RRID:IMSR_JAX:007788 hemizygous) and haploinsufficient *Hnrnpu*^*+/−*^ mice (deletion of one allele of *Hnrnpu* spanning axons 4 to 14, generated by crossing the *HnrnpU*^*WT/flox*^ line (Hnrnpu<tm1.1Tman>/J, strain: 032187, RRID: IMSR_JAX:032187) with the *CMV-Cre*^*Cre/Cre*^ line (B6.C-Tg(CMV-cre)1Cgn/J, strain: 006054, RRID: IMSR_JAX:006054).

### Reagents

Chemicals and solutions are listed in Supplementary Tables 1 and 2.

### Transcardial fixative perfusion

Solutions were prepared on the same day and kept at room temperature. Mice were first anaesthetized with isoflurane (1–2% (volume/volume; v/v) and then with ketamine (80–100 mg per kg body weight) and xylazine (10 mg per kg) intraperitoneally, combined with metamizol (200 mg per kg) subcutaneously for analgesia. After checking for deep anaesthesia by toe pinch, they were transcardially perfused at a flow rate of 7 ml min^−1^ using a peristaltic pump, first with room-temperature 1× phosphate-buffered saline (PBS) for 2 min and then with a solution (room temperature) containing 4% (weight/volume; w/v) paraformaldehyde (pH 7.4) and 10% acrylamide (AA, w/v) in 1× PBS for 6 min.

Brains were collected and post-fixed for 8–12 h at 4 °C with gentle agitation, using the same fixative solution.

### Brain tissue processing

Brains were washed three times in cold (4 °C) 1× PBS for around 1 min each, with gentle agitation. They were sectioned coronally at a 50-µm thickness using a vibratome (Leica VT 1200 S). Where indicated, sections were prepared at a 300-µm thickness. Sections were placed in ice-cold 1× PBS supplemented with 100 mM glycine to quench PFA-reactive groups for 6–8 h at 4 °C and washed three times for around 1 min in 4 °C 1× PBS. They were stored at 4 °C in 1× PBS supplemented with 0.015% NaN_3_ for up to three months, typically.

### Epoxide treatment

Brain sections were washed twice for 15 min each in 1× PBS at room temperature with gentle agitation. They were then washed twice for 15 min each in 100 mM sodium bicarbonate in milli-Q water, pH 8.0, at room temperature followed by incubation with 0.1% (w/v) TGE and 0.1% (w/v) GMA in 100 mM sodium bicarbonate in milli-Q water (pH 8.0) for 3 h at 37 °C with gentle agitation in a chemical hood. For 300-µm-thick slices, the TGE and GMA concentration was increased to 1% (w/v). Samples were then washed with 1× PBS for 1 h with gentle agitation at room temperature.

### Pre-expansion immunolabelling for distortion analysis

Pre-expansion immunolabelling was performed only for distortion analysis measurements. All other immunolabellings were performed after expansion. Primary and secondary antibodies are listed in Supplementary Tables 3 and 4. Brain slices were permeabilized for 60–80 min with 0.2% Tween-20 in 1× PBS at room temperature with gentle agitation. Labelling with primary anti-GFP antibody was performed in 5% BSA and 0.2% Tween-20 in 1× PBS overnight at 4 °C with gentle agitation. Samples were then washed three to five times for around 30 min each in 1× PBS at room temperature with gentle agitation, followed by staining with secondary antibody overnight at 4 °C, in the same buffer as for the primary antibody labelling, with gentle agitation, followed by three to five washes for around 30 min each in 1× PBS with gentle agitation. Pre-expansion images were acquired on a spinning-disc confocal microscope after the first gelation step.

### Preparation of hydrogel solutions

The compositions of hydrogels in LICONN are summarized in Supplementary Table 5. For preparation of the first hydrogel solution, AA and sodium acrylate (SA) were mixed in milli-Q water, vortexed and centrifuged at 4,500*g* for 5 min. Supernatant was transferred to a fresh 50-ml tube and supplemented with *N*,*N*′-methylenebisacrylamide (BIS) stock solution (prepared in milli-Q water) to a final BIS concentration of 0.075% w/v. After adjustment to the final volume, solutions were aliquoted in 2-ml tubes and stored at −20 °C, typically for up to one month. Solutions for the stabilizing and second expandable hydrogels were prepared similarly, usually freshly before experiments. We observed that the quality of sodium acrylate was crucial for successful expansion, mirrored in a batch dependency for this specific compound.

### Polymerization of the first expandable hydrogel

In an ice-water bath, the first hydrogel monomer solution (Supplementary Table 5) was supplemented first with 0.001% 4-hydroxy-2,2,6,6-tetramethylpiperidin-1 (TEMPO, w/v) and then with 0.15% ammonium persulfate (APS, w/v) and 0.15% *N*,*N*,*N*′,*N*′-tetramethyl-ethylenediamine (TEMED, volume/volume; v/v) and vortexed. The following sample-handling steps were performed in an ice-water bath. Brain sections were pre-incubated with the hydrogel monomer solution for 30–45 min (75–90 min for 300-µm-thick slices) with gentle agitation in a 24-well plate. A gelation chamber was assembled from two cover glasses on bottom and top and #1 cover-glass strips on either side as spacers (for 300-µm sections: one #1 and one #1.5 cover-glass strip). Gelation was then performed at 37 °C in a humidified chamber for two hours. For optionally acquiring pre-expansion images, the sample was imaged on a spinning-disc confocal microscope (Oxford Instruments, Andor Dragonfly) after the first hour of gelation for around 15 min, and then further incubated at 37 °C for another approximately 45 min.

### Disruption of mechanical cohesiveness, expansion and hydrogel neutralization

After gelation, the top coverslip was removed and the hydrogel–tissue hybrid was trimmed to the region of interest. Together with the bottom coverslip, it was then transferred to a 5-ml beaker with 2 ml denaturation buffer (200 mM SDS, 200 mM NaCl and 50 mM Tris-HCl in milli-Q water, pH adjusted to 9.0 with NaOH). The beaker was placed in a larger vessel containing pre-heated water and transferred to a water bath at 95 °C for 100 min. For 300-µm-thick samples, the denaturation time was extended to four hours, starting at 85 °C and ramping up to 95 °C within about 15 min.

For expansion, samples were placed in milli-Q water, with the water being changed every 20 min (approximately) until no further increase in gel size was observed.

We neutralized unreacted double bonds of the divinyl cross-linker (BIS) remaining after polymerization of the first hydrogel to ensure that it would not react with the following stabilizing and second expandable hydrogels. For this, we incubated with 0.2% APS plus 0.2% TEMED in milli-Q water for 2.5 h at 37 °C with gentle agitation, followed by one washing step with agitation in milli-Q water for 30 min at 37 °C and a similar washing step at room temperature.

### Immunolabelling

Expanded hydrogels were again trimmed to the region of interest and incubated in 1× PBS for 10 min at room temperature, shrinking the gel in a 12-well plate. It was then incubated with primary antibodies (Supplementary Table 3) in 1× PBS at 4 °C overnight with gentle agitation in a 24-well plate, followed by at least three washes for one hour each in 1× PBS with gentle agitation at room temperature. Secondary antibody incubation (Supplementary Table 4) was also performed in 1× PBS at 4 °C overnight with gentle agitation, followed by at least three washes for one hour each in 1× PBS with gentle agitation at room temperature. Post-fixation was conducted for 15 min at room temperature with 4% PFA in 1× PBS, followed by quenching with 100 mM glycine in 1× PBS for 10 min at room temperature with gentle agitation and subsequent washing three times in 1× PBS for around 3 min each. Hydrogels were re-expanded in milli-Q water in a 12-well plate before further processing, and labelling was optionally evaluated by imaging.

During the LICONN procedure, cellular proteins are anchored to the hydrogel and epitopes can undergo denaturation and structural modification. We tested further primary antibodies by imaging directly after the immunolabelling; that is, without applying the second expansion step, as summarized in Supplementary Table 6. Note that not all of the antibodies that produce labelling in the first hydrogel may be retained throughout the entire sample preparation procedure. Dedicated optimization of heat and chemical denaturation and antibody incubation conditions may improve performance for individual antibodies. For somatostatin labelling, we applied a mixture of primary antibodies to increase the signal strength.

### Polymerization of stabilizing hydrogel and neutralization

Expanded hydrogels were pre-incubated on ice-water for 3–3.5 h or 5 h for 50-µm-thick and 300-µm-thick slices, respectively, with the monomer solution of the stabilizing hydrogel (10% (w/v) AA, 0.025% (w/v) BIS, 0.05% (v/v) TEMED and 0.05% (w/v) APS in milli-Q water) with gentle agitation in a 12-well plate. After removing excess monomer solution, the hydrogel was sandwiched between a 22 × 22-mm^2^ coverslip placed on a microscopy slide and an 18-mm round coverslip on top of the hydrogel without spacers, and surrounded by monomer solution. Gelation was then performed for two hours at 37 °C in a pre-warmed (37 °C) humidified chamber. Gels were washed with milli-Q water for around 30–60 min at room temperature.

To neutralize remaining unreacted double bonds of the BIS cross-linker in the stabilizing hydrogel network and ensure that the hydrogel network did not react with the following second expandable hydrogel network, the hydrogel–tissue hybrid was incubated with 0.2% APS and 0.2% TEMED in milli-Q water for 2.5 h at 37 °C with gentle agitation, followed by washing with agitation in milli-Q water for 30 min at 37 °C and a second washing step at room temperature.

### Polymerization of second expandable hydrogel

Stabilized and neutralized hydrogels were pre-incubated on ice-water with the monomer solution for the second expandable hydrogel (19% sodium acrylate, 10% AA, 0.025% (w/v) BIS, 0.05% TEMED and 0.05% APS in milli-Q water) in a 12-well plate for 3–3.5 h (50-µm slices), or 5 h (300-µm slices). After removing excess monomer solution, the hydrogel was again sandwiched between a 22 × 22-mm^2^ coverslip placed on a microscopy slide and an 18 × 18-mm^2^ coverslip on top of the hydrogel without spacers, and surrounded by monomer solution. Gelation was then performed for two hours at 37 °C in a pre-warmed (37 °C) humidified chamber. Hydrogels were washed with 1× PBS for around 30 min at room temperature.

### Protein labelling

Pan-protein staining was performed with either 40 µM ATTO 488 NHS ester or 40 µM Alexa Fluor 488 NHS ester (Supplementary Table 7) in 1× PBS overnight at 4 °C with gentle agitation. Hydrogels were optionally washed three times for one hour in total with 1× PBS, and expanded for up to five hours before imaging in milli-Q water, with fluid exchange approximately every hour.

### Mounting of expanded hydrogels for imaging

Before mounting for imaging, the region of interest was located with the spinning-disc confocal microscope in the expanded hydrogel after placing it onto a plastic culture dish with the bottom replaced with a 50-mm #1 cover-glass. For final imaging, the hydrogel was trimmed to the region of interest. Round coverslips (40 mm #1.5; Bioptechs) were rinsed with milli-Q water, covered with poly-l-lysine (PLL) and incubated at 37 °C for up to three hours. Coated coverslips were stored at 4 °C in 1× PBS for up to one week. Right before mounting, coverslips were washed with milli-Q water and placed in a home-built imaging chamber made from aluminium. The hydrogel was placed in the centre of the coverslip and further stabilized with two-component dental silicone (Twinsil extrahart, Picodent), and immersed in milli-Q water. We imaged for up to eight hours, typically.

### Imaging using spinning-disc confocal microscopy

Imaging was performed on an Andor Dragonfly microscope based on a Nikon Ti2E inverted stand with motorized stage and an Andor Zyla 4.2 Megapixel sCMOS camera (2,048 × 2,048 pixels). Data were acquired using Andor Fusion software v. 2.2. Two pinhole disc patterns (25-µm and 40-µm hole diameter) and four continuous-wave excitation lasers (405 nm, 488 nm, 561 nm and 637 nm) were available. Imaging of non-expanded and expanded samples was performed with a 40× water-immersion objective (Nikon Apochromat LWD 40× lambda S, NA 1.15, water, working distance (WD) 0.6 mm), using the 40-µm disc pattern. For overview imaging (Fig. 5a and Supplementary Figs. 23, 24), a Nikon CFI P-Apochromat 20×, NA 0.95, WD 0.95-mm objective lens was used. The structural channel was imaged with a 488-nm excitation wavelength with a 521/38-nm detection bandpass filter. Typical exposure times were 110–150 ms and the laser power was set to 10–15% of available laser power. Voxels measured 150 × 150 nm^2^ laterally and the axial (*z*-direction) step size was chosen as 200 nm, 300 nm or 400 nm. The lateral field of view (FOV) was typically 307 × 307 µm^2^, corresponding to a biological tissue scale of around 20 × 20 µm^2^. The axial-imaging extent was typically chosen as 20–25 µm in biological tissue scale, limited by the 600-µm WD of the objective lens. For tiled measurements, the ‘field’ mode was used with 10% lateral overlap between individual subvolumes. For additional imaging of immunolabelling channels, colour channels (with excitation wavelengths of 488 nm (structural channel), 637 nm and 561 nm) were recorded sequentially in a frame-wise manner with exposure times of 120–200 ms per immunolabelling channel and typical laser power settings of 10–20%. For detection, 594/43-nm and 685/47-nm bandpass filters were used for the respective colour channels. Images for distortion analysis were acquired with a 150 × 150 × 200-nm^3^ voxel size with the 40×, NA 1.15 water-immersion objective lens.

### Extension of imaging volumes in the *z* direction by block-face imaging and sectioning of expanded hydrogels

To seamlessly stitch imaging volumes not only laterally but also in the axial direction, we performed the following ‘lossless’ sectioning procedure, ensuring overlap of imaging volumes both laterally and axially. We expanded 300-µm-thick brain tissue sections and mounted them on PLL-coated coverslips for imaging as described above. We first imaged a multi-tile volume arranged on a 2D grid. We then removed the hydrogel and twinseal from the imaging chamber and glued the back surface (opposing the imaged layer) of the hydrogel onto the sample holder of a vibratome (Leica VT1200S, vibrating blade microtome) with superglue (Loctite 401, Henkel), gently flattening out the hydrogel by passing a soft plastic sheet over it. The vibratome chamber was partially filled with water, surrounding but not covering the hydrogel. We zeroed the vertical blade position on the hydrogel surface and lowered it to the desired cutting position, chosen to fall within the already imaged region (for example, cutting 320 µm below the hydrogel surface after imaging an axial range of 466-µm physical imaging range in the two-round measurement in Extended Data Fig. 10). Cutting was performed at 0.2 mm s^−1^ blade advancement. We then removed the hydrogel from the vibratome sample holder with the plastic sheet, mounted it for imaging with the vibratome-cut surface facing the PLL-coated coverslip and imaged a second layer of tiled, overlapping imaging volumes similarly to before. For the multi-round measurement, we iteratively repeated this procedure.

### Troubleshooting

When establishing the procedure, we suggest that the protocol is adhered to closely, including details such as the precise chemicals and providers listed, the timing of steps, concentrations, temperatures at which individual steps are performed (for example, on ice-water versus at room temperature) and preparation of fresh solutions directly before experiments where indicated. On the basis of observations made during the development of the procedure (see for example Supplementary Figs. 1–5, 7–9 and Supplementary Table 8), we suggest checking the following points in the case of suboptimal results. Quality of transcardial fixative perfusion is crucial to achieve adequate tissue preservation, including, for example, cell shape. If tissue preservation is suboptimal, basic parameters such as perfusion speed and duration, and composition of solutions, should be checked. We anecdotally observed that when setting the pH of the perfusion solution, large pH variations should be avoided and it was preferable to carefully titrate in one direction until the final pH was reached. If the final expansion factor is lower than expected, over-fixation might be a factor (owing to an excessive duration of the post-fixation step, for example). Similarly, excessive protein anchoring might interfere with full expansion. Variation of cross-linker (BIS) concentration is also expected to have a direct effect on the expansion factor. For sodium acrylate, we observed a batch-to-batch variation in compound quality (for example, impurities), which could have an effect on the final expansion factor and overall outcome. For the other chemicals used, we did not make obvious similar observations. Shortening the time for expansion (for example, one hour instead of four to five hours for the final hydrogel) might also compromise the final expansion factor. Premature gelation of the second or third hydrogel might take place if the same reaction well is used for performing the preceding neutralizing step (removing unreacted double bonds) involving APS and TEMED, even if the well is washed. If the signal-to-noise ratio in the structural labelling is unsatisfactory, protein anchoring and labelling with NHS esters of fluorophores merit scrutiny. NHS esters are prone to hydrolysis. NHS esters of fluorophores should be dissolved only once needed in high-quality (water-free) dimethyl sulfoxide and aliquoted in single-use quantities (10-µl aliquots of 20 mM stock solution stored at −20 °C) to avoid freeze-thaw cycles, and should be used within two to three months. To monitor success at the various steps of the procedure during the set-up phase, it might be useful to perform imaging at various check-points, including after the first expansion step (applying NHS labelling), after immunolabelling and after full expansion.

Observing these guidelines produced highly consistent results. We followed the outcome of all transcardial fixative perfusions performed within a certain time period (*n* = 15 mice) from perfusion to expansion and final imaging. In total, the 15 perfusion experiments yielded 50 experimental runs that typically contained several technical replicates (hydrogel embedding and expansion experiments). In one out of 15 transcardial fixative perfusion experiments, the fixative perfusion itself failed, producing poor tissue preservation in the resulting samples, which is likely to be related to the suboptimal quality of the perfusion solution. In one technical replicate, we observed premature gelation of the stabilizing hydrogel, probably owing to remnants of APS and TEMED in the same well plate used during the neutralization step. The other replicates produced useful imaging data either for demonstrating various technical aspects (for example, determining the expansion factor, distortion analysis and testing of immunolabelling) or for the full LICONN procedure, as required. We selected a subset of these for further analysis and display, on the basis of visual inspection for factors such as the signal-to-noise ratio.

### Data handling and format conversion

Raw imaging data were obtained in IMS format, as generated by the spinning-disc confocal microscope (Andor Dragonfly), which is a hierarchical data format with six resolution levels, from which we extracted the highest resolution level (level 0). Data conversion for downstream analysis was performed with custom Python scripts implemented in Python v.3.8 or higher, including the Imaris-ims-file-reader, zarr, webKnossos and tifffile packages. Custom scripts are available at https://github.com/danzllab/LICONN, with individual package versions specified in the respective configuration (.yml) files. Datasets are available through Neuroglancer (see Data availability section) or through the data repository of the Institute of Science and Technology Austria (ISTA)^61^ at https://research-explorer.ista.ac.at/record/18697. At various processing steps, conversion of file formats was necessary, and this was performed on ISTA’s high-performance computing cluster, using either a single node or SLURM (slurm-wlm 22.05.8) for task allocation, according to the resource requirement based on file size. We transferred files to cluster storage and converted from the respective source format to tiff, zarr, n5 or webKnossos (wk) format according to processing needs.

### Image processing

Imaging data were processed using Fiji v.1.54f, including the CLAHE, BigWarp and BigData viewer plug-ins. For visualization, display ranges were adjusted, also accounting for a camera background signal of around 100. Intensity lookup tables were linear unless otherwise noted.

Overlay of immunolabellings with LICONN data was performed in GIMP v.2.10.34 after saving individual channels separately in RGB format in Fiji. The background in immunolabellings was set to transparent (alpha = black) and immunolabellings were overlaid with the structural channel. The output was saved in PNG format.

CLAHE (contrast-limited adaptive histogram equalization) was applied as indicated for individual figure panels, using either Fiji or a Python-based workflow as described below. In Fiji, first the display range was adjusted, and in the CLAHE plug-in the block size and histogram bin were set to the minimum and maximum values of the display range. The maximum slope was set to 3 and the standard (not fast) processing option was chosen. Three-dimensional renderings of cilia were done with Imaris v.9.3.

### Distortion analysis and calculation of expansion factors

The analysis of distortions in the expansion procedure was adapted from previously published methods^39,62^, and the code was adapted from https://github.com/Yujie-S/Click-ExM_data_process_and_example. We analysed a total of 14 imaging volumes of around 20 × 20 µm^2^ laterally and 9–15 µm axially, recorded across 4 technical replicates in *n* = 3 *Thy1-eGFP* mice, exhibiting cytosolic expression of eGFP in a sparse subset of neurons. Imaging volumes corresponded to single tile measurements (no stitching or fusion of subvolumes). For distortion analysis, immunolabelling against eGFP was performed before subjecting the samples to the LICONN expansion procedure, and the eGFP channel was used for analysis. Pre-expansion images were acquired after around one hour of polymerization of the first hydrogel. Imaging volumes containing the same structures were acquired after expansion of the first hydrogel (before application of the stabilizing hydrogel) and after expansion of the second expandable hydrogel, with a spinning-disc confocal microscope and a 40×, NA 1.15 water-immersion objective lens, as in the LICONN imaging. This allowed us to assess distortions beyond the approximately 200-nm spatial scale. Using the BigWarp^63^ plug-in in Fiji, we first manually placed around 25 landmarks at corresponding neuronal features in maximum intensity projections of the respective pre- and post-expansion imaging volumes. Using BigWarp, we then applied either a similarity transformation (including isotropic scaling, translation and rotation) or an affine transformation (including scaling, translation, rotation and shearing) to the pre-expansion images to best match landmarks in pre- and post-expansion images. Expansion factors were extracted as the linear scale factor in the similarity transformations. We smoothed pre- and post-expansion projection images with Gaussian filters of different widths to account for the different resolution in the respective images. For individual expansion steps, *σ* was set to 1 and 6 pixels for pre- and post-expansion images, respectively. For total (iterative) expansion, *σ* was set to 1 and 16 for pre- and post-expansion images, respectively, resulting in a comparable appearance as judged by visual inspection. For one of the datasets, the intensity range was slightly saturated at the main dendrite branch in the pre-expansion image. We now used the imregdemons.m function in MATLAB (v.R2022b, MathWorks) to compute distortion vector fields^62,64^ from the aligned pre- and post-expansion images. For this, we used the images before the respective expansion step as a reference and evaluated distortions arising from that expansion step. For display purposes, we used the quiver function in MATLAB with vector scaling set to 1.5. We then computed and plotted distortions (measurement error) for different measurement lengths, as previously described^16^. For this, we generated binary masks of neuronal structures of interest by thresholding pre- and post-expansion images, and then combining, dilating and eroding the resulting masks using parameters as judged by visual inspection. We then randomly sampled 2 × 10^5^ pairs of points restricted to the masked area of interest. These pairs of points defined a set of vectors in the pre-expansion image. We then found a corresponding set of transformed vectors by applying the distortion field generated with imregdemons.m to the respective pairs of points. For each measurement length, we then computed the measurement errors as lengths of the difference vectors between the pre-expansion and transformed vectors combined from three or four different datasets within one experiment. For plotting, measurement errors at a given measurement length were averaged and the mean was plotted together with the standard deviation.

From these measurement errors, the root mean square (RMS) error was calculated across different measurement scales. Finally, the mean and standard deviation of RMS errors across the *n* = 4 technical replicates were plotted for individual expansion cycles and transformations. For display purposes, we smoothed the measurement error curve with a one-dimensional median filter.

### Stitching and fusion of imaging tiles

We extended SOFIMA (https://github.com/google-research/sofima, git hash 64d5c7c) to support seamless stitching of 3D tiles. In brief, we processed every volumetric tile with CLAHE, applied independently to every in-plane (*xy*) section of the tile. The tiles had an in-plane size of 2,048 × 2,048 voxel^2^ and an axial extent varying depending on the experiment. We laid out the tiles on a 2D grid according to imaging metadata, and used a coarse positioning step to find their initial location in the coordinate system of the output volume. This was followed by fine alignment to elastically deform them to obtain seamless matches between adjacent tiles and form 3D sections, around 20–30-μm thick. The tiles were then rendered with linear-distance-weighted blending of overlapping image content.

For the coarse positioning step, we extracted a small (50-voxel)^3^ cube of image content from the ‘preceding’ tile of every adjacent tile pair, and used cross-correlation to identify the corresponding image content in the ‘following’ tile, forming an offset vector between the tiles. The tiles were then modelled as unit masses connected by springs of resting lengths equal to the computed offsets, and the system was allowed to relax, with the relaxed state determining the tile positions at the beginning of the fine alignment step.

For fine alignment, we modelled every tile as an elastic mesh of unit masses placed on a regular grid with a 3D stride of 20 voxels and nearest neighbour masses connected with Hookean springs. Coarse positioning resulted in the neighbouring tiles partially overlapping with each other, and for any mass located in the overlapping image region, we again used cross-correlation to identify the position within the neighbouring tile of a an (80 voxel)^3^ image patch centred on the mass (PATCH_SIZE=80). These formed point correspondences between the tiles, which we modelled as 0-length Hookean springs, and allowed the whole system to relax. The relaxed set of tile meshes was used to render the aligned volume.

For datasets comprising more than one thick section (Extended Data Fig. 10), we first stitched tiles into thick sections as described above, and then aligned them to each other sequentially. First, we manually identified the approximate amount of overlap in the axial dimension (typically 100–200 voxels) for adjacent sections, and computed a flow field from the middle plane of the overlap subvolume of the following section to the overlap subvolume of the preceding section, using a patch size of (80, 80, 80) and a stride of (40, 40, 40) voxels in the following section, and a patch size of (160, 160, overlap depth) in the preceding section.

We then modelled the following section as a 3D spring mesh, and allowed it to relax according to the flow field. The relaxed mesh was used to render the following section in alignment with the preceding one. We tracked the position of the middle plane of the following section for which the flow field was computed as the thick section was warped, and when fusing it with the preceding section, retained voxels from the preceding section above (lower axial coordinates) this warped plane, and used voxels from the following section below it. This is similar to the ‘fine alignment’ of individual tiles, and the overall strategy is a direct 3D analogue of how alignment of 2D sections is done with SOFIMA for volume EM datasets.

Details of processing were as follows. In all cases, we based stitching and fusion on the structural imaging channel. We first transferred individual raw IMS files (uint16 format) to cluster storage and extracted the highest resolution level (level 0). We then used a BigStitcher-based Fiji macro to concatenate the data and convert to n5 format. We then applied CLAHE tile-by-tile with Python. Data handling was done with the TensorStore library (https://google.github.io/tensorstore/, v.0.1.33). We used the numpy.clip function to clip the intensity range plane by plane typically between 120 and 350, determined by visual inspection. For CLAHE, we used skimage.exposure.equalize_adapthist to apply histogram equalization, with a clip limit of 0.03. We multiplied the output (ranging from 0 to 1) with 255 and saved as uint8 format.

In the rigid, coarse alignment step, we determined the relative arrangement of tile pairs inputting the known tile layout (arrangement of tiles in the image acquisition). We set the QUERY_R_ORTHO and QUERY_R_OVERLAP parameters to 25. We set the minimum tile overlap (QUERY_OVERLAP_OFFSET) to 60 (100 for the dataset in Fig. 2a) and the size of the search area within tiles (SEARCH_OVERLAP, SEARCH_R_ORTHO) to 300 (400 and 600, respectively, for the dataset in Fig. 2a). The second, fine-stitching, step was used to warp tiles and achieve a smooth transition at tile borders, using a stride of (20, 20, 20) voxels. This step outputs a mesh that ensures a globally consistent warping and relative arrangement of individual tiles. In the last step, we applied the obtained mesh to either the CLAHE-modified structural imaging data for downstream (automated) segmentation or skeletonization, or the raw structural imaging data, as well as to immunolabelling channels, resulting in the respective fused imaging volumes in n5 format. We performed these processing steps on ISTA’s high-performance computing cluster and/or, in the case of the measurement in Fig. 2a and Extended Data Fig. 10, on Google’s high-performance computing infrastructure, requesting resources according to the processing task. Typically, for CLAHE, we requested 16 CPUs (Intel Xeon CPU E5–2680 v4, 2.40 GHz or similar) and 150 GB of RAM. For coarse stitching, we typically requested 3 GPUs (GeForce RTX 2080 Ti or similar), 32 CPUs and 100 GB or RAM. Fine stitching was done with 1 GPU, 16 CPUs and up to 970 GB of RAM. Rendering was typically done requesting 32 CPUs and up to 700 GB of RAM.

### Manual segmentation and proofreading

For manual segmentation, we used VAST v.1.4.0 (downloaded from https://lichtman.rc.fas.harvard.edu/vast/) with the data in eight-bit format. Segmentation of the data in Fig. 1g was originally performed by one person, and the resulting segmentation was used for training. For visualization in Fig. 1g, segmentation was partially proofread by another person using VAST v.1.4.1.

### Skeletonization

We used webKnossos v.22.05.1 for manual skeletonization, running on a local computer with 2× AMD EPIC MILAN 75F3 processor, 32-core 32C/64T, 2.95 GHz with 512 GB of memory, 2 TB NVMe-SSD and an additional 27 TB NVMe-SSD, as well as 4 NVIDIA A6000 GPUs. To reduce required memory resources, we converted data to uint8 format when converting to the webKnossos (wk) format. Before conversion, we clipped the intensity range according to visual inspection, to account for intensity outliers and avoid signal overflow. For convenient exploration of the datasets across spatial scales, we made use of ten precomputed resolution levels, using the webKnossos downsample function. Typically, raw data rather than CLAHE-processed data were used for tracing. When comparing automated segmentation of the volume in Fig. 2a with manually generated skeletons, we checked sites of discrepancy and corrected skeletons as appropriate.

### Generation of GFP-based ground truth

For testing traceability by comparison with an independently generated ground truth, we compared manual tracings of neurites in the structural LICONN channel with their structure revealed by cytosolic expression of eGFP in *Thy1-EGFP* mice. The LICONN structural channel and the eGFP channel were recorded at a similar spatial resolution after tissue expansion with a spinning-disc confocal microscope. The structural channel was recorded in the 488-nm excitation channel, whereas eGFP was detected by post-expansion immunolabelling as described above with imaging at 561 nm excitation. Voxel sizes were either 150 × 150 × 200 nm^3^ or 150 × 150 × 300 nm^3^, with the largest step size referring to the *z* direction. Data were converted to wk format for tracing. Using the webKnossos access permissions feature, we assigned permissions to ensure blinding as required. For generating ‘ground-truth’ skeletons of eGFP-expressing neurons, both the structural and the eGFP channels were loaded and considered jointly. In addition, one seed point was placed in each eGFP-expressing neuronal structure. Two novice human tracers were trained in a non-blinded manner with both the eGFP and the structural LICONN channels of four datasets (single volumetric imaging tiles, around 19 × 19 × 19 µm^3^), containing, in total, two eGFP-expressing axons and two eGFP-expressing dendrite stretches. These datasets were excluded from further analysis. After training, the LICONN channel together with the seed points were provided to the two human annotators blinded to the eGFP channel who independently traced the marked structures based on the structural channel.

For dendritic spines, annotators were asked to indicate these as short branches diverging from the main dendrite trunk. The annotators were then asked to compare the individual skeletons and generate a ‘consensus’ skeleton for each of the traced structures. Human consensus skeletons were then overlaid with and compared to the eGFP-based ground truth using webKnossos.

### Neuronal instance segmentation

We used FFNs (available at https://github.com/google/ffn, git hash 12d680e)^42^ to automatically segment the datasets. We used the original convolutional stack architecture with a 33 × 33 × 33-voxel FOV, a step size of 8 voxels and the network depth extended to 20 residual modules. The models were applied to data with 19.4 × 19.4 × 25.9-nm^3^ voxel size (in original tissue scale). For this, CLAHE-processed volumetric imaging data were 2× downsampled laterally relative to the imaging voxel size using volume averaging in Python. Axially, datasets with a 200-nm axial-imaging step size (effective voxel size 9.7 × 9.7 ×13.0 nm^3^, obtained by scaling with an exF of 15.44; Fig. 2a) were also downsampled twofold in the *z* direction, whereas datasets with a 400-nm axial step size (effective voxel size 9.7 × 9.7 × 25.9 nm^3^; Fig. 1a) were not downsampled in the *z* direction. Training was performed with a batch size of 128 and a learning rate of 10^*−*4^ using the AdamW^65^ optimizer with 32 NVIDIA V100 GPUs, for up to 30 days, which corresponded to about 8.8 million training steps. A Jupyter notebook for performing FFN inference on LICONN data is available at https://github.com/google/ffn/blob/master/notebooks/ (git hash: 12d680e).

We used an iterative approach to collect the volumetric ground-truth annotations for training the FFNs. First, 162 neurite fragments covering 110 Mvx (megavoxels, at full imaging resolution) were manually annotated as described above, using VAST in the dataset shown in Fig. 1g (single tile). Model 1 trained with these data on full-resolution imaging data was used to segment the multi-tile volume in Fig. 2a. We then manually agglomerated 103 neurite fragments covering 447 Mvx (voxel number referring to full imaging resolution) within that segmentation (for a description of manual agglomeration, see below). We used this to train another FFN model (model 2), using both the newly collected data and the pre-existing manually painted annotations for training with voxels 2× downsampled in every direction (19.4 × 19.4 × 25.9-nm^3^ effective voxel size). Within the segmentation results of model 2, we manually proofread a larger set of axons (540 axons, 920 Mvx at full imaging resolution, 115 Mvx downsampled, 17.8-mm cumulative path length) and dendrites (314 dendrites, 3,344 Mvx at full imaging resolution, 418 Mvx downsampled, 23.8-mm cumulative path length), and used these data to train the final (model 3) FFN model that was used to produce all the automated neurite segmentations in the manuscript.

We followed a segmentation strategy optimized for first reducing the number of segments affected by merge errors (‘base segmentation’) and then agglomerating these mostly merge-free supervoxels into larger neurites, as described in the following sections. For the base segmentation, we applied the seed-order oversegmentation consensus procedure^42^, followed by oversegmentation consensus with two more segmentations generated with alternative FFN check-points (snapshots of weights saved at regular time intervals during training), which were manually selected for having a small number of wrong merges. This reduced the number of falsely merged supervoxels in the segments evaluated with manually generated skeletons in the volume in Fig. 2a to zero, whereas the overall volume contained at least five mergers. For determining edge accuracy^42^, a skeleton edge was counted as ‘accurate’ when both nodes were labelled identically and the label did not correspond to a segment overlapping more than one skeleton.

We automatically skeletonized the base segmentation for downstream use in agglomeration and proofreading using the TEASAR algorithm^66^ (https://github.com/seung-lab/kimimaro, version b390a9abdde60ec81472f1901e7980a962ecd847) and heuristically identified all nodes of degree 1 in the resulting skeleton graphs as ‘end-points’.

### Semantic segmentation

To automatically classify segments into distinct subclasses—in particular, axons and dendrites—we developed a semantic segmentation pipeline.

For this, an experimenter classified manually agglomerated objects in a segmentation of a 512 × 512 × 334-voxel subvolume (at the downsampled 19.4 × 19.4 × 25.9-nm^3^ effective voxel size) of the dataset in Fig. 2a into one of five classes: axons (331 segments, 15 Mvx), dendrites (36 segments, 11.1 Mvx), myelinated axons (6 segments, 1.1 Mvx), oligodendrocytes (2 segments, 0.15 Mvx) and (astro)glia (47 fragments, 1.3 Mvx). We used these annotations to train a neural network model to perform semantic segmentation (voxel-wise multi-class prediction) of the structural LICONN data. The neural network used the same residual 3D convolution stack architecture as the FFN, with a depth of eight residual modules and a FOV of 33 × 33 × 33 voxels, but with the convolutions operating in ‘valid’ mode, and the residual summation discarding context at the boundary of the larger-sized argument. The model was trained with asynchronous stochastic gradient descent at a learning rate of 10^−4^, using 16 NVIDIA V100 GPUs and a batch size of 16. Training lasted approximately 38 h, during which 40.7 million steps were executed. Training points from all five classes were sampled at equal frequencies.

We used the trained model to generate a semantic segmentation of the entire volume in Fig. 2a and associate every voxel with the class predicted with the highest probability. These voxel-wise classifications were then aggregated over all voxels of every instance segment, and the class predicted most frequently was associated with that segment.

### Segment agglomeration

The instance segmentation pipeline described above is optimized to make it unlikely that an individual super-voxel incorrectly covers more than one neurite. This happens at the cost of an increased number of split errors; that is, assigning multiple smaller segments to the same neurite.

To mitigate this, we applied a multistep agglomeration procedure in which the FFN model was used to evaluate whether a pair of geometrically proximal segments should be merged together^42^ and how a segment could be further extended from a heuristically detected end-point in the skeletons generated from the base segmentation^9^. In the first step, we used the ‘relaxed acceptance criterion’^9^. In the second step, we merged two segments when the end-point-seeded FFN inference recovered 90% of the voxels of both segments within a (200 voxel)^3^ subvolume centred at the end-point.

We further trained two FFN models with a 3D U-net architecture and a 128^3^-voxel FOV and without a FOV movement policy. We used the same training data as for the main instance segmentation model, but split the data into two non-overlapping sets, so that one model was trained exclusively on axons, and the other exclusively on dendrites. We applied the axon model to end-points of all axon segments. Within the prediction results for every end-point, we determined the segment *S* with the highest recovery fraction (other than the segment *E* containing the end-point) and agglomerated *S* and *E* if that fraction was at least 50%, and if *S* had an end-point within 100 voxels of the end-point of *E*. We applied the dendrite model to all skeleton nodes of dendritic trunks (skeletons generated from the base segmentation), taking any dendritic segment of at least 100,000 voxels to be a trunk. For every evaluated point, we collected any putative spine (dendritic segment of less than 100,000 voxels) associated with at least a 50% recovery fraction. We then agglomerated all spines to the trunk for which it had the highest recovery fraction.

The agglomeration procedure resulted in a graph with 516,437 edges, in which each edge represents one pair of automatically joined base segments.

### Agglomeration graph filtering

The semantic segmentation model allowed us to associate per-class voxel counts with all base and agglomerated segments. We used these classifications to filter the agglomeration graph similarly to previous work^9^. Specifically, we sorted the edge list in decreasing order of the associated scores, and discarded any edge that would cause a merge between inconsistent classes. The class combinations we considered were: (astro)glia versus axons versus dendrites versus oligodendrocytes; (astro)glia and oligodendrocytes versus others; and (astro)glia and oligodendrocytes versus axons versus dendrites. An (agglomerated) segment was considered to belong to one of these composite classes when it had a total size of at least 100,000 voxels, and at least 75% of its voxels were classified as the target class. This criterion was relaxed to 10,000 voxels and 50%, respectively, for the end-point agglomeration step, in which we also disallowed merging together objects larger than 100,000 voxels.

### Manual proofreading of neuron segmentations

The automated segmentation of the dataset obtained as a result of the agglomeration procedure was expected to have remaining split and merge errors, which we decided to correct in a structured manual proofreading workflow, leading to a marked increase in proofreading speed. Focusing on topological split and merge errors rather than painting voxels, the workflow was implemented as a Python script driving the Neuroglancer viewer^67^ (https://github.com/google/neuroglancer, c466b24), within which an expert annotator interactively inspected segments and the structural channel images in both 2D cross-sections and as 3D mesh rendering. The tool had features for automatically moving the 3D cursor to predetermined locations (such as end-points of segments), marking segments for future view and directly editing the agglomeration graph by adding or removing edges.

Using the results of the semantic segmentation to classify all agglomerated segments, we organized the proofreading process as follows:

1. All axon segments (*n* = 2,674) of at least 10,000 voxels and touching a single face of the bounding box of the volume were inspected for splits and manually connected to appropriate partner segments where necessary. The tool was configured to automatically navigate to heuristically detected end-points of the axon segments.
2. All axons within the volume (*n* = 18,667) were reviewed for shape implausibilities resulting from putative merge errors, which were then inspected and corrected.
3. Dendritic spines not connected to a trunk (*n* = 2,782) were reviewed and connected to a trunk when one could be unambiguously identified.
4. All dendritic branches (*n* = 1,643) were reviewed for incorrectly associated spines, which were separated, and subsequently manually connected to the correct trunk.

### Synapse detection through immunolabelling

To automatically detect pre- and postsynaptic sites in volumetric datasets comprising the LICONN structural channel and immunolabelling channels for bassoon and SHANK2, respectively, we loaded the multicolour image stacks in tiff format. We converted image stacks for molecular signals to 32-bit floating numbers and adjusted the contrast to a minimum of 101 to account for camera background, and the maximum to the 99.5th and 99th percentile for the dataset in Fig. 4l and Supplementary Fig. 19g–i (300-nm *z*-step size) and 99.95th and 99th percentile for the datasets in Figs. 3g,h and 4p (200-nm or 400-nm *z*-step size) for bassoon and SHANK2, respectively. We then applied background subtraction by applying Gaussian filters of two different widths using scipy.ndimage.gaussian_filter, with *σ* = 5 voxels and *σ* = 11 voxels, corresponding to signal and background, respectively, for bassoon (*σ* = 6 voxels and *σ* = 10 voxels for datasets with 200-nm *z*-step size) and *σ* = 4 voxels and *σ* = 11 voxels (*σ* = 6 voxels and *σ* = 12 voxels for datasets with 200-nm and 400-nm *z*-step size) for SHANK2. Background was subtracted from signal and negative values were set to zero. We then applied Otsu thresholding and used the skimage.measure.label function for converting the resulting binary mask into instance segmentations. We then applied the regionprops function to extract the maximum intensity value for the underlying LICONN channel for each of the 3D regions defined by the individual segments. The list of maximum intensity values had a bimodal distribution, which we used to distinguish between synaptic regions (high intensity in structural channel) and unspecific immunolabelling (low intensity in structural channel). To separate the two populations, we again used Otsu thresholding. To remove further non-specific detections, we performed additional size filtering. Segments that occupied less than 60 voxels were removed, as well as objects that spanned fewer than a certain number of planes in the *z* direction (300-nm *z*-step size: <13; 200-nm *z*-step size: <14 for bassoon and <12 for SHANK2; 400-nm *z*-step size: <12 for SHANK2). Listed parameters were determined from a grid search on a separate validation dataset or a subvolume of the analysed dataset for each of the *z*-step sizes, choosing the parameter set that resulted in the highest F1 score.

We finally converted the individual segments into point annotations, using the regionprops function to find the weighted centroid for each segment based on the underlying LICONN data.

For detecting gephyrin-positive post-synapses, we applied a simplified algorithm involving background removal, Otsu thresholding, morphological opening and size filtering, and manually proofread the output.

### Validation of immunostaining-based pre- and post-synapse detection

For validating automated synapse detection, a human annotator created ground-truth synapse annotation in a dataset comprising the LICONN structural data, bassoon immunolabelling and SHANK2 immunolabelling. For each synapse, they generated a ‘skeleton’ with the source point in the pre-synapse and the target point in the post-synapse, near the respective centres of the molecular signals, using webKnossos v.22.05.1, after conversion of the datasets to wk format. Bassoon signals without corresponding SHANK2 signals, which are likely, in large part, to correspond to inhibitory synapses, were annotated as single source points. Similarly, at volume edges, instances of SHANK2 signals without corresponding bassoon signals occurred, which were annotated as single target points. The human annotator also indicated synaptic connections that lacked SHANK2 signal but showed a clear PSD in the structural channel as excitatory post-synapse. The special cases of a single post-synapse connected to more than one pre-synapse or a single pre-synapse connected to multiple post-synapses were handled by text comments. In total, three datasets were annotated. One test dataset from the hippocampus had a 300-nm *z*-step size and comprised 700 × 700 × 200 voxels of size 9.7 × 9.7 × 19.4 nm^3^. This dataset contained 261 pre-synapses and 247 post-synapses, forming 242 full synapses (with one-to-many connections contributing a count of 1 with each edge), with 25 occurrences of unpaired pre-synapses and 7 unpaired post-synapses. The second test dataset from the hippocampus had a 200-nm *z*-step size and comprised 1,000 × 1,000 × 600 voxels of size 9.7 × 9.7 × 13.0 nm^3^. This dataset contained 1,116 pre-synapses and 1,084 post-synapses, forming 1,059 full synapses (with one-to-many connections contributing a count of 1 with each edge), with 57 occurrences of unpaired pre-synapses and 25 unpaired post-synapses. The third test dataset was from cortex, had a 300-nm *z*-step size and comprised 1,322 × 1,322 × 170 voxels of size 9.7 × 9.7 × 19.4 nm^3^. This dataset contained 306 pre-synapses and 262 post-synapses, forming 261 full synapses (with one-to-many connections contributing a count of 1 with each edge), with 48 occurrences of unpaired pre-synapses and one unpaired post-synapse. We generated further validation datasets for parameter tuning, which were not included in the test data.

To compare the automatically generated point annotations of bassoon or SHANK2 signals with the corresponding human ground-truth point annotations, we performed spatial matching of the respective point annotations. For this, we defined a cost matrix with entry *C*_*ij*_ corresponding to the Euclidean distance between automatically generated point annotation *i* and manually generated point annotation *j*. We defined a matching limit *r*_matching_; that is, an upper distance beyond which matching was not allowed, and set it to *r*_matching_ = 30 voxels after stepping this parameter in a test dataset. For *C*_*ij*_ > *r*_matching_, we set this matrix entry to an arbitrary large number. We now used the scipy.optimize.linear_sum_assignment function to solve the linear assignment problem of finding corresponding points; that is, the single-point to single-point matches with minimum Euclidean distances. Pairs matching between automatically and manually generated annotations were classified as true-positive automated detections. Automated detections without corresponding ground-truth element within the matching radius *r*_matching_ were classified as false-positive detections. Ground-truth detections without a corresponding match in the automated detection were classified as false-negative detections.

### Association to full synapses

To connect corresponding presynaptic sites automatically identified by bassoon immunolabelling with postsynaptic sites automatically identified by SHANK2 immunolabelling, we first defined a cost matrix with entries *S*_*ij*_ corresponding to the Euclidean distances between presynaptic annotations *j* and postsynaptic annotations *i*. We then defined a binary matching matrix, *M*_*ij*_, with *M*_*ij*_ = 1 indicating a connection between the respective pre- and postsynaptic sites (else 0).

Solving this as a linear assignment problem as above would result exclusively in 1:1 matches, which would not capture cases in which one pre-synapse is connected to multiple post-synapses or vice versa. We therefore developed a pipeline that gave priority to the spatially closest occurrences of bassoon and SHANK2 during the presynaptic–postsynaptic pairing procedure but that also allowed for one-to-many connections.

In a first pass, we paired every postsynaptic site with the closest presynaptic sites; that is, we identified the smallest *S*_*ij*_ within each row of the cost matrix and set the corresponding *M*_*ij*_ to 1 if *S*_*ij*_ was below a maximum matching distance of *d*_matching_ = 30 voxels. This value was taken from the distance distribution between manually generated pre- and postsynaptic annotations, choosing a value close to the observed maximum distance of 31 voxels.

Hence, each post-synapse was at most associated with one pre-synapse, whereas each pre-synapse could be associated to zero, one or more post-synapses. In a second pass, we now exclusively considered those bassoon signals that were left unpaired in the previous run (‘floating’ pre-synapses); that is, bassoon occurrences *j* that did not have *M*_*ij*_ = 1 after the first pass. For these, we found the postsynaptic entry *i* with the lowest distance and set the corresponding *M*_*ij*_ to 1 if this distance was below the matching limit *d*_matching_. This identified the previously unpaired pre-synapses that had SHANK2 signal in close spatial proximity; that is, within the matching limit.

First associating the closest pre- and postsynaptic sites in this way did not allow additional connections between pre- and postsynaptic detections that were already assembled into a synaptic connection in a more closely matching pair, which would occur if we simply matched all pre- and postsynaptic occurrences within the maximum matching distance *d*_matching_.

### Validation of synapse assembly

We classified automated detections of synaptic connections (that is, a connection between an individual presynaptic annotation and an individual postsynaptic annotation) as true positive if the following criteria were fulfilled: (i) true-positive detection of a bassoon occurrence; (ii) true-positive detection of a SHANK2 occurrence; and (iii) an automatically detected connection between these two that was also present in the manually annotated connections. We classified automated detections of synaptic connections as false positive if (i) a presynaptic and a postsynaptic detection were automatically connected but were not connected in the manual annotation or (ii) a false-positive molecule detection (bassoon or SHANK2 prediction) was assigned a synaptic connection. We classified detections of synaptic connections as false negative if a manually annotated synaptic connection was not present in the automated detection of synaptic connections. A typical scenario for this was a missed automated detection of pre- or postsynaptic sites, which can also happen if two neighbouring bassoon occurrences (or SHANK2 occurrences) are blurred into each other during the smoothing step and thus undersegmented.

For determining F1 scores, we revised false-negative detections that were close to the border of the analysed imaging volume in the *z* direction, because potential true-positive detections near the border might have been removed during the *z*-size filtering step. For this, we automatically generated two lists of detections: one that included objects spanning fewer than the specified number of planes in the *z* direction and a second in which these structures were removed. Further true-positive detections were then defined according to the following rules: (i) the detection was true positive in the first list and false negative in the second list and (ii) the detection was located within the first ten or last ten imaging planes of the volume.

### Automated post-processing of synapse detections

To account for those cases in which a particular synapse was not retained during one of the processing steps for automated synapse detection, we implemented an automated post-processing step for the respective test datasets (Supplementary Fig. 19). Such cases included failed detection in cases in which the synaptic immunolabelling was either weak or absent at a particular synapse and therefore did not meet the cut-off threshold (for example, owing to lack of SHANK2 expression), or the sampling of the structural LICONN feature did not pass the global threshold, or size filtering. Here, we identified unpaired pre- and postsynaptic detections, defined a local search area around those and checked whether bright structural features were present in the LICONN channel around those unpaired detections. For cases in which a prominent PSD or bright presynaptic feature was present, we classified them as additional pre- or postsynaptic detections, and added them to the respective lists of pre- and post-synapses, including them in the presynaptic–postsynaptic matching algorithm.

In brief, we checked individual rows and columns of the matching matrix *M*_*ij*_ that only contained zeros, corresponding to unmatched post- and pre-synapses, respectively. We then defined a 100 × 100 × 100-voxel^3^ bounding box around those locations (or smaller at imaging volume edges). We then applied intensity rescaling to the structural LICONN channel between p1 and p99.95, where p1 refers to the first percentile and p99.95 to the 99.95th percentile. We then removed background using Gaussian filters of two different widths at *σ* = 2 voxels and *σ* = 10 voxels, subtraction and clipping of negative values to zero. We then performed thresholding at thr = p95 + 0.4 × (p100 − p95), with p95 and p100 being the 95th and 100th percentile, respectively. For the dataset in Supplementary Fig. 19g–i, a factor of 0.5 instead of 0.4 was used. We then removed previously detected pre- and post-synapses by multiplying the resulting mask with a mask corresponding to an inverted semantic segmentation of the previously detected pre- and post-synapses. We then did a binary opening (eroding and then dilating) with a ball of two-voxel radius. We then performed instance segmentation with skimage.measure.label, removed structures smaller than 20 voxels and determined the weighted centroid on the intensity distribution in the underlying structural LICONN channel. We classified the resulting objects as a new pre- or postsynaptic detection when their Euclidian distance from the originally unpaired pre- or postsynaptic site was smaller than the matching limit *r*_matching_. We used these to update the list of pre- and postsynaptic detections used for the association to full synapses.

### Deep-learning-based synapse detection

We devised a strategy for identifying the locations of pre-synapses and post-synapses from the structural imaging channel alone. We used deep-learning-based prediction of synapse location with an approach that did not require human input for the generation of training data. For this, we implemented a U-net architecture for image translation to predict the locations of synaptic molecules from structural data, using measured molecule locations as input to the training. We then converted the predicted molecule locations to automatically generated synapse detections, for this step using a pipeline that was similar to the immunolabelling-based pipeline above. We generated two independent models for predicting the location of the presynaptic marker bassoon and for predicting the excitatory-synapse-specific postsynaptic marker SHANK2.

#### Generation of training data

We converted 3D super-resolved measurements of synaptic-molecule locations (based on specific immunolabelling), paired with the structural imaging channel, to training data with the following workflow. We first converted TIFF images to 32-bit floating format and split them into individual datasets for the structural and each of the immunolabelling channels, maintaining the original voxel size. We then implemented a preprocessing pipeline to automatically remove unspecific immunolabelling signals, similar to the approach taken for the immunolabelling-based synapse detection described above. However, unlike in that pipeline (in which we performed a strong smoothing step to avoid oversegmentation of the spatially structured bassoon and SHANK2 distributions), we here preserved the spatial distribution of the bassoon and SHANK2 signals more closely, allowing the deep-learning network to recapitulate the molecule arrangement in the respective synaptic compartments, including the lattice-like arrangement of bassoon, paralleled by high-intensity (protein-rich) structural features.

We rescaled intensity using the skimage.exposure.rescale_intensity function, setting the minimum to 101 and the maximum to the 99.995th percentile. We then removed background by applying isotropic Gaussian filters of two different widths using scipy.ndimage.gaussian_filter, with *σ* = 0.5 voxels and *σ* = 10 voxels, corresponding to signal and background, respectively, subtracting background from signal and clipping negative values to 0. We applied Otsu thresholding to generate a binary mask and used the skimage.measure.label function to derive an instance segmentation. We then sampled the maximum intensity value of the underlying structural data using the skimage.measure.regionprops function. We applied Otsu thresholding to distinguish segments with underlying high-intensity LICONN features, which we classified as specific immunolabellings, and without high-intensity features, which we classified as non-specific immunolabellings. We next removed the latter and converted the remaining segments back to an overall binary mask. To approximate the intensity distribution of the immunolabellings, we blurred the resulting mask with a 3D Gaussian of *σ* = 1 voxel.

For the structural channel, we applied simple intensity normalization to image pixel values im (im’ = (im − min)/(max − min)), where min is the 0th percentile and max is the 100th percentile of the intensity distribution. We converted to 8-bit format by multiplying the normalized data by 255 and clipping the intensity range between 0 and 255, followed by type casting.

We saved the paired immunolabelling and structural data in zarr format, allowing resource-efficient handling of large datasets.

#### Network architecture and training

We implemented deep-learning pipelines with Pytorch v.1.12.1 (https://pytorch.org) and used the Gunpowder framework v.1.2.2 (https://github.com/funkelab/gunpowder) to implement our data loading, augmentation, training and prediction pipeline that conveniently allowed processing of big datasets. For predicting molecule location, we used the 3D U-net architecture from a previous study^46^. We used Adam’s optimizer (torch.optim.Adam) with a 10^−4^ learning rate. We used the torch.nn.MSEloss function to implement mean squared error as a loss function. The batch size was set to 8 and the training input size was 64 × 128 × 128 voxels, with the smallest number referring to the *z* direction.

We performed network training on ISTA’s high-performance computing cluster, using SLURM for task allocation. We typically requested 8 CPUs, graphics acceleration by 1 GPU (NVIDIA GeForce) and 700 GB of RAM.

We trained on datasets with a 300-nm *z*-step size (6 individual tiles, total volume of 16,699 µm^3^, containing around 16,250 excitatory synapses) and applied the resulting model to datasets with a 200-nm, 300-nm or 400-nm *z*-step size. We performed training with 100,000 iterations and saved intermediate check-points every 5,000 iterations. For selecting a model for the different *z*-step sizes, we applied models from various check-points and took the best-performing check-point when evaluating F1 on the respective test datasets. For the 200-nm *z*-step size, we took the 70,000 and 50,000 iteration check-points for bassoon and SHANK2 prediction, respectively, and we applied the same model to the 400-nm *z*-step size. For the 300-nm *z*-step size, we used the 20,000 iterations check-point for both bassoon and SHANK2 prediction.

During the validation phase for the 300-nm *z*-step size prediction, training data comprised 5 of the 6 tiles (total volume of 14,207 µm^3^, with around 13,650 excitatory synapses), and one dataset was used for testing. For final training, all six datasets were included. During training, patches of training data (normalized between 0 and 1 with gp.normalize) were randomly sampled from the training datasets with equal probability and used in training if at least 0.1% of voxels were included in the binary mask generated during preprocessing of molecular signals (that is, 1,048.6 voxels of the 64 × 128 × 128-voxel patches). Training data were augmented by mirroring and transposing in the *xy* direction on structural and molecular channels. In addition, we applied intensity augmentation on the structural channel with (0.9, 1.1, −0.1, 0.1) for the two scaling and the two intensity shifting parameters, respectively, and enabled range clipping.

#### Prediction

Prediction was performed on ISTA’s high-performance computing cluster using SLURM for task allocation. We requested 8 CPUs, graphics acceleration by 1 GPU (NVIDIA GeForce RTX 2080 Ti or similar) and 100 GB of RAM. Prediction was performed in a piece-wise, ‘scanning’ mode, again with a patch size of 64 × 128 × 128 voxels after converting LICONN structural data to zarr format while applying the same normalization procedure as for the training data.

To convert predicted molecule locations to point annotations, we first rescaled intensity using the skimage.exposure.rescale_intensity, taking p1 and p99.95 as limits, with p1 referring to the first percentile and p99.95 to the 99.95th percentile. We then applied 3D Gaussian filtering with *σ* = 5 for bassoon and SHANK2. We applied Otsu thresholding to derive a binary mask and skimage.measure.label to extract instance segmentations. We erased volume segments smaller than 60 voxels and those that covered fewer than 13 imaging planes. We applied the skimage.measure.regionprops function to determine the weighted centroid for each segment on the basis of the intensity of the underlying LICONN structural channel, and placed a point annotation at that location.

Association of pre- and postsynaptic sites and validation of deep-learning-based synapse prediction were performed with the same pipeline as was used for immunolabelling-based synapse detection. The automated post-processing step for synapse detection was identical to the corresponding step in the immunolabelling-based synapse-detection pipeline and was applied to the test datasets (Fig. 4l–n and Supplementary Fig. 19).

### Visualization

Neurite and synaptic segmentations, skeletons and imaging volumes were visualized in 3D with Blender versions 2.92, 3.2 or 3.51 (https://www.blender.org/). Video files were also generated with Blender. For visualizing neurites on an overview scale (Figs. 1a, 2c,d), precomputed meshes from Neuroglancer were used. Detailed views of 3D renderings (Fig. 1a zoomed dendrite, Figs. 1e,f, 3c) were based on meshes generated with the marching cubes implementation in scikit-image. For 3D rendering of molecular signals in the context of neuronal segmentations, immunolabelling channels and predicted molecule distributions were intensity scaled according to visual inspection, background was removed with *σ* ≈ 0.5 and *σ* ≈ 5 to preserve shapes and then Otsu thresholding was applied. To associate these signals with a particular neuronal segment, the segment was expanded with a ball of radius 6 and any molecular signals with at least 1 voxel overlap were retained. Meshes were again generated with the marching cubes algorithm.

### Associating synapse detections with FFN-derived neuronal segments

To map detected synapses onto selected neuronal segments, we first expanded the segment with a ball of radius of 5 or 6 voxels. To map postsynaptic sites onto dendrites, we selected those detections whose centre coordinates were located within the expanded neuronal segment. We recalled the corresponding presynaptic partners using the matching matrix *M*_*ij*_ to generate the list of full synapses associated with that segment. For axons, we first identified the pre-synapses located within the expanded segment, and then retrieved the corresponding post-synapses from the matching matrix. Owing to the large size of the imaging volume, computations were performed on manually defined patches, and results were combined for visualization and further analysis.

### Automated analysis of connectivity based on deep-learning-based synapse prediction and neurite segmentation

To analyse connectivity in Fig. 4p, we first performed deep-learning-based synapse prediction on the imaging volume. For this, we predicted bassoon and SHANK2 molecule locations in smaller overlapping patches of the volume, and combined them into the final full-size prediction volume. Next, we converted the predicted molecule locations into synaptic point annotations using the algorithm described above. Because of the large size of the volume, pre-synapse and post-synapse annotations were computed on manually defined non-overlapping patches. Association of pre- and post-synapses—that is, computation of the matching matrix *M*_*ij*_—was performed on the entire volume to avoid errors in full synapse assembly owing to separation by a patch boundary. We then associated pre- and postsynaptic annotations with the neuronal segmentation.

For each pre-synapse (or post-synapse) location, we identified the underlying segment ID from the FFN-based neurite segmentation. If no segment was present at the coordinates of a specific pre- or postsynaptic detection, we performed an automated search in a single plane for the nearest non-zero segment in a 20 × 20-voxel neighbourhood. If (i) the squared distance between the respective pre-synapse (post-synapse) and the nearest non-zero segment was less than 30 voxel^2^ and (ii) the segment was classified as an axon (dendrite) by the neurite classifier described above, the respective pre-synapse (post-synapse) was mapped to the segment.

Finally, we combined the information from the matching matrix *M*_*ij*_ and the synapse–segment associations obtained in the previous step to compute a matrix of connectivity between neuronal segments. For this, we iterated through the matching matrix *M*_*ij*_ and added a connection between the segment associated with post-synapse *i* and the segment associated with pre-synapse *j*.

### Statistics and reproducibility

GraphPad Prism v.10.1.2 was used for statistical analysis and plotting. Bootstrap analysis in Supplementary Fig. 21 was performed as described previously^68^, based on code available at https://gitlab.mpcdf.mpg.de/connectomics/human_primate. The violin plot in Fig. 2g was generated with seaborn.violin.plot (https://seaborn.pydata.org/generated/seaborn.violinplot.html). This is a proof-of-concept study focusing on the development of new technology and its applicability. Accordingly, no prior determination of statistical sample size was performed. Once the technology was established and the data quality was to our satisfaction, experiments were performed in multiple replicates to ensure and demonstrate reproducibility. For the comparison of primary cilia length in wild-type versus *Hnrnpu*^*+/−*^ mice (Fig. 5g), we chose *n* = 3 biological replicates in each group, recorded across a total of *n* = 7 technical replicates to demonstrate that a biologically meaningful number of replicates can be analysed with LICONN. No randomization between experimental groups was performed. The comparison of cilia length between wild-type and mutant mice (Fig. 5g) was performed with an unpaired two-tailed *t*-test (*P* = 0.64; not significant). Measurements of cellular parameters (for example, distance measurements within synapses, periodicity and connectivity) were performed to show that quantitative information that is consistent with previous measurements can be extracted from LICONN data. Researchers were aware of the source of the data and the purpose of the study. Quantification of segmentation accuracy and synapse-detection fidelity were performed to evaluate how well computational algorithms could reproduce human ground-truth annotations. In these cases, blinding was not possible or appropriate. For determining the accuracy of human tracings relative to independent ground-truth information from sparse eGFP expression, blinding to that independent ground-truth information was performed. Human tracers were blinded to the eGFP channel for the analysis in Fig. 1e,f and Supplementary Figs. 13, 14. Blinding was implemented through the permissions settings in webKnossos.

#### Replication

To confirm the reproducibility of the technology, we performed a series of technical replicates, which were typically recorded across several biological specimens, as indicated below. For analyses, only datasets of high labelling and imaging quality were pursued. Typically, during the development phase, a number of experiments with some variation of parameters were performed. In all images, representative data from single experiments are shown. For some of the experiments that we performed routinely, the stated numbers give lower bounds and we did not count further replicates beyond *n* = 10. Fig. 1a,b: imaging data representative of acquisition of expansion and multi-tile imaging in *n* = 10 replicates across *n* = 3 mice. Fig. 1b represents subregion of dataset from Fig. 1a. Fig. 1c: 192 synapses analysed with multiple line profiles per synapse, in *n* = 3 technical replicates across *n* = 2 mice. Fig. 1d: 32 distance measurements across *n* = 2 mice. Fig. 1e and Supplementary Figs. 13, 14: 37 axon stretches analysed in 12 datasets from *n* = 3 technical replicates across *n* = 3 mice. Fig. 1f, Supplementary Fig. 15: 5 datasets from *n* = 3 technical replicates across *n* = 2 mice. Fig. 1g and Supplementary Fig. 6: single-tile LICONN measurements were performed with a large number of technical and biological replicates (*n* ≫ 20). Manual neurite annotation was performed in *n* = 2 datasets across *n* = 2 mice. Fig. 2a–i and Supplementary Fig. 16: multi-tile LICONN measurements were performed in *n* = 10 replicates. FFN-based segmentation was applied to *n* = 6 datasets. Comprehensive proofreading of all neuronal structures (Fig. 2a–g) and quantification of segmentation results using manually generated reference skeletons (Fig. 2h and Supplementary Figs. 16, 17) were performed in *n* = 1 dataset. Fig. 3a: immunolabelling for bassoon and PSD95 was performed in *n* = 3 technical replicates across *n* = 2 mice. Fig. 3b: analysis of 106 synapses in *n* = 3 technical replicates across *n* = 2 mice. Fig. 3c: visualization from the dataset in Fig. 3a. Bassoon and SHANK2 labelling was performed in *n* = 5 replicates across 3 mice. Fig. 3d: synaptic immunolabellings were replicated *n* = 2 times, with further immunolabellings for synaptic targets in various contexts. Fig. 3e: 185 synapses analysed in 3 imaging volumes from *n* = 1 technical replicate. Fig. 3f–h and Supplementary Fig. 19: immunolabelling-based, automated synapse-detection accuracy was evaluated against manually generated ground truth in *n* = 3 technical replicates recorded across *n* = 2 mice. Fig. 3f represents a schematic illustration of synapse detection (synapse annotations placed manually, *n* = 1). Fig. 3i: visualization of synapse detections was performed for *n* = 1 dendrite. Fig. 3j,k: immunolabelling for inhibitory synapses was performed in *n* = 5 technical replicates. Fig. 4a–k and Supplementary Figs. 20, 21: manual connectivity analysis was performed across 2 imaging volumes (total 154,560 µm^3^) in *n* = 1 technical replicate (*n* = 1 mouse). Fig. 4b–f: quantification of synaptic contacts of spiny dendrites was performed on *n* = 11 dendrites. Fig. 4h,i: output analysis and spine tracing was performed for *n* = 19 axons. Fig. 4k: output was analysed for *n* = 8 AIS-seeded axons. Fig. 4l–m and Supplementary Fig. 19: accuracy of deep-learning-based synapse prediction was evaluated against manually generated ground-truth annotations in *n* = 3 technical replicates recorded across *n* = 2 mice. Fig. 4n: mapping of synaptic inputs and outputs was performed in *n* = 1 cell and represents further analysis of the dataset in Fig. 1a. Fig. 4o: analysis of data in Fig. 2a (*n* = 1 dataset). Fig. 4p: visualization of excitatory and inhibitory inputs was done for *n* = 1 dendrite. Fig. 5a: immunolabellings were replicated *n* = 2 times in *n* = 2 mice. Fig. 5b: immunolabellings were replicated *n* = 3 times. Fig. 5c: imaging data representative of *n* = 10 replicates across *n* = 3 mice. Fig. 5e: immunolabellings were replicated *n* = 2 times. Fig. 5f: analysis of 78 cells from dataset in Fig. 1a. Fig. 5g: *Hnrnpu*^+/*−*^-haploinsufficient mice: 80 cells from *n* = 7 technical replicates across *n* = 3 mice; wild type: 78 cells from *n* = 7 technical replicates across *n* = 3 mice. Fig. 5h and Extended Data Fig. 9: representative of *n* = 2 replicates in *n* = 2 mice. Fig. 5i: Cilia diameter: 52 cross-sections; microtubule doublet distance: 54 distance measurements, analysed across *n* = 2 technical replicates in *n* = 1 mouse (5 imaging volumes). Fig. 5j and Extended Data Fig. 5: immunolabellings were reproduced in *n* = 2 replicates. Fig. 5k: representative of *n* = 3 replicates. Fig. 5l: representative of immunolabelling in *n* = 3 replicates. Fig. 5m: gap-junction and inhibitory-synapse density evaluated across *n* = 4 imaging volumes in cortex and hippocampus from *n* = 1 mouse. Extended Data Fig. 2: high-resolution large-scale tiling in hippocampus was performed in *n* = 1 replicate in *n* = 1 mouse. Extended Data Fig. 3: immunolabelling for RIM1/2 and VGLUT1 is representative of *n* = 2 technical replicates and bassoon and PSD95 immunolabelling of *n* = 3 replicates across *n* = 2 mice. Extended Data Fig. 5: the displayed combinations of immunolabellings for MBP and Cnx-43 were performed in *n* = 1 technical replicate, RIM1/2 and VGLUT1 in *n* = 2 technical replicates, GFAP and Cnx-43 in *n* = 2 technical replicates and vimentin and PMP70 in *n* = 1 replicate. Extended Data Fig. 6: LICONN imaging in different brain regions was performed in *n* = 1 mouse. Extended Data Fig. 7: overview imaging in CA3 stratum lucidum was performed in *n* = 2 technical replicates, single tile measurements were performed in multiple technical replicates. Extended Data Fig. 8: imaging of the various layers was performed in *n* = 1 mouse. Extended Data Fig. 9: data representative of replicates in *n* = 2 mice. Extended Data Fig. 10a: imaging data representative of LICONN in 300-µm-thick slices in *n* = 3 mice. Extended Data Fig. 10c–f: post-expansion hydrogel sectioning was performed in *n* = 3 technical replicates. Extended Data Fig. 10h: block-face imaging and sectioning over 12 rounds was performed in *n* = 1 specimen. Supplementary Fig. 1: data points correspond to individual technical replicates. Supplementary Fig. 1a: *n* = 1 replicate for 0.25%, 0.15%, 0.005%, *n* = 2 replicates otherwise. Supplementary Fig. 1b: different hydrogel recipes were tested in the following technical replicates: replicates 1–4 and 15: *n* = 2; replicates 5, 9, 11 and 13: *n* = 3; replicates 6–8, 10, 12 and 14: *n* = 4. Supplementary Fig. 1c: experiments were performed in the following technical replicates: 0.05% APS and TEMED: *n* = 3 replicates. 0.15% APS and TEMED: replicates 7, 8, 10, 11 and 14: *n* = 3 replicates; replicate 13: *n* = 2 replicates. Supplementary Fig. 1d: *n* = 2 technical replicates. Supplementary Fig. 1e: technical replicates: first hydrogel: replicates 11, 7, 14, 8 and 10: *n* = 3; replicate 13: *n* = 2. Final hydrogel: *n* = 4. Supplementary Fig. 1f: a single hydrogel was subdivided after the first gelation step. Expansion factors for the second expandable hydrogel were determined in the following technical replicates: replicate 11: *n* = 3; replicates 13, 7, 14 and 1: *n* = 4. Supplementary Fig. 1g: *n* = 4 technical replicates. Supplementary Fig. 2: data representative of *n* = 3 technical replicates. Supplementary Fig. 3: each condition was technically replicated *n* = 2 times. Hydrogels 14 and 15 were replicated *n* = 5 times. Supplementary Fig. 4a–c: individual data points represent individual mice. Number of mice analysed: Supplementary Fig. 4a–c: 20% AA: *n* = 3; 0% AA, 5% AA, 10% AA, 12.5% AA: *n* = 4; 15% AA: *n* = 6. Supplementary Fig. 4f: 0% AA: *n* = 15 cells across *n* = 1 technical replicate; 5% AA: *n* = 32 cells across *n* = 3 technical replicates; 10% AA: *n* = 98 cells across *n* = 3 technical replicates; 12.5% AA: *n* = 49 cells across *n* = 2 technical replicates; 15% AA: *n* = 74 cells across *n* = 3 technical replicates; 20% AA: *n* = 20 cells across *n* = 2 technical replicates; 25% AA: *n* = 20 cells across *n* = 2 technical replicates; 30% AA: *n* = 17 cells across *n* = 2 technical replicates. Supplementary Fig. 5: data representative of *n* = 3 technical replicates. Supplementary Fig. 6: manual segmentation in datasets with NAS anchoring was performed in *n* = 1 replicate. Supplementary Fig. 7: data representative of *n* = 3 technical replicates. Supplementary Fig. 8: data representative of *n* = 2 technical replicates. Supplementary Fig. 9: data representative of *n* = 3 technical replicates for each condition. Supplementary Fig. 10a: Measurement error represents mean ± s.d. at given measurement length from sampling 2 × 10^5^ pairs of points in pre- and post-expansion images (see Methods) from the specific single measurement (*n* = 1 imaging volume) shown in the figure panel. Data representative of *n* = 4 technical replicates from *n* = 3 mice. Supplementary Fig. 10b,c: RMS measurement error over different measurement lengths for the first and second expansion steps and for the overall LICONN procedure were evaluated as mean ± s.d. across *n* = 4 technical replicates (using a total of 14 imaging volumes), recorded in cortex across *n* = 3 mice. Supplementary Fig. 10d: 21 imaging volumes analysed across *n* = 6 technical replicates from *n* = 3 mice. Supplementary Fig. 11: overview image in region corresponding to dataset in Fig. 1a (*n* = 1 dataset). Supplementary Fig. 12a,b: data representative of application of CLAHE in *n* = 10 technical replicates. Illustration of visual appearance of CLAHE-processed data at different slopes (Supplementary Fig. 12b) was performed in *n* = 1 dataset. Supplementary Fig. 18: data representative of *n* = 10 technical replicates. Visual illustration of resolution improvement by iterative expansion as shown in the figure was performed in *n* = 1 specimen. Supplementary Figs. 20, 21: manual tracings were performed across 2 imaging volumes from *n* = 1 specimen (*n* = 1 technical replicate) imaged in the high-resolution overview scan. Supplementary Fig. 22: tracing with deep-learning prediction of synapses was performed in *n* = 1 technical replicate. Supplementary Figs. 23, 24: immunolabellings were technically replicated *n* = 2 times. Supplementary Fig. 25: high-resolution overview imaging of the region analysed in Fig. 5g was performed in *n* = 1 specimen.

### Creation of figures

Figures were created in Adobe Illustrator v.27.5 and v.27.7. The schematic in Fig. 5d and brain cross-section schematics in Supplementary Fig. 4 were created in BioRender (2025, https://BioRender.com/j05y134 and https://BioRender.com/x93f652).

### Materials availability

*Hnrnpu*^*+/−*^ mice are available from the authors upon request.

### Reporting summary

Further information on research design is available in the Nature Portfolio Reporting Summary linked to this article.

## Online content

Any methods, additional references, Nature Portfolio reporting summaries, source data, extended data, supplementary information, acknowledgements, peer review information; details of author contributions and competing interests; and statements of data and code availability are available at 10.1038/s41586-025-08985-1.

## Supplementary information

Supplementary Information PDF file containing Supplementary Tables 1–8 and Supplementary Figs. 1–25, providing further information on LICONN optimization, analysis and application.

Reporting Summary

Source Data for Supplementary Figures.

Supplementary Video 1 **Manual segmentation of LICONN volume**. Fly-through of the original data in Fig. 1g, with step-by-step appearance of manually annotated segments.

Supplementary Video 2 **Automated segmentation**. Rendering of original data in Fig. 2a (hippocampus) with a subset of neurite segments from the FFN segmentation, with comprehensive proofreading of neuronal structures.

Supplementary Video 3 **Excitatory synaptic inputs and outputs of a cortical neuron based on deep-learning prediction of synapses**. Rotation of rendering in Fig. 4n.

Supplementary Video 4 **Multi-round block-face imaging and sectioning**. Fly-through of the original data in Extended Data Fig. 10h, covering 205 µm along the axial direction after fusion of the imaging subvolumes arranged on a 3×3×12 grid. Voxels are downsampled in the *xy* direction owing to file-size limitations. Original data after CLAHE processing and fusion of the multi-round data are available in browsable format at: https://neuroglancer-demo.appspot.com/#!gs://liconn-public/ng_states/multiround_fusion.json.

## Source data

Source Data Fig. 1

Source Data Fig. 2

Source Data Fig. 3

Source Data Fig. 4

Source Data Fig. 5

## Data Availability

Original data and/or segmentations for example datasets are available in browsable format through Neuroglancer, as follows. Full segmentation with comprehensive proofreading of neuronal structures: Fig. 2a–f: https://neuroglancer-demo.appspot.com/#!gs://liconn-public/ng_states/expid82.json; Fig. 2c: https://neuroglancer-demo.appspot.com/#!gs://liconn-public/ng_states/expid82_fig2_axons.json; and Fig. 2d: https://neuroglancer-demo.appspot.com/#!gs://liconn-public/ng_states/expid82_fig2_dends.json. Additional datasets with lateral and/or axial fusion: Fig. 1a,b: https://neuroglancer-demo.appspot.com/#!gs://liconn-public/ng_states/fig1a.json; Extended Data Fig. 10c–f: https://neuroglancer-demo.appspot.com/#!gs://liconn-public/ng_states/expid146.json; and Extended Data Fig. 10h: https://neuroglancer-demo.appspot.com/#!gs://liconn-public/ng_states/multiround_fusion.json. Further data are available at the ISTA’s data repository: 10.15479/AT:ISTA:18697. Source data are provided with this paper.

## Code availability

Custom code for synapse detection, connectivity analysis and distortion analysis is available at https://github.com/danzllab/LICONN. FFNs are available at https://github.com/google/ffn, including a Jupyter notebook for performing FFN inference on LICONN data (https://github.com/google/ffn/blob/master/notebooks/jax_ffn_inference_liconn.ipynb, git hash: 12d680e). SOFIMA is available at https://github.com/google-research/sofima (git hash 64d5c7c).

## References

[1] Kasthuri, N. et al. Saturated reconstruction of a volume of neocortex. *Cell* **162**, 648–661 (2015). 26232230 10.1016/j.cell.2015.06.054

[2] Jefferis, G., Collinson, L., Bosch, C., Costa, M. & Schlegel, P. *Scaling up Connectomics: The Road to a Whole Mouse Brain Connectome*. https://wellcome.org/reports/scaling-connectomics (Wellcome Trust, 2023).

[3] Kornfeld, J. & Denk, W. Progress and remaining challenges in high-throughput volume electron microscopy. *Curr. Opin. Neurobiol.* **50**, 261–267 (2018). 31160001 10.1016/j.conb.2019.05.003

[4] White, J. G., Southgate, E., Thomson, J. N. & Brenner, S. The structure of the nervous system of the nematode *Caenorhabditis elegans*. *Phil. Trans. R. Soc. B* **314**, 1–340 (1986). 22462104 10.1098/rstb.1986.0056

[5] Scheffer, L. K. et al. A connectome and analysis of the adult *Drosophila* central brain. *eLife* **9**, e57443 (2020). 32880371 10.7554/eLife.57443 PMC7546738

[6] Winding, M. et al. The connectome of an insect brain. *Science* **379**, eadd9330 (2023). 36893230 10.1126/science.add9330 PMC7614541

[7] Dorkenwald, S. et al. Neuronal wiring diagram of an adult brain. *Nature* **634**, 124–138 (2024). 39358518 10.1038/s41586-024-07558-y PMC11446842

[8] The MICrONS Consortium. Functional connectomics spanning multiple areas of mouse visual cortex. *Nature*10.1038/s41586-025-08790-w (2025). 10.1038/s41586-025-08790-w PMC11981939 40205214

[9] Shapson-Coe, A. et al. A petavoxel fragment of human cerebral cortex reconstructed at nanoscale resolution. *Science* **384**, eadk4858 (2024). 38723085 10.1126/science.adk4858 PMC11718559

[10] Loomba, S. et al. Connectomic comparison of mouse and human cortex. *Science* **377**, eabo0924 (2022). 35737810 10.1126/science.abo0924

[11] Fang, T. et al. Nanobody immunostaining for correlated light and electron microscopy with preservation of ultrastructure. *Nat. Methods* **15**, 1029–1032 (2018). 30397326 10.1038/s41592-018-0177-x PMC6405223

[12] Fulton, K. A. & Briggman, K. L. Permeabilization-free en bloc immunohistochemistry for correlative microscopy. *eLife* **10**, e63392 (2021). 33983117 10.7554/eLife.63392 PMC8118656

[13] Han, X. et al. Multiplexed volumetric CLEM enabled by scFvs provides insights into the cytology of cerebellar cortex. *Nat. Commun.* **15**, 6648 (2024). 39103318 10.1038/s41467-024-50411-z PMC11300613

[14] Holler, S., Köstinger, G., Martin, K. A. C., Schuhknecht, G. F. P. & Stratford, K. J. Structure and function of a neocortical synapse. *Nature* **591**, 111–116 (2021). 33442056 10.1038/s41586-020-03134-2

[15] Sahl, S. J., Hell, S. W. & Jakobs, S. Fluorescence nanoscopy in cell biology. *Nat. Rev. Mol. Cell Biol.* **18**, 685–701 (2017). 28875992 10.1038/nrm.2017.71

[16] Chen, F., Tillberg, P. W. & Boyden, E. S. Expansion microscopy. *Science* **347**, 543–548 (2015). 25592419 10.1126/science.1260088 PMC4312537

[17] Shen, F. Y. et al. Light microscopy based approach for mapping connectivity with molecular specificity. *Nat. Commun.* **11**, 4632 (2020). 32934230 10.1038/s41467-020-18422-8 PMC7493953

[18] Klar, T. A., Jakobs, S., Dyba, M., Egner, A. & Hell, S. W. Fluorescence microscopy with diffraction resolution barrier broken by stimulated emission. *Proc. Natl Acad. Sci. USA* **97**, 8206–8210 (2000). 10899992 10.1073/pnas.97.15.8206 PMC26924

[19] Tønnesen, J., Inavalli, V. V. G. K. & Nägerl, U. V. Super-resolution imaging of the extracellular space in living brain tissue. *Cell* **172**, 1108–1121 (2018). 29474910 10.1016/j.cell.2018.02.007

[20] Velicky, P. et al. Dense 4D nanoscale reconstruction of living brain tissue. *Nat. Methods* **20**, 1256–1265 (2023). 37429995 10.1038/s41592-023-01936-6 PMC10406607

[21] Michalska, J. M. et al. Imaging brain tissue architecture across millimeter to nanometer scales. *Nat. Biotechnol.* **42**, 1051–1064 (2024). 37653226 10.1038/s41587-023-01911-8 PMC11252008

[22] Ku, T. et al. Multiplexed and scalable super-resolution imaging of three-dimensional protein localization in size-adjustable tissues. *Nat. Biotechnol.* **34**, 973–981 (2016). 27454740 10.1038/nbt.3641 PMC5070610

[23] Truckenbrodt, S. et al. X10 expansion microscopy enables 25 nm resolution on conventional microscopes. *EMBO Rep.* **19**, e45836 (2018). 29987134 10.15252/embr.201845836 PMC6123658

[24] Truckenbrodt, S., Sommer, C., Rizzoli, S. O. & Danzl, J. G. A practical guide to optimization in X10 expansion microscopy. *Nat. Protoc.* **14**, 832–863 (2019). 30778205 10.1038/s41596-018-0117-3

[25] Damstra, H. G. et al. Visualizing cellular and tissue ultrastructure using ten-fold robust expansion microscopy (TREx). *eLife* **11**, e73775 (2022). 35179128 10.7554/eLife.73775 PMC8887890

[26] Klimas, A. et al. Magnify is a universal molecular anchoring strategy for expansion microscopy. *Nat. Biotechnol.* **41**, 858–869 (2023). 36593399 10.1038/s41587-022-01546-1 PMC10264239

[27] Park, H. E. et al. Scalable and isotropic expansion of tissues with simply tunable expansion ratio. *Adv. Sci.* **6**, 1901673 (2019). 10.1002/advs.201901673 PMC6864509 31763149

[28] Chang, J.-B. et al. Iterative expansion microscopy. *Nat. Methods* **14**, 593–599 (2017). 28417997 10.1038/nmeth.4261 PMC5560071

[29] Sarkar, D. et al. Revealing nanostructures in brain tissue via protein decrowding by iterative expansion microscopy. *Nat. Biomed. Eng.* **6**, 1057–1073 (2022). 36038771 10.1038/s41551-022-00912-3 PMC9551354

[30] M’Saad, O. & Bewersdorf, J. Light microscopy of proteins in their ultrastructural context. *Nat. Commun.* **11**, 3850 (2020). 32737322 10.1038/s41467-020-17523-8 PMC7395138

[31] Mao, C. et al. Feature-rich covalent stains for super-resolution and cleared tissue fluorescence microscopy. *Sci. Adv.* **6**, eaba4542 (2020). 32518827 10.1126/sciadv.aba4542 PMC7253160

[32] M’Saad, O. et al. All-optical visualization of specific molecules in the ultrastructural context of brain tissue. Preprint at *bioRxiv*10.1101/2022.04.04.486901 (2022). 10.1038/s41587-025-02905-4 PMC13338931 41299044

[33] Korogod, N., Petersen, C. C. & Knott, G. W. Ultrastructural analysis of adult mouse neocortex comparing aldehyde perfusion with cryo fixation. *eLife* **4**, e05793 (2015). 26259873 10.7554/eLife.05793 PMC4530226

[34] Chung, K. et al. Structural and molecular interrogation of intact biological systems. *Nature* **497**, 332–337 (2013). 23575631 10.1038/nature12107 PMC4092167

[35] Pallotto, M., Watkins, P. V., Fubara, B., Singer, J. H. & Briggman, K. L. Extracellular space preservation aids the connectomic analysis of neural circuits. *eLife* **4**, e08206 (2015). 26650352 10.7554/eLife.08206 PMC4764589

[36] Park, Y.-G. et al. Protection of tissue physicochemical properties using polyfunctional crosslinkers. *Nat. Biotechnol.* **37**, 73–83 (2018). 10.1038/nbt.4281 PMC6579717 30556815

[37] Cui, Y. et al. Expansion microscopy using a single anchor molecule for high-yield multiplexed imaging of proteins and RNAs. *PLoS One* **18**, e0291506 (2023). 37729182 10.1371/journal.pone.0291506 PMC10511132

[38] Tillberg, P. W. et al. Protein-retention expansion microscopy of cells and tissues labeled using standard fluorescent proteins and antibodies. *Nat. Biotechnol.* **34**, 987–992 (2016). 27376584 10.1038/nbt.3625 PMC5068827

[39] Chozinski, T. J. et al. Expansion microscopy with conventional antibodies and fluorescent proteins. *Nat. Methods* **13**, 485–488 (2016). 27064647 10.1038/nmeth.3833 PMC4929147

[40] Januszewski, M., Blakely, T. & Lueckmann, J.-M. SOFIMA: Scalable optical flow-based image montaging and alignment. *Zenodo*10.5281/zenodo.10534541 (2024).

[41] Xu, K., Zhong, G. & Zhuang, X. Actin, spectrin, and associated proteins form a periodic cytoskeletal structure in axons. *Science* **339**, 452–456 (2013). 23239625 10.1126/science.1232251 PMC3815867

[42] Januszewski, M. et al. High-precision automated reconstruction of neurons with flood-filling networks. *Nat. Methods* **15**, 605–610 (2018). 30013046 10.1038/s41592-018-0049-4

[43] Mishchenko, Y. et al. Ultrastructural analysis of hippocampal neuropil from the connectomics perspective. *Neuron* **67**, 1009–1020 (2010). 20869597 10.1016/j.neuron.2010.08.014 PMC3215280

[44] Bartol, T. M. Jr et al. Nanoconnectomic upper bound on the variability of synaptic plasticity. *eLife* **4**, e10778 (2015). 26618907 10.7554/eLife.10778 PMC4737657

[45] Dani, A., Huang, B., Bergan, J., Dulac, C. & Zhuang, X. Superresolution imaging of chemical synapses in the brain. *Neuron* **68**, 843–856 (2010). 21144999 10.1016/j.neuron.2010.11.021 PMC3057101

[46] Ounkomol, C., Seshamani, S., Maleckar, M. M., Collman, F. & Johnson, G. R. Label-free prediction of three-dimensional fluorescence images from transmitted-light microscopy. *Nat. Methods* **15**, 917–920 (2018). 30224672 10.1038/s41592-018-0111-2 PMC6212323

[47] Motta, A. et al. Dense connectomic reconstruction in layer 4 of the somatosensory cortex. *Science* **366**, eaay3134 (2019). 31649140 10.1126/science.aay3134

[48] Elabbady, L. et al. Perisomatic ultrastructure efficiently classifies cells in mouse cortex. *Nature* **640**, 478–486 (2025). 10.1038/s41586-024-07765-7 PMC11981918 40205216

[49] Sheu, S.-H. et al. A serotonergic axon–cilium synapse drives nuclear signaling to alter chromatin accessibility. *Cell* **185**, 3390–3407 (2022). 36055200 10.1016/j.cell.2022.07.026 PMC9789380

[50] Ott, C. M. et al. Ultrastructural differences impact cilia shape and external exposure across cell classes in the visual cortex. *Curr. Biol.* **34**, 2418–2433 (2024). 38749425 10.1016/j.cub.2024.04.043 PMC11217952

[51] Sapir, T. et al. Heterogeneous nuclear ribonucleoprotein U (HNRNPU) safeguards the developing mouse cortex. *Nat. Commun.* **13**, 4209 (2022). 35864088 10.1038/s41467-022-31752-z PMC9304408

[52] Mastropasqua, F. et al. Deficiency of the heterogeneous nuclear ribonucleoprotein U locus leads to delayed hindbrain neurogenesis. *Biol. Open* **12**, bio060113 (2023). 37815090 10.1242/bio.060113 PMC10581386

[53] Anderson, J. R. et al. Exploring the retinal connectome. *Mol. Vis.* **17**, 355–379 (2011). 21311605 PMC3036568

[54] Lin, J. H.-C. et al. Gap-junction-mediated propagation and amplification of cell injury. *Nat. Neurosci.* **1**, 494–500 (1998). 10196547 10.1038/2210

[55] Abbott, L. F. et al. The mind of a mouse. *Cell* **182**, 1372–1376 (2020). 32946777 10.1016/j.cell.2020.08.010

[56] Alon, S. et al. Expansion sequencing: spatially precise in situ transcriptomics in intact biological systems. *Science* **371**, eaax2656 (2021). 33509999 10.1126/science.aax2656 PMC7900882

[57] Wang, X. et al. Three-dimensional intact-tissue sequencing of single-cell transcriptional states. *Science* **361**, eaat5691 (2018). 29930089 10.1126/science.aat5691 PMC6339868

[58] Eng, C.-H. L. et al. Transcriptome-scale super-resolved imaging in tissues by RNA seqFISH+. *Nature* **568**, 235–239 (2019). 30911168 10.1038/s41586-019-1049-y PMC6544023

[59] Wang, G., Moffitt, J. R. & Zhuang, X. Multiplexed imaging of high-density libraries of RNAs with MERFISH and expansion microscopy. *Sci. Rep.* **8**, 4847 (2018). 29555914 10.1038/s41598-018-22297-7 PMC5859009

[60] Lee, B. R. et al. Scaled, high fidelity electrophysiological, morphological, and transcriptomic cell characterization. *eLife* **10**, e65482 (2021). 34387544 10.7554/eLife.65482 PMC8428855

[61] Danzl, J. et al. Research data for publication ‘Light-microscopy-based connectomic reconstruction of mammalian brain tissue’. Institute of Science and Technology Austria 10.15479/AT:ISTA:18697 (2025).

[62] Sun, D. et al. Click-ExM enables expansion microscopy for all biomolecules. *Nat. Methods* **18**, 107–113 (2021). 33288959 10.1038/s41592-020-01005-2

[63] Bogovic, J. A., Hanslovsky, P., Wong, A. & Saalfeld, S. Robust registration of calcium images by learned contrast synthesis. In *2016 IEEE 13th International Symposium on Biomedical Imaging (ISBI)* 1123–1126 (IEEE, 2016).

[64] Thirion, J.-P. Image matching as a diffusion process: an analogy with Maxwell’s demons. *Med. Image Anal.* **2**, 243–260 (1998). 9873902 10.1016/s1361-8415(98)80022-4

[65] Loshchilov, I. & Hutter, F. Decoupled weight decay regularization. In *International Conference on Learning Representations (ICLR 2019)* https://openreview.net/forum?id=Bkg6RiCqY7 (OpenReview, 2019).

[66] Sato, M., Bitter, I., Bender, M. A., Kaufman, A. E. & Nakajima, M. TEASAR: tree-structure extraction algorithm for accurate and robust skeletons. In *Proc. 8th Pacific Conference on Computer Graphics and Applications* 281–449 (IEEE, 2000).

[67] Maitin-Shepard, J. et al. google/neuroglancer. *Zenodo*10.5281/ZENODO.5573294 (2021).

[68] Loomba, S. *Connectomic Comparison of the Human and Non-Human Primate Cortex to Mouse*. PhD thesis, Radboud Univ. (2023).

