# Supplementary material for: Light-microscopy-based connectomic reconstruction of mammalian brain tissue
Source: Nature. 2025 May 7;642(8067):398–410. doi: 10.1038/s41586-025-08985-1 (PMC12158774; doi:10.1038/s41586-025-08985-1)
Supplement: Supplementary file 1 — PDF file containing Supplementary Tables 1–8 and Supplementary Figs. 1–25, providing further information on LICONN optimization, analysis and application. [file 41586_2025_8985_MOESM1_ESM.pdf]

nature portfolio

https://doi.org/10.1038/s41586-025-08985-1

Supplementary information

Light-microscopy-based connectomic reconstruction of mammalian brain tissue

In the format provided by the authors and unedited

Supplementary Information

Light-microscopy based connectomic reconstruction of mammalian brain tissue

Mojtaba R. Tavakoli[1], Julia Lyudchik[1], Michał Januszewski[2], Vitali Vistunou[1], Nathalie Agudelo Dueñas[1], Jakob Vorlaufer[1], Christoph Sommer[1], Caroline Kreuzinger[1], Bárbara Oliveira[1], Alban Cenameri[1], Gaia Novarino[1], Viren Jain[3], Johann G. Danzl[1*]

[1]Institute of Science and Technology Austria; Klosterneuburg, Austria. [2]Google Research; Zürich, Switzerland. [3]Google Research; Mountain View, CA, USA.

*correspondence to: johann.danzl@ista.ac.at

Table of contents

	page
**Supplementary Tables**	
Supplementary Table 1	2
Supplementary Table 2	3
Supplementary Table 3	4
Supplementary Table 4	6
Supplementary Table 5	6
Supplementary Table 6	7
Supplementary Table 7	9
Supplementary Table 8	9
**Supplementary Figures**	
Supplementary Figure 1	11
Supplementary Figure 2	13
Supplementary Figure 3	14
Supplementary Figure 4	15
Supplementary Figure 5	17
Supplementary Figure 6	18
Supplementary Figure 7	19
Supplementary Figure 8	20
Supplementary Figure 9	21
Supplementary Figure 10	23
Supplementary Figure 11	25
Supplementary Figure 12	27
Supplementary Figure 13	29
Supplementary Figure 14	31
Supplementary Figure 15	33
Supplementary Figure 16	35
Supplementary Figure 17	37
Supplementary Figure 18	38
Supplementary Figure 19	39
Supplementary Figure 20	41
Supplementary Figure 21	43
Supplementary Figure 22	45
Supplementary Figure 23	46
Supplementary Figure 24	47
Supplementary Figure 25	48

Supplementary Table 1| List of chemicals

Compound	Abbreviation	Vendor	Identifier
Acrylamide	AA	Sigma-Aldrich/Merck	A9099
Sodium acrylate	SA	AK Scientific	R624
N,N′-Methylenebisacrylamide	BIS	Sigma-Aldrich/Merck	M7279
Ammonium persulfate	APS	Sigma-Aldrich/Merck	A3678
N,N,N',N'- tetramethyl-ethylenediamine	TEMED	Sigma-Aldrich/Merck	T9281
4-Hydroxy-2,2,6,6-tetramethylpiperidin-1	TEMPO	Sigma-Aldrich/Merck	176141
Sodium dodecyl sulfate	SDS	Sigma-Aldrich/Merck	436143
Sodium chloride	----	Sigma-Aldrich/Merck	71383
Tris 1 M, pH 8.0, RNase-free	----	ThermoFisher Scientific/ Invitrogen	AM9856
Paraformaldehyde	PFA	Sigma-Aldrich/Merck	158127
Glycine	----	Sigma-Aldrich/Merck	50046
Sodium azide	----	Sigma-Aldrich/Merck	71289
Ketamine 100 mg/ml	----	MSD Tiergesundheit	NA
Xylazine 20 mg/ml	Xylasol	Livisto	NA
Novalgine 0.5 mg/ml	Metamizol	Sanofi	NA
Isoflurane	----	Virbac/ Vetflurane	NA
Glycerol triglycidyl ether (Glycidyl Glycerol-Ether, Polyfunctional)	TGE	Polysciences Europe GmbH	09221
Glycidyl Methacrylate (Glycidyl Acrylate)	GMA	TCI	G0497
Sodium bicarbonate	$NaHCO_3$	Sigma-Aldrich/Merck	792519
Tween-20	Tween	Fisher BioReagents	BP337
Sodium hydroxide	NaOH	Sigma Aldrich/Merck	S5881
Poly-L-Lysine Hydrochloride	PLL-HCl	Sigma-Aldrich/Merck	P2658
Acrylic acid N-hydroxysuccinimide ester	NAS	Sigma-Aldrich/Merck	A8060
6-((acryloyl)amino)hexanoic acid, succinimidyl ester	AcX	ThermoFisher Scientific	A20770
4’6-diamidino-2- phenylindole dihydrochloride	DAPI	Sigma-Aldrich/Merck	D9542

Structures of chemicals

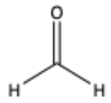

Formaldehyde (FA) Molecular weight (MW) 30.03 (g/mol)

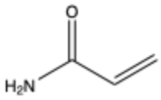

Acrylamide (AA), MW 71.08 (g/mol)

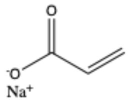

Sodium acrylate (SA), MW 94.04 (g/mol)

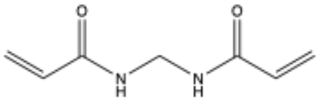

N,N‘-Methylenebisacrylamide (BIS), MW 154.17 (g/mol)

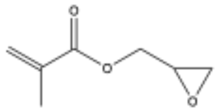

Glycidyl Methacrylate (GMA), MW 142.15 (g/mol)

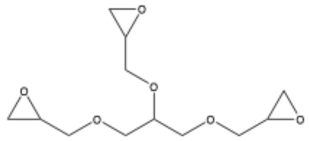

Glycerol triglycidyl ether (TGE), MW 260.29 (g/mol)

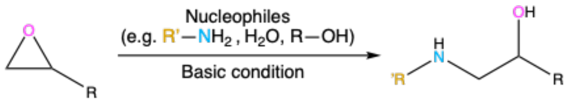

Epoxide ring opening under basic condition

Supplementary Table 2| List of solutions

Solution	Abbr.	Composition	Concentration
1X Phosphate Buffered Saline, pH 7.4	1X PBS	Sodium Phosphate dibasic (Na_2HPO_4)	10.00 mM
		Potassium Phosphate Monobasic (KH_2PO_4)	1.98 mM
		Potassium chloride (KCl)	2.68 mM

		Sodium chloride (NaCl)	136.89 mM
Milli-Q water, pH 7.4	milli-Q water	Ultrapure water (Milli-Q, EQ7000)	---
Sodium bicarbonate, pH 8.5	100 mM $NaHCO_3$	Sodium bicarbonate ($NaHCO_3$)	100 mM
		Ultrapure water	---
Denaturation buffer, pH 9.0	DB	Sodium dodecyl sulfate (SDS)	200 mM
		Sodium chloride (NaCl)	200 mM
		TRIS-HCl	50 mM
		Ultrapure water	---
Perfusion solution, pH 7.4	PS	4% Paraformaldehyde (PFA)	4 g/ 100 ml
		10% Acrylamide (AA)	10 g/ 100 ml
		1X PBS	---

Supplementary Table 3| List of primary antibodies used in the manuscript

Target, host species	Abbr.	Vendor	Identifier	Clonality	Working dilution
Anti-Bassoon antibody, Mouse	Anti-Bsn	Synaptic Systems	141 011	Monoclonal	1:300
Anti-Bassoon antibody, Rabbit	Anti-Bsn	Synaptic Systems	141 003	Polyclonal	1:300
Anti-RIM-1/2 antibody, Guinea pig	Anti-RIM-1/2	Synaptic Systems	140 205	Polyclonal	1:300
Anti-Munc13-1 antibody, Rabbit	Anti-Munc13-1	Synaptic Systems	126 103	Polyclonal	1:300
Anti-vesicular glutamate transporter 1 antibody, Rabbit	Anti-vGlut1	Synaptic Systems	135 302	Polyclonal	1:300
Anti-vesicular gamma-aminobutyric acid transporter antibody, Rabbit	Anti-vGAT	Synaptic Systems	131 003	Polyclonal	1:200
Anti-Ca^{2+} P/Q antibody, Guinea pig	Anti- Ca^{2+} P/Q	Synaptic Systems	152 205	Polyclonal	1:300
Anti-NMDA type glutamate receptor antibody, Mouse	Anti-GluN1	Synaptic Systems	114 011	Monoclonal	1:300
Anti-Postsynaptic density-95 antibody, Mouse	Anti-PSD95	ThermoFisher/ Invitrogen	MA1-046	Monoclonal	1:400

Anti-Shank2 antibody, Guinea pig	Anti-Shank2	Synaptic Systems	162 204	Polyclonal	1:300
Anti-Shank3 antibody, Guinea pig	Anti-Shank3	Synaptic Systems	162 304	Polyclonal	1:300
Anti-Shank1/2/3 antibody, Mouse	Anti-Shank1/2/3	Santa Cruz Biotechnology	sc-393963	Monoclonal	1:100
Anti-Gephyrin antibody, Mouse	Anti-Gephyrin	Synaptic Systems	147 111	Monoclonal	1:300
Anti-Gephyrin antibody, Mouse	Anti-Gephyrin	Santa Cruz Biotechnology	sc-25311	Monoclonal	1:200
Anti-glial fibrillary acidic protein antibody, Mouse	Anti-GFAP	Synaptic Systems	173 011	Monoclonal	1:300
Anti-glial fibrillary acidic protein antibody, Rabbit	Anti-GFAP	ThermoFisher/ Invitrogen	PA1-10019	Polyclonal	1:400
Anti-Vimentin antibody, Guinea pig	Anti-Vimentin	Synaptic Systems	172 004	Polyclonal	1:300
Anti-Somatostatin antibody, Mouse	Anti-SST	Sanza Cruz Biotechnology	sc-74556	Monoclonal	1:100 (used in cocktail, low signal)
Anti-Somatostatin antibody, Rat	Anti-SST	Sanza Cruz Biotechnology	sc-47706	Monoclonal	1:100 (used in cocktail, low signal)
Anti-Somatostatin antibody, Chicken	Anti-SST	Synaptic Systems	366 006	Polyclonal	1:300 (used in cocktail, low signal)
Anti-Somatostatin antibody, Rat	Anti-SST	Sigma-Aldrich/Merck	MAB354	Monoclonal	1:100 (used in cocktail, low signal)
Anti-Somatostatin antibody, Mouse	Anti-SST	Sanza Cruz Biotechnology	sc-55565	Monoclonal	1:100 (used in cocktail, low signal)
Anti-K_V3.1b, Rabbit	Anti-K_V3.1b	Synaptic Systems	242003	Polyclonal	1:300
Anti-Acetylated tubulin antibody, Mouse	Anti-a-Tubulin	Sigma-Aldrich/Merck	T7451	Monoclonal	1:200

Anti-Adenylate cyclase-3 antibody, Rabbit	Anti-AC3	NOVUS Biologicals	NBP1-92683	Polyclonal	1:200
Anti-Ankyrin G antibody, Guinea pig	Anti-Ankyrin G	Synaptic Systems	386 005	Polyclonal	1:300
Anti-Green fluorescent protein antibody, Rabbit	Anti-GFP	ThermoFisher/ Invitrogen	A-11122	Polyclonal	1:300
Anti-Myelin basic protein antibody, Mouse	Anti-MBP	BioLegend	808403	Monoclonal	1:200
Anti-Connexin43 antibody, Rabbit	Anti-CnX-43	Sigma-Aldrich/Merck	C6219	Polyclonal	1:200
Anti-Pmp70 antibody, Rabbit	Anti-Pmp70	Abcam	ab85550	Polyclonal	1:400

Supplementary Table 4| List of secondary antibodies

**Target**	**Species**	**fluorophore**	**Vendor**	**Identifier**	**Working dilution**
Mouse IgG (H+L)	Goat	Alexa Fluor 488	Thermo Fisher/ Invitrogen	A-11001	1:400
Mouse IgG (H+L)	Goat	Alexa Fluor 546	Thermo Fisher/ Invitrogen	A-11030	1:400
Mouse IgG (H+L)	Goat	STAR RED	Abberior	STRED-1001	1:200
Rabbit IgG (H+L)	Goat	Alexa Fluor Plus 488	Thermo Fisher/ Invitrogen	A-32731	1:400
Rabbit IgG (H+L)	Goat	Alexa Fluor 546	Thermo Fisher/ Invitrogen	A-11035	1:400
Rabbit IgG	Goat	STAR RED	Abberior	STRED-1002	1:200
Guinea pig IgG	Goat	STAR RED	Abberior	STRED-1006	1:200
Rat IgG	Goat	STAR 635 P	Abberior	ST635p-1007	1:100
Rat IgG	Goat	STAR 580	Abberior	ST580-1007	1:100
Chicken IgG	Goat	Alexa Fluor 594	Thermo Fisher/ Invitrogen	A-11042	1:300

Supplementary Table 5| Composition of LICONN hydrogels.

	Acrylamide (AA) (% w/v)	Sodium Acrylate (SA) (% w/v)	N,N′-Methylenebisacrylamide (BIS) (% w/v)	APS (% w/v)	TEMED (% v/v)	TEMPO (% w/v)
1st expandable hydrogel	10	12.5	0.075	0.15	0.15	0.001

Stabilizing hydrogel	10	----	0.025	0.05	0.05	----
2nd expandable hydrogel	10	19	0.025	0.05	0.05	----

Supplementary Table 6| Additional antibodies tested

Testing was performed after the first hydrogel. Dedicated optimization of labelling conditions may yield satisfactory labelling results with antibodies that show no or poor labelling under the standard conditions tested here.

Target, host species	Vendor	Identifier	Clonality	Tested working dilution and Batch concentration	Labelling after 1st hydrogel
Anti-alpha/beta SNAP-25, Rabbit	Synaptic Systems	111 002	Polyclonal	1:300<br>N/A	No
Anti-bFos, Mouse	Santa Cruz Biotech.	sc-398595	Monoclonal	1:100<br>0.2 mg/ml	No
Anti-cFos, Rabbit	Synaptic Systems	226 008	Monoclonal	1:50<br>1 mg/ml	No
Anti-cFos, Rabbit	Cell Signaling Technology	2250	Monoclonal	1:50<br>0.1 mg/ml	No
Anti-Connexin-35/36, Mouse	Merck	MAB3045	Monoclonal	1:100<br>1 mg/ml	No
Anti-Connexin-36, Rabbit	Thermo Fisher	51-6200	Polyclonal	1:200<br>0.25 mg/ml	No
Anti-Connexin-36, Mouse	Santa Cruz Biotech.	sc-398063	Monoclonal	1:100<br>0.2 mg/ml	No
Anti-Connexin-45, Rabbit	Thermo Fisher	40-7000	Polyclonal	1:200<br>0.25 mg/ml	No
Anti-Dynamin-1/2/3, Rabbit	Synaptic Systems	115 002	Polyclonal	1:300<br>N/A	No
Anti-glial fibrillary acidic protein, Mouse	Santa Cruz Biotech.	sc-33673	Monoclonal	1:200<br>0.2 mg/ml	Yes
Anti-Glutamic acid decarboxylase 1/67, Rabbit	Synaptic Systems	198 208	Monoclonal	1:300<br>1 mg/ml	Dim, poorly/not retained after full expansion

Anti-Glutamic acid decarboxylase 65/67, Mouse	Santa Cruz Biotech.	sc-365180	Monoclonal	1:100<br>0.2 mg/ml	No
Anti-Green fluorescent protein, Mouse	Thermo Fisher	A11120	Monoclonal	1:300<br>1 mg/ml	Yes
Anti-IBA1, Rabbit	Synaptic Systems	234 013	Polyclonal	1:300<br>1 mg/ml	Dim, poorly/not retained after full expansion
Anti-IBA1, Chicken	Synaptic Systems	234 009	Monoclonal	1:300<br>1 mg/ml	Dim, poorly/not retained after full expansion
Anti-Myelin basic protein, Mouse	Santa Cruz Biotech.	sc-271524	Monoclonal	1:100<br>0.2 mg/ml	Yes
Anti-Nucleoporin-98, Rabbit	Cell Signaling Technology	2598	Monoclonal	1:200<br>0.018 mg/ml	Yes
Anti-Parvalbumin, Guinea pig	Synaptic Systems	195 004	Polyclonal	1:200<br>N/A	No
Anti-Parvalbumin, Guinea pig	Synaptic Systems	195 308	Monoclonal	1:100<br>1 mg/ml	No
Anti-Parvalbumin, Rabbit	Synaptic Systems	195 002	Polyclonal	1:100<br>N/A	Yes
Anti-Piccolo, Guinea pig	Synaptic Systems	142 104	Polyclonal	1:300<br>N/A	Yes
Anti-S100B, Guinea pig	Synaptic Systems	287 004	Polyclonal	1:300<br>N/A	No
Anti-Synapatophysin 1, Guinea pig	Synaptic Systems	101 004	Polyclonal	1:300<br>N/A	No
Anti-Synaptic vesicle 2A protein, Guinea pig	Synaptic Systems	119 004	Polyclonal	1:300<br>N/A	No
Anti-Synaptophysin, Rabbit	Cell Signaling Technology	36406	Monoclonal	1:100<br>0.006 mg/ml	No

Anti-Synaptotagmin1 Guinea pig	Synaptic Systems	105 015	Polyclonal	1:300<br>1 mg/ml	No
Anti-vesicular GABA transporter, Chicken	Synaptic Systems	131 006	Polyclonal	1:200<br>1 mg/ml	Yes

Supplementary Table 7| List of NHS-coupled fluorophores

Compound	Abbr.	Vendor	Identifier	Working concentration
N-hydroxysuccinimidyl-ester Alexa Fluor 488	NHS-AF 488	Jena Bioscience (Click Chemistry Tools, Vectorlabs)	APC-002-1 (Click Chemistry Tools: 1338)	40 µM
N-hydroxysuccinimidyl-ester Atto 488	NHS-Atto 488	Atto-Tec	AD 488-31	

Supplementary Table 8| Composition of hydrogels.

Hydrogels used in various optimization steps as indicated in the respective Supplementary Figures. For LICONN, compositions #11 and #14 were chosen for the first and second expandable hydrogels, respectively, with BIS concentration adjusted to 0.025% for the second expandable hydrogel.

ID	Acrylamide (%w/v)	Sodium acrylate (%w/v)	N,N´-Methylenebisacrylamide (%w/v)
	AA	SA	BIS
#1	4	5.3	0.075
#2	4	7	
#3	6	7	
#4	7	7	
#5	10	7	
#6	12.5	7	
#7	15	7	
#8	20	7	
#9	10	10	
#10	14	10	
#11	10	12.5	
#12	12.5	12.5	
#13	10	15	
#14	10	19	
#15	2.5	8.6	
#16	12.5	10	

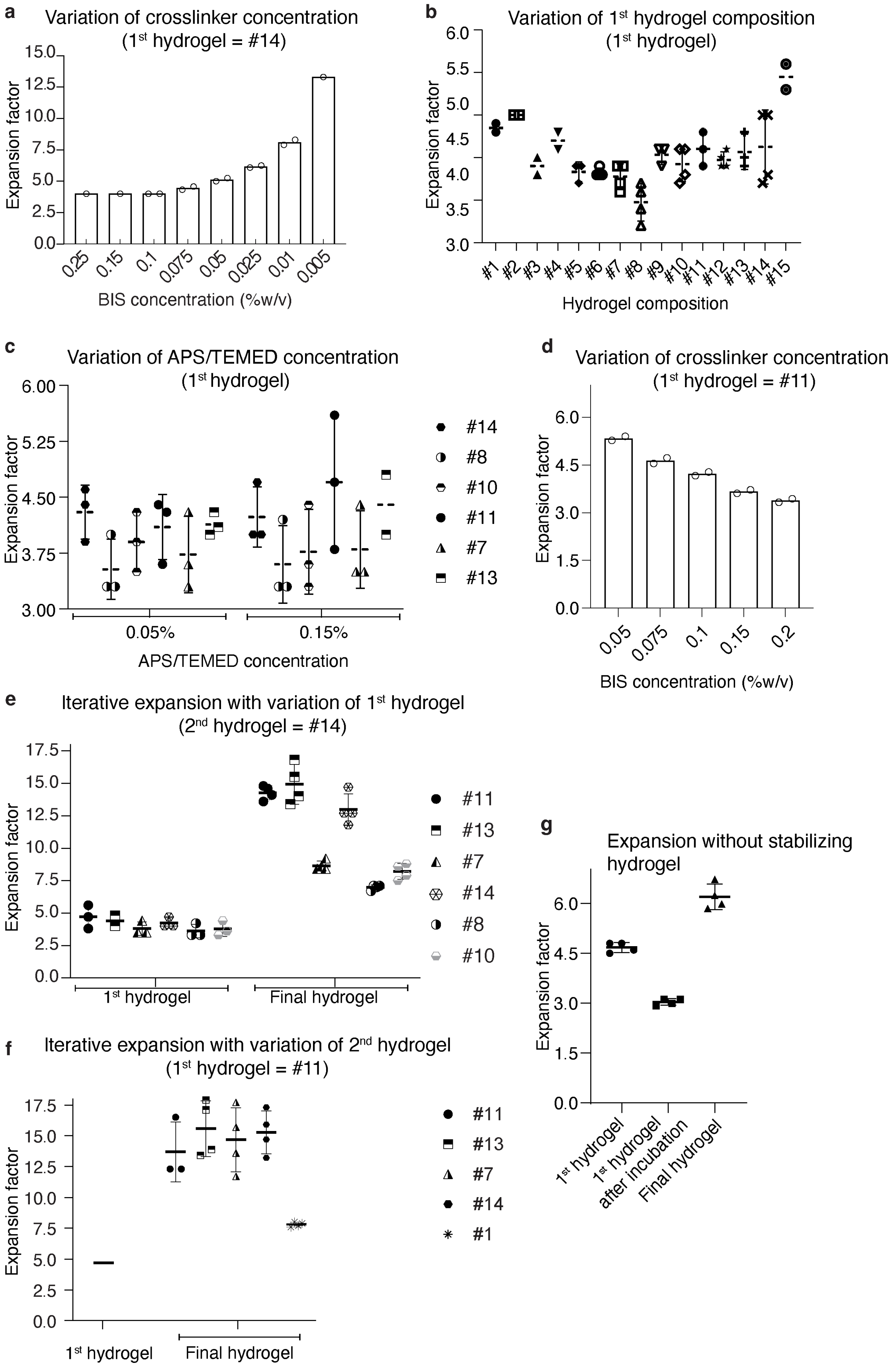

Supplementary Fig. 1| Optimization of hydrogel compositions. a, Expansion factor in single-step expansion as a function of crosslinker (BIS) concentration. Hydrogel composition as in hydrogel #14 (see **Supplementary Table 8**) but with variation of BIS concentration.

Reduction of crosslinker concentration increased exF but resulted in mechanically unstable hydrogels. **b,** ExF of the first expandable hydrogel for different hydrogel compositions according to **Supplementary Table 8**. Hydrogel composition #11 was chosen for the first expandable hydrogel in LICONN, taking additional factors like expansion fidelity and mechanical stability into account. For example, hydrogel #15 resulted in a mechanically unstable hydrogel. **c,** Expansion factor of the first expandable hydrogel for different concentrations of polymerization initiator (APS) and accelerator (TEMED), used at equal concentrations, for different hydrogel compositions according to **Supplementary Table 8**. **d,** Evaluation of expansion factor for the hydrogel composition of the first expandable hydrogel (composition #11) in LICONN, as a function of crosslinker concentration. **e,** Expansion factor after the first and second expansion steps in iterative expansion as a function of composition of the first expandable hydrogel. The composition of the second expandable hydrogel was kept constant, using the one chosen for LICONN (#14). Hydrogel neutralization was performed with 0.2% or 1% APS/TEMED. **f,** Expansion factor after the first and second expansion steps in iterative expansion as a function of composition of the second expandable hydrogel. The composition of the first expandable hydrogel was kept constant, using the one chosen for LICONN (#11). For testing composition of the second expandable hydrogel, one initial hydrogel (exF=4.7) was subdivided and further expanded according to the stated recipes for the second expandable gel. **g,** Effect of omitting the stabilizing hydrogel. Expansion factor after the first expansion step, after incubation with the monomer solution for the second expandable hydrogel (when omitting the stabilizing hydrogel), and after polymerizing and expanding the second swellable hydrogel. Expansion factor was drastically reduced with respect to the LICONN procedure. Compositions of first and second expandable hydrogels were identical to the LICONN procedure. Expansion factors were evaluated by measuring hydrogel size with a caliper in technical samples without biological specimens. Graphs include mean values and, where 3 or more technical replicates (see Replication section) were performed, also standard deviation (s.d.). Data points represent individual technical replicates.

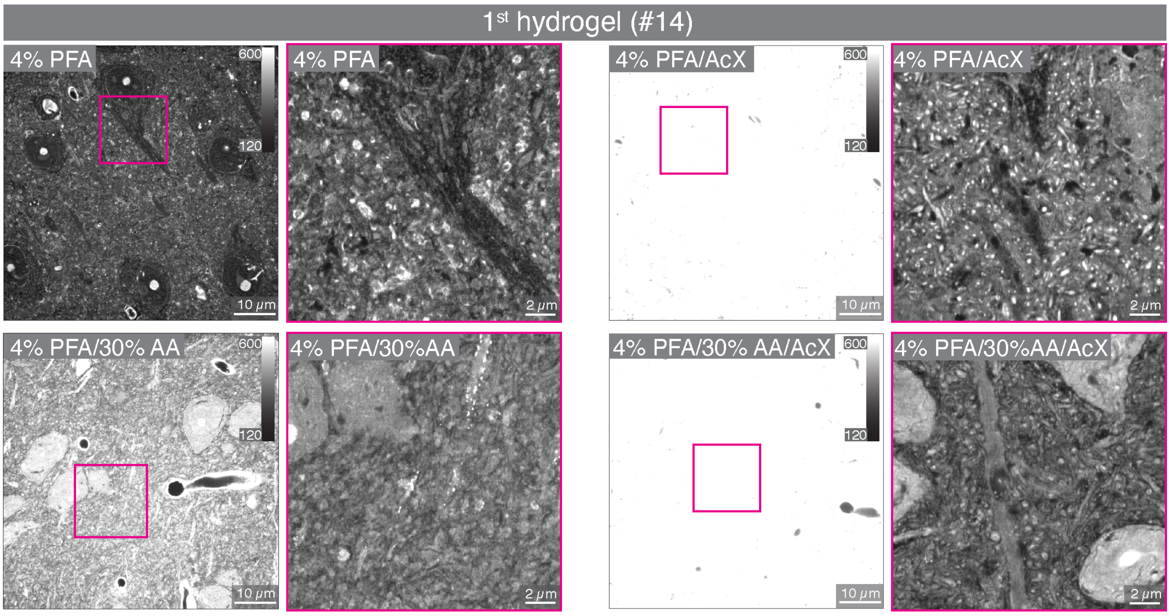

Supplementary Fig. 2| Initial evaluation of protein anchoring. Confocal images with magnified views after first hydrogel expansion using hydrogel composition #14 (**Supplementary Table 8**). Imaging followed transcardial fixative perfusion, postfixation, quenching with glycine, slicing, optional additional anchoring with acryloyl-X (AcX, 100 µg/ml in PBS), hydrogel embedding, denaturation, protein density (pan) labelling, and expansion. *Left, top*: transcardial fixative perfusion with 4% PFA in 1X PBS. *Left, bottom*: Transcardial fixative perfusion with 2% AA in 1X PBS followed by 30% AA and 4% PFA in 1X PBS. Addition of AA increased signal level (same imaging parameters and intensity lookup table in all overview images; adjusted intensity ranges in magnified views). *Right*: Additional anchoring with AcX strongly increased signal level (intensity range saturated in overview images) both for PFA and PFA+AA perfusion. However, we decided against AcX as anchoring agent, as the strong anchoring was paralleled with compromises in preservation of cellular structure. Data representative of *n*=3 technical replicates.

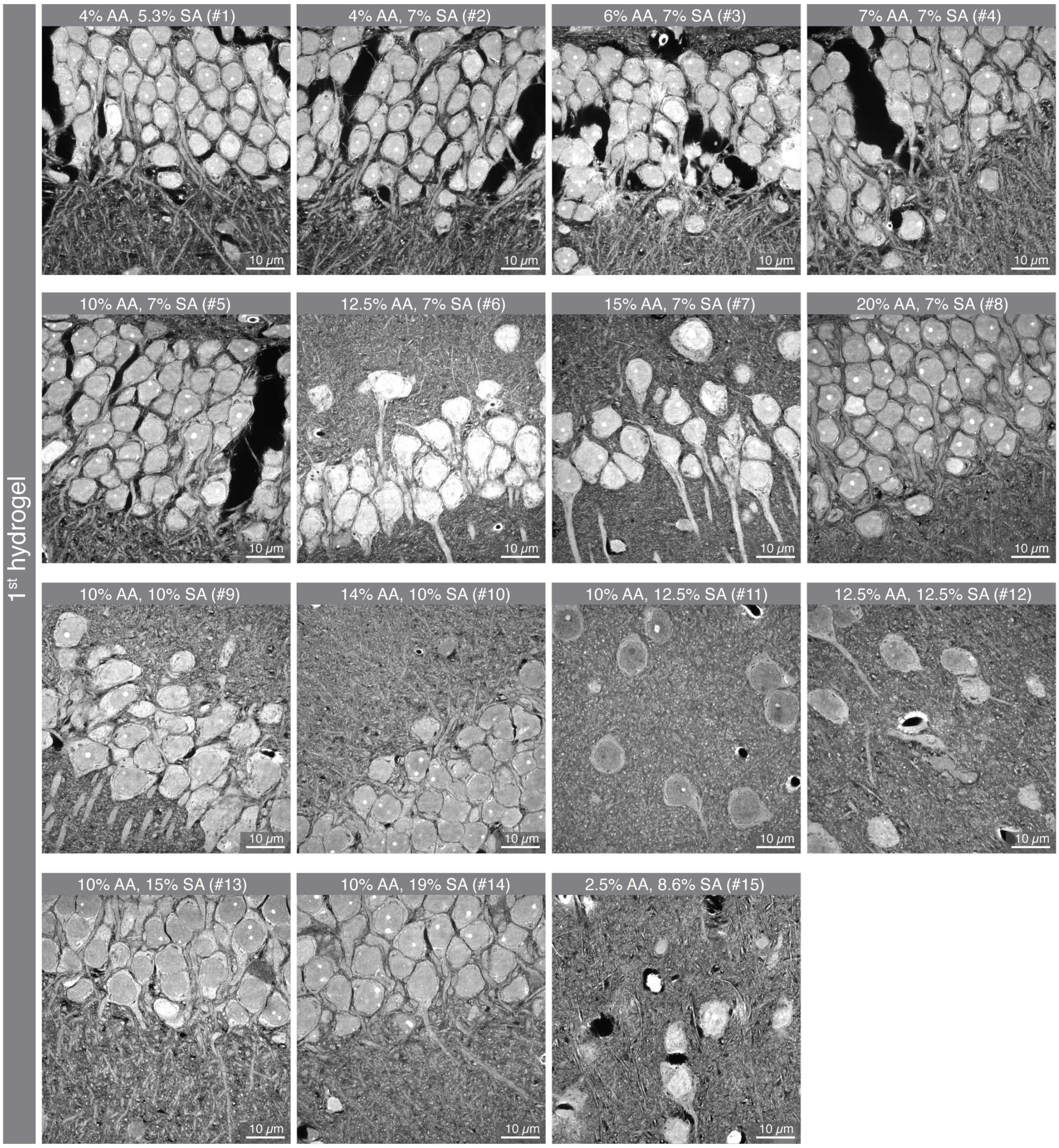

Supplementary Fig. 3| Tissue preservation as a function of the composition of the first expandable hydrogel. Confocal images after the first expansion step for different hydrogel compositions according to **Supplementary Table 8** in hippocampus (#1, #2, #3, #4, #5, #6, #7, #8, #9, #10, #13) or cortex (#11, #12, #15). For transcardial fixative perfusion, previously used conditions were employed in this measurement (perfusion with 2% AA in PBS, followed by 30% AA plus 4 % PFA in PBS)[22]. Brains were post-fixed overnight in the same solution at 4 °C. After slicing, samples were quenched with glycine and incubated with acryloyl-X (100 μg/ml in PBS, overnight) for anchoring of proteins to the hydrogel. Denaturation was performed for 100 min at 95 °C in the same denaturation buffer as used for LICONN experiments. Protein density (pan) labelling and expansion were performed before imaging. Hydrogel compositions with low AA concentrations resulted in fragile hydrogels and ruptures of the tissue-hydrogel hybrid after expansion (black areas). Each condition was technically replicated *n*=2 times. Hydrogels #14 and #15 were replicated *n*=5 times.

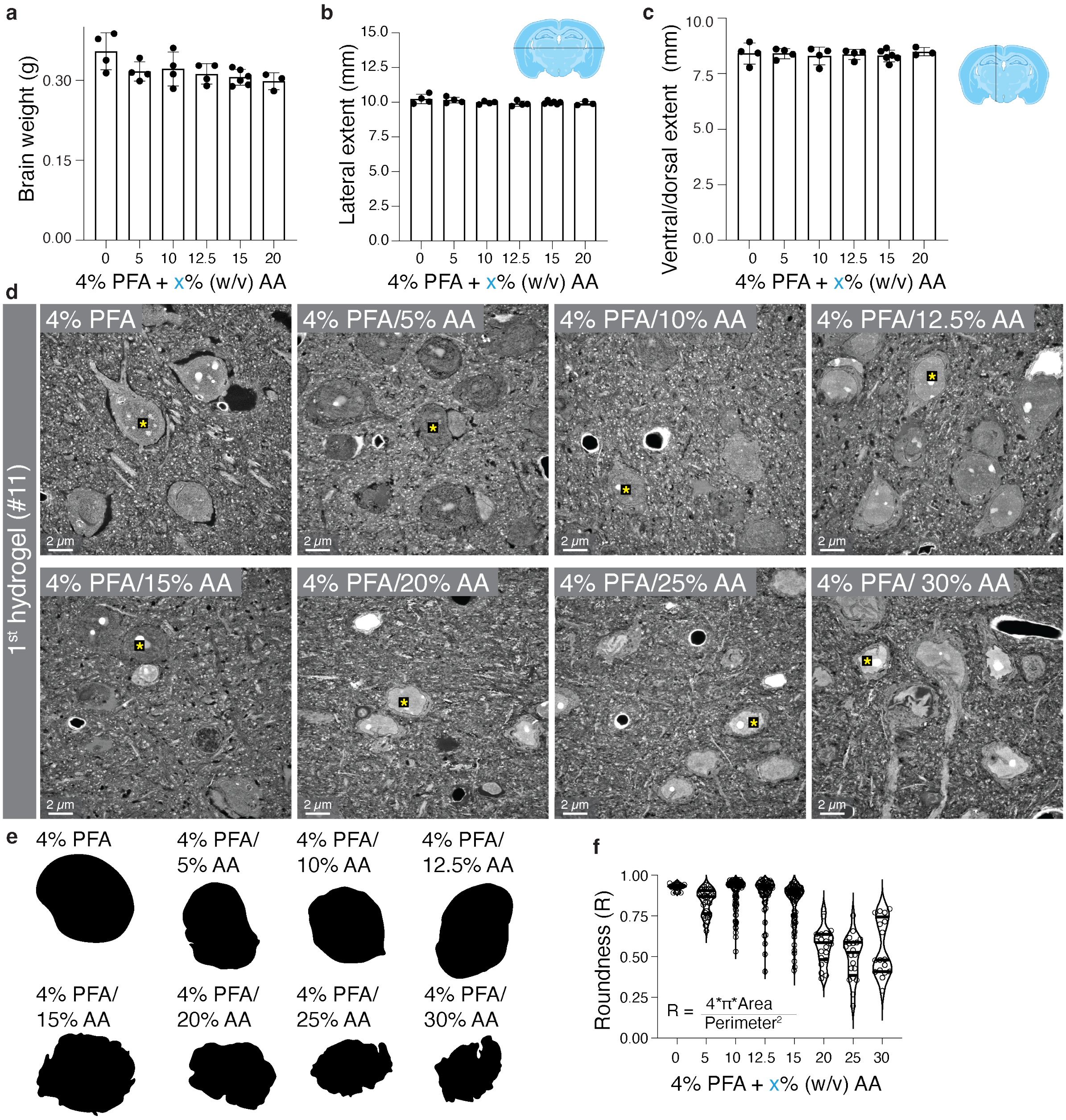

Supplementary Fig. 4| Optimization of transcardial fixative perfusion conditions. a-c, Weight, lateral, and ventral/dorsal extent of brains (mean±s.d.) harvested after transcardial fixative perfusion, first with 1X PBS and then with 4% PFA and varying concentrations of AA. Weight shows a tendency towards lower values with increasing AA concentration. Brain weight was determined after overnight postfixation in the same solution as used in perfusion, and washing in 1X PBS. For quantification of dimensions (see schematic), brains were sliced, stained with DAPI and mounted in Mowiol on a microscopy slide before confocal imaging of corresponding coronal slices across the various conditions. Individual data points represent individual animals. **d,** Confocal images after overnight postfixation in the same solution, slicing, quenching with glycine, hydrogel embedding (composition #11), denaturation at 95 °C for 100 min, protein density (pan) labelling, and expansion. No additional protein anchoring to

the hydrogel was applied. Omission of AA in the transcardial perfusion solution led to voids around cells whereas the highest AA concentrations led to corrugated cell and nuclear outlines. **e,** Shapes of the cell nuclei indicated by asterisks in d. **f,** Quantification of the roundness R of nuclei according to $R=4\pi*area/perimeter^2$. Violin plots include median and lower and upper quartiles. Data points represent individual nuclei.

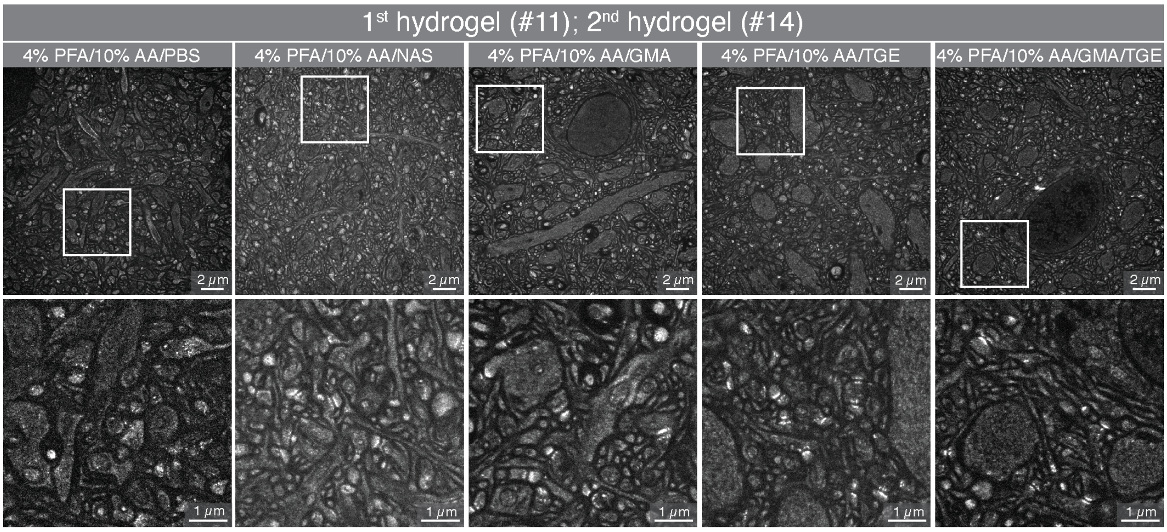

Supplementary Fig. 5| Effect of NAS anchoring and epoxides evaluated after the second expansion step. Overview confocal images and magnified regions as indicated by white boxes in hippocampal CA1 stratum radiatum. The transcardial fixative perfusion procedure (4% PFA and 10 % AA in 1X PBS) and all subsequent steps were performed according to the final LICONN parameters (1st expandable hydrogel: composition #11, 2nd expandable hydrogel: composition #14). After postfixation, slicing and quenching, one of the following steps were performed from left to right: (i) no additional anchoring, (ii) anchoring with NAS (360 µM) for 3h at room temperature, (iii) application of epoxide GMA (3h, 37 °C), (iv) application of epoxide TGE (3h, 37 °C), (v) application of GMA and TGE (3h, 37 °C). Intensity lookup tables were adjusted to account for different overall signal intensities in the images. Both NAS anchoring and epoxide treatment produced high quality datasets whereas omission of these reagents led to reduced signal levels and poorer structural representation. We opted for epoxide treatment as this led to improved signal-to-noise ratio and more pronounced delineation of high protein-density features at synapses. Representative of *n*=3 technical replicates.

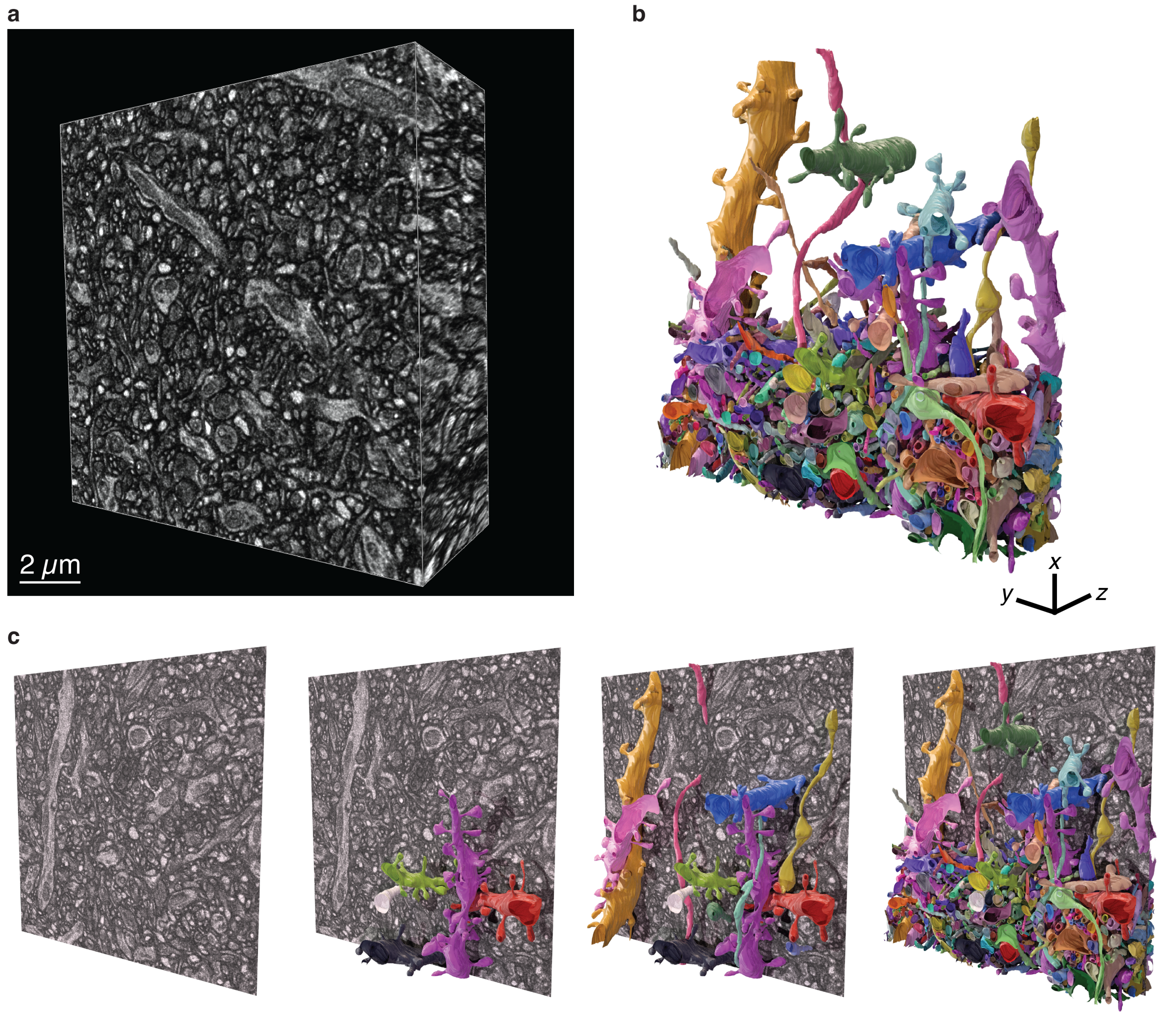

Supplementary Fig. 6| Manual annotation of LICONN volume with NAS anchoring. a, 3D rendering of a 13x13x5 μm³ LICONN volume with NAS anchoring (different from dataset in Fig. 1g) in the hippocampal CA1 stratum oriens. **b,** Rendering of manual annotations of a subset of neuronal structures. The bottom third was densely annotated. **c,** Single confocal imaging plane with progressively increasing number of rendered segments. Transcardial fixative perfusion was performed with 4% PFA, 10 % AA in 1X PBS, followed by postfixation, slicing, glycine quenching, NAS anchoring and hydrogel embedding. The first and second expandable hydrogels had compositions #13 and #14, respectively, according to **Supplementary Table 8**.

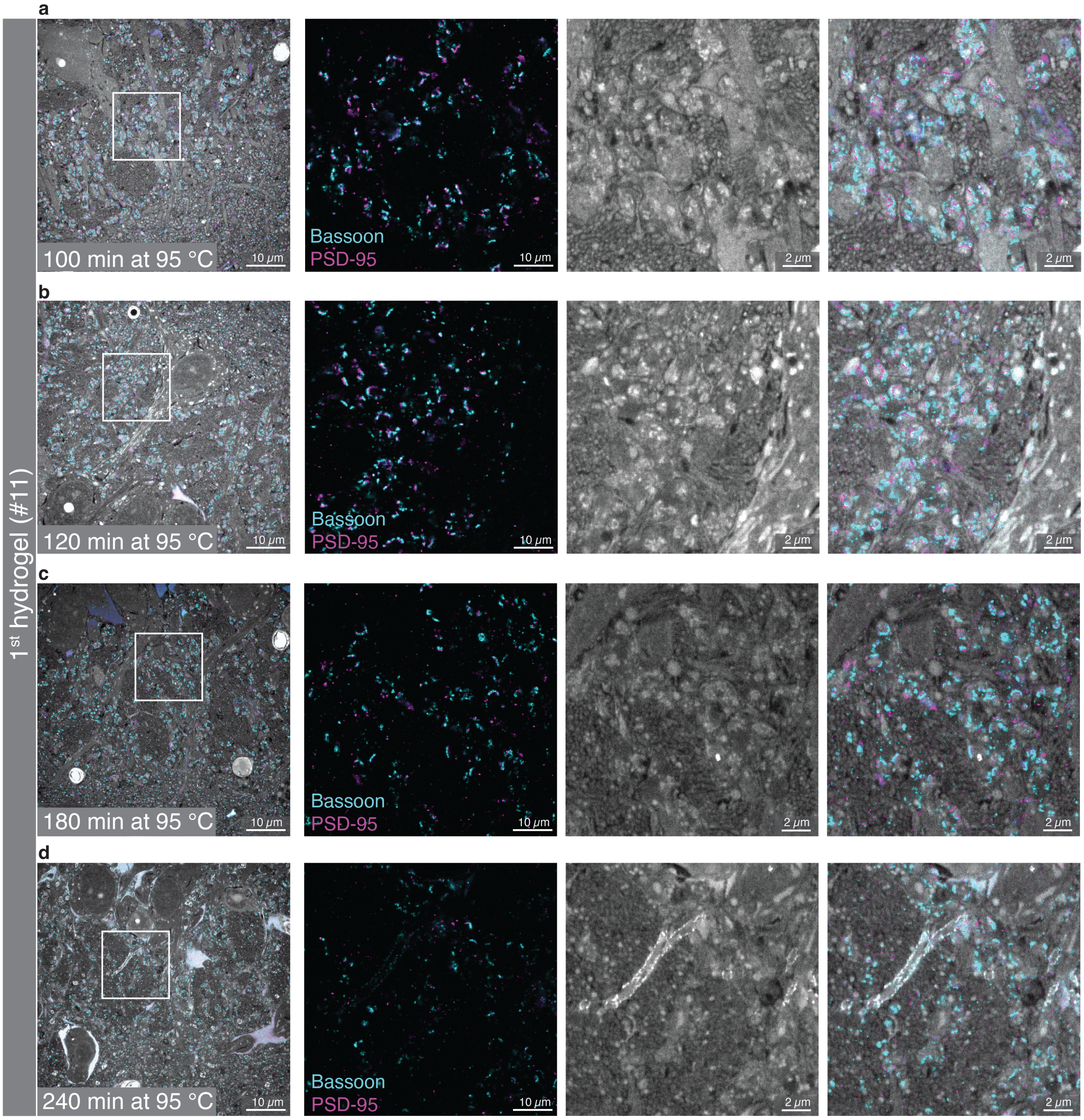

Supplementary Fig. 7| Optimization of denaturation duration. a-d, Confocal images in the hippocampal CA3 stratum lucidum after the first hydrogel expansion step in the LICONN procedure, with protein density (pan) labelling (grey) and immunolabelling for Bassoon (cyan) and PSD-95 (magenta). Denaturation time was varied. Increasing denaturation time led to signal reduction of the protein-density labelling and immunolabelling and compromised structural integrity. Representative of n=3 technical replicates.

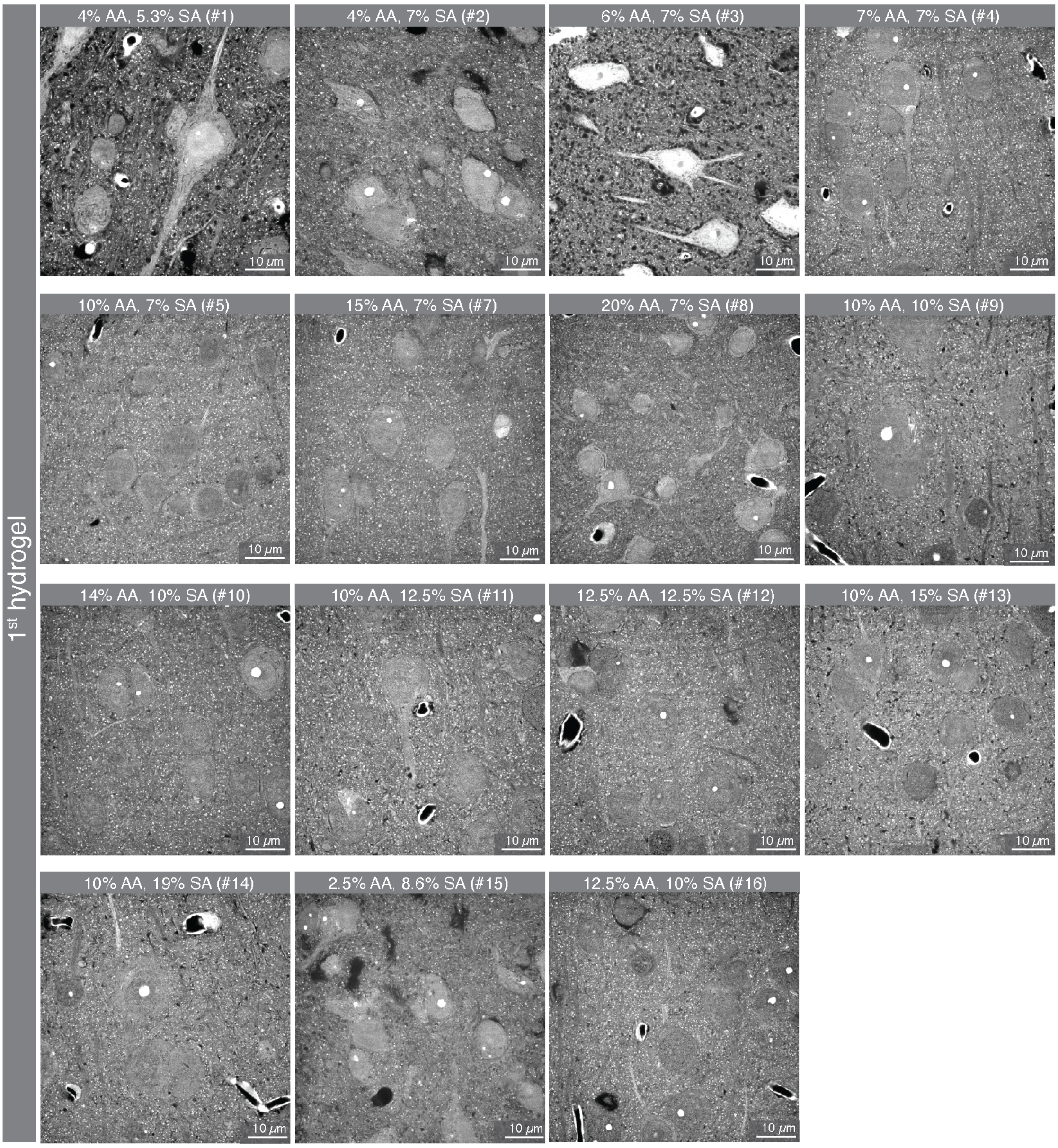

Supplementary Fig. 8| Evaluation of the composition of the first expandable hydrogel, using optimized perfusion and epoxide treatment. Representative confocal images in cortex after the first expansion step and protein density (pan) labelling (grey). Composition of the first expandable hydrogel was varied according to **Supplementary Table 8** while other parameters were identical as in the LICONN procedure. Hydrogels with low AA concentration (e.g. #1, #2, #3, #15) showed compromised integrity and voids throughout the tissue hydrogel hybrid. Representative of *n*=2 technical replicates.

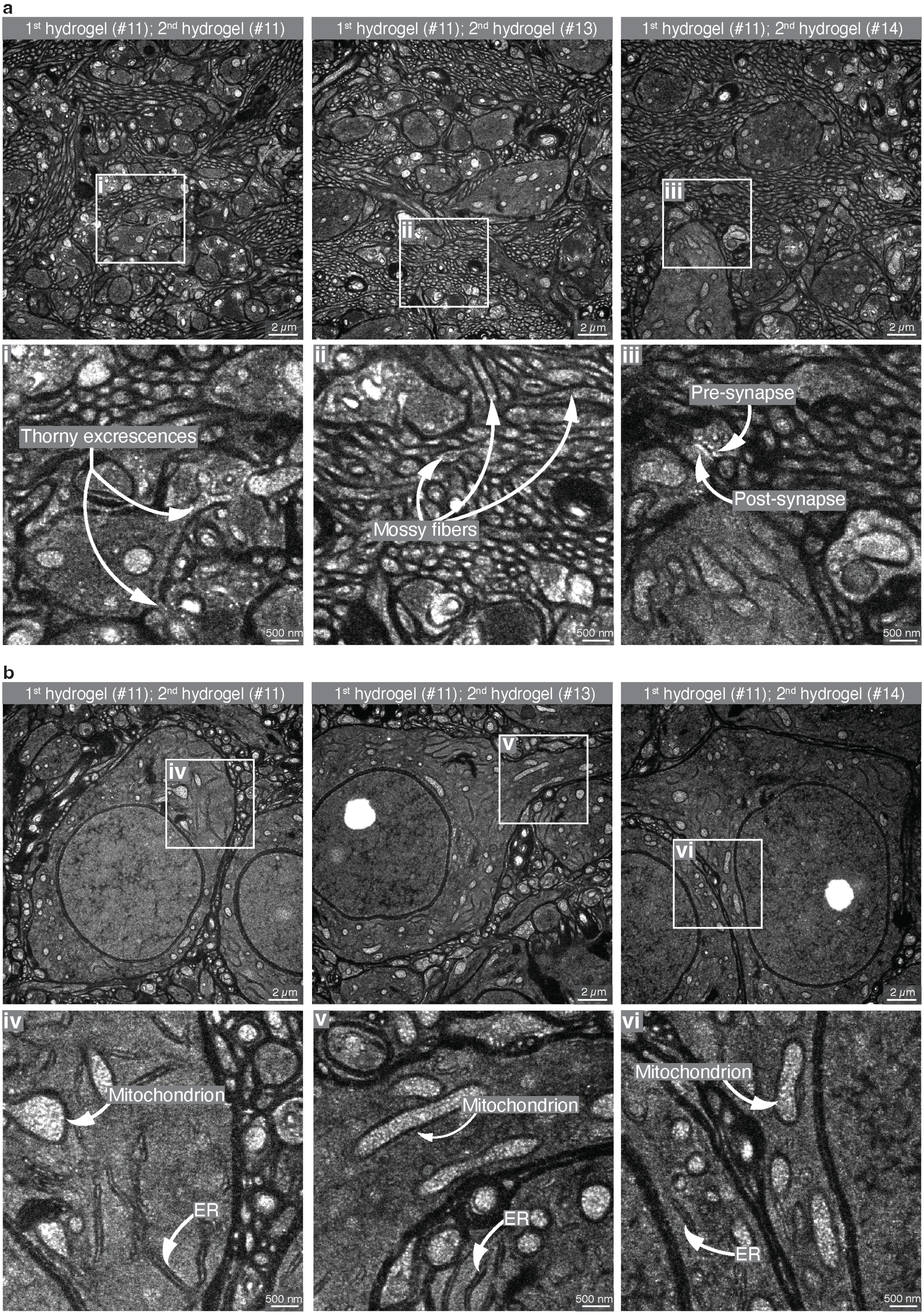

Supplementary Fig. 9| Robustness against variation of parameters. Confocal images after full expansion with optimized denaturation duration, first expandable and stabilizing hydrogels. Perfusion was performed with 4% PFA and 5 % AA in 1X PBS. For protein

anchoring, NAS in PBS (360 μM, 4°C, overnight with shaking) was used. The second expandable hydrogel was varied with compositions according to **Supplementary Table 8**. **a,** Overview and magnified views in the hippocampal CA3 stratum lucidum. Here, mossy fibres, the axons of dentate gyrus (DG) granule cells, are organized in bundles and form excitatory synapses with complex dendritic spines dubbed “thorny excrescences” on proximal dendrites of CA3 pyramidal neurons. **b,** Similar measurements at cell somata in the neighbouring CA3 stratum pyramidale. All displayed conditions produced high-quality datasets. Representative of *n*=3 technical replicates for each condition.

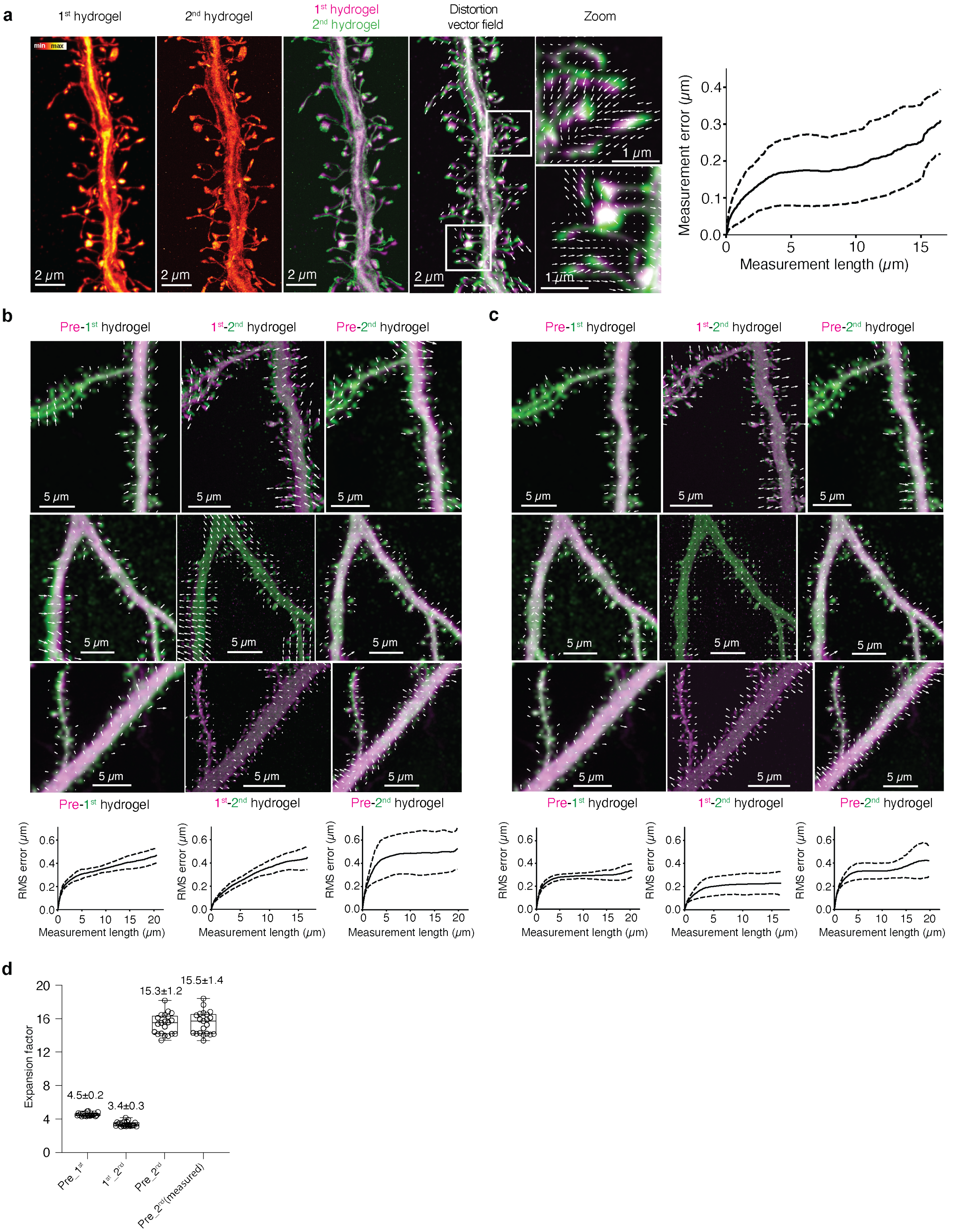

Supplementary Fig. 10| Analysis of expansion-induced distortions and expansion factor. a, Example of distortion analysis, illustrated here for the second LICONN expansion step. *Left to right*: (i) Maximum intensity projection of a confocal imaging volume after the first LICONN expansion step in cortex of a *Thy1-eGFP* mouse, with an eGFP expressing dendrite stretch. The cytosolically expressed eGFP was visualized by immunolabelling. (ii) Maximum intensity projection of the same region after the second LICONN expansion step. Maximum intensity projections were aligned with a similarity transformation (including isotropic scaling,

translation and rotation). (iii) Overlay. (iv) Vector field of distortions during the second expansion step with two magnified views, overlaid with the respective maximum intensity projections. Beyond distortions introduced by the expansion step itself, they may also include additional distortions from manual handling and mounting for imaging. Gaussian smoothing was applied to achieve similar appearance of images before and after expansion for distortion analysis (see Methods). (v) Distortions (mean±s.d. evaluated at given measurement length) as a function of measurement length for this specific measurement. Scale bars: 2 μm, magnified views: 1 μm. Data representative of distortion measurements in 14 fields of view across *n*=4 technical replicates, recorded in cortex across *n*=3 animals. **b,** Distortion vector fields for the first (*left*: pre-expansion to1st hydrogel) and second (*middle*: 1st to 2nd hydrogel) individual expansion steps and for the overall LICONN procedure (right: pre-expansion to 2nd hydrogel). Images represent three examples of measurements used in the analysis. Colour coding in the maximum intensity projections is according to the respective figure headings. Similarity transformation and Gaussian smoothing were applied as in panel a. *Bottom*: Root mean square (RMS) measurement error (mean±s.d. across *n*=4 technical replicates) over different measurement lengths for the first and second expansion steps and for the overall LICONN procedure, evaluated in 14 fields of view across *n*=4 technical replicates, recorded in cortex across *n*=3 animals. Scale bars: 5 μm. **c,** Similar analysis of the same data (mean±s.d. across *n*=4 technical replicates) using an affine transformation (including scaling, translation, rotation and shearing) for initial alignment of maximum intensity projections. Distortion analysis was performed across 14 fields of view in *n*=4 technical replicates, recorded in cortex across *n*=3 animals. **d,** Expansion factor (mean±s.d.) determined as the linear scaling factor in the similarity transformation as in panel a, in 21 imaging volumes analysed across *n*=6 technical replicates from *n*=3 animals. ExF was evaluated for the first (pre_1st) and second (1st_2nd) expansion steps individually. The value obtained as product (pre_2nd) of the exF in the first and second steps was consistent with the value obtained when directly aligning images of pre-expansion and fully expanded hydrogels (pre_2nd (measured)). Box plot: median; lower and upper quartiles; whiskers: min. and max. values. Data points and mean±s.d. refer to individual measurements within the technical replicates.

Scale bars, measurement errors and measurement lengths are scaled to the pre-expansion tissue size throughout. For display purposes, the length of arrows in the distortion vector fields was multiplied by 1.5, while for the magnified views in panel a, the original arrow length is shown.

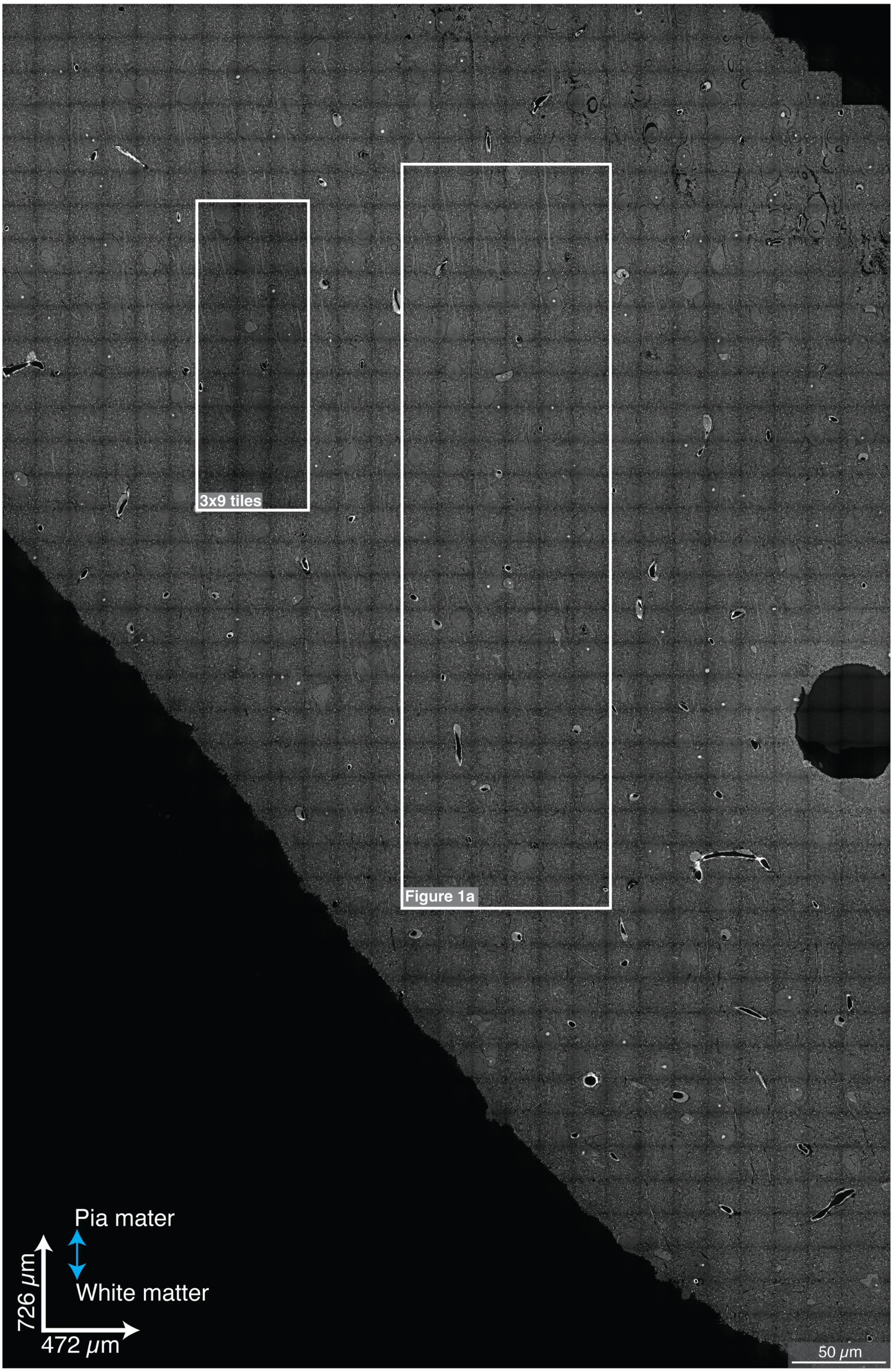

Supplementary Fig. 11| Overview imaging in cortex. Single plane overview image in the region of the somatosensory cortex comprising the imaging volume in **Fig. 1a** as indicated by the boxed region. The lower signal in the region indicated on the left reflects photobleaching

during previous recording of an imaging volume. The dark circular region in the right margin of the image represents a defect in the tissue-hydrogel hybrid, which can occasionally be observed in histological sample processing. Such defects are not specific to LICONN and did not impede our measurements, such that we did not screen samples for defects before imaging. If required for a particular application, such as scaling to much larger volumes, detecting defects would be possible before LICONN imaging by coarse overview scanning of the samples.

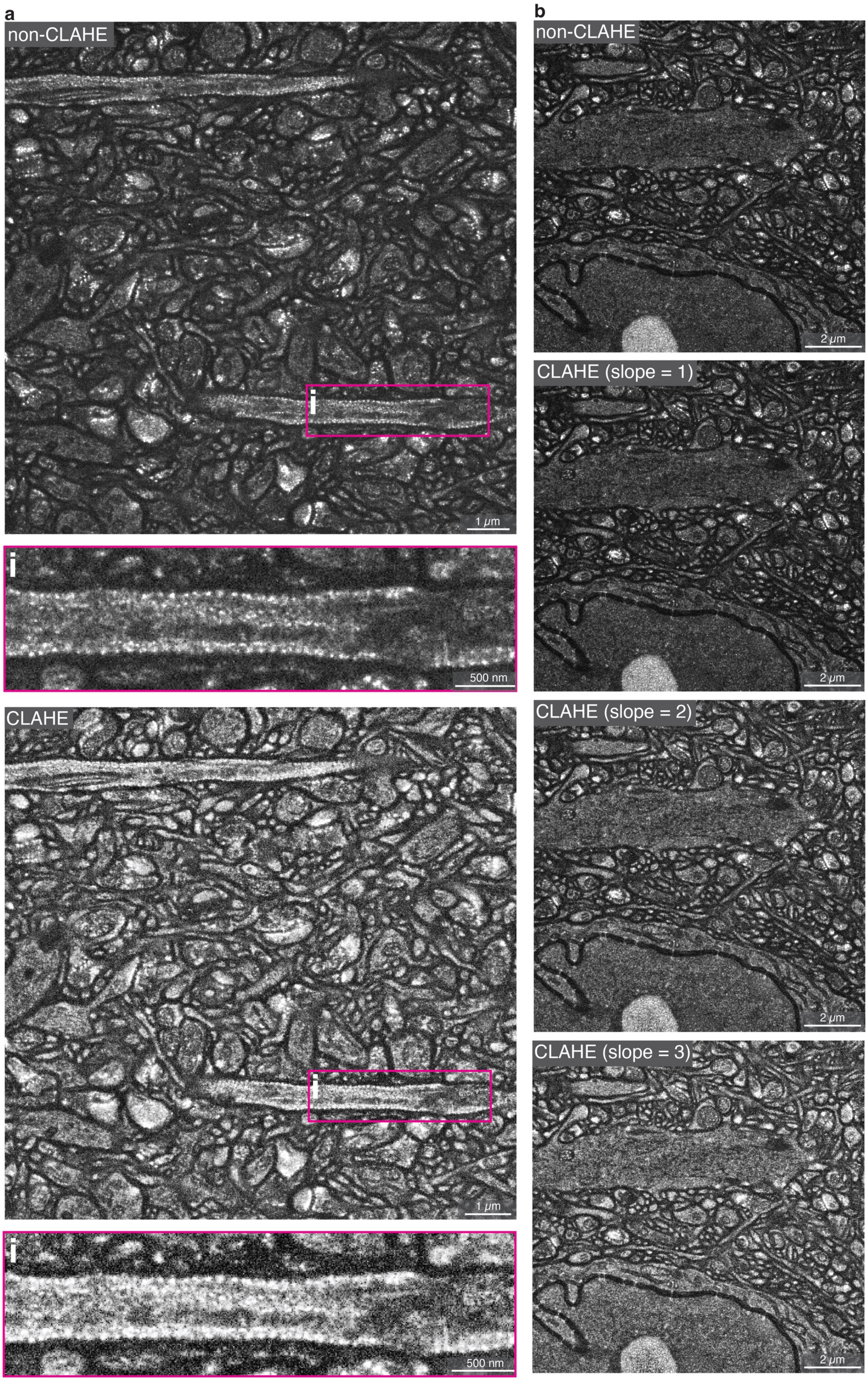

Supplementary Fig. 12| Comparison of CLAHE-processed data with raw imaging data. **a,** *Top:* Raw (non-CLAHE) LICONN imaging data from somatosensory cortex (subregion of a single imaging plane from the dataset in **Fig. 1a**) with magnified view of boxed region,

showing the periodic protein-density modulation induced by the actin-spectrin cytoskeletal rings present in a subset of neurites. *Bottom*: Same data after processing with contrast limited adaptive histogram equalization (CLAHE). High protein-density features at synapses and the periodic lattice stand out more clearly in the raw data. For automated neurite segmentation and display purposes, we used the CLAHE processed data as indicated. **b,** Effect of CLAHE as a function of increasing slope parameter, as implemented in FIJI/ImageJ. Data in panels a and b are representative of application of CLAHE in *n*=10 technical replicates.

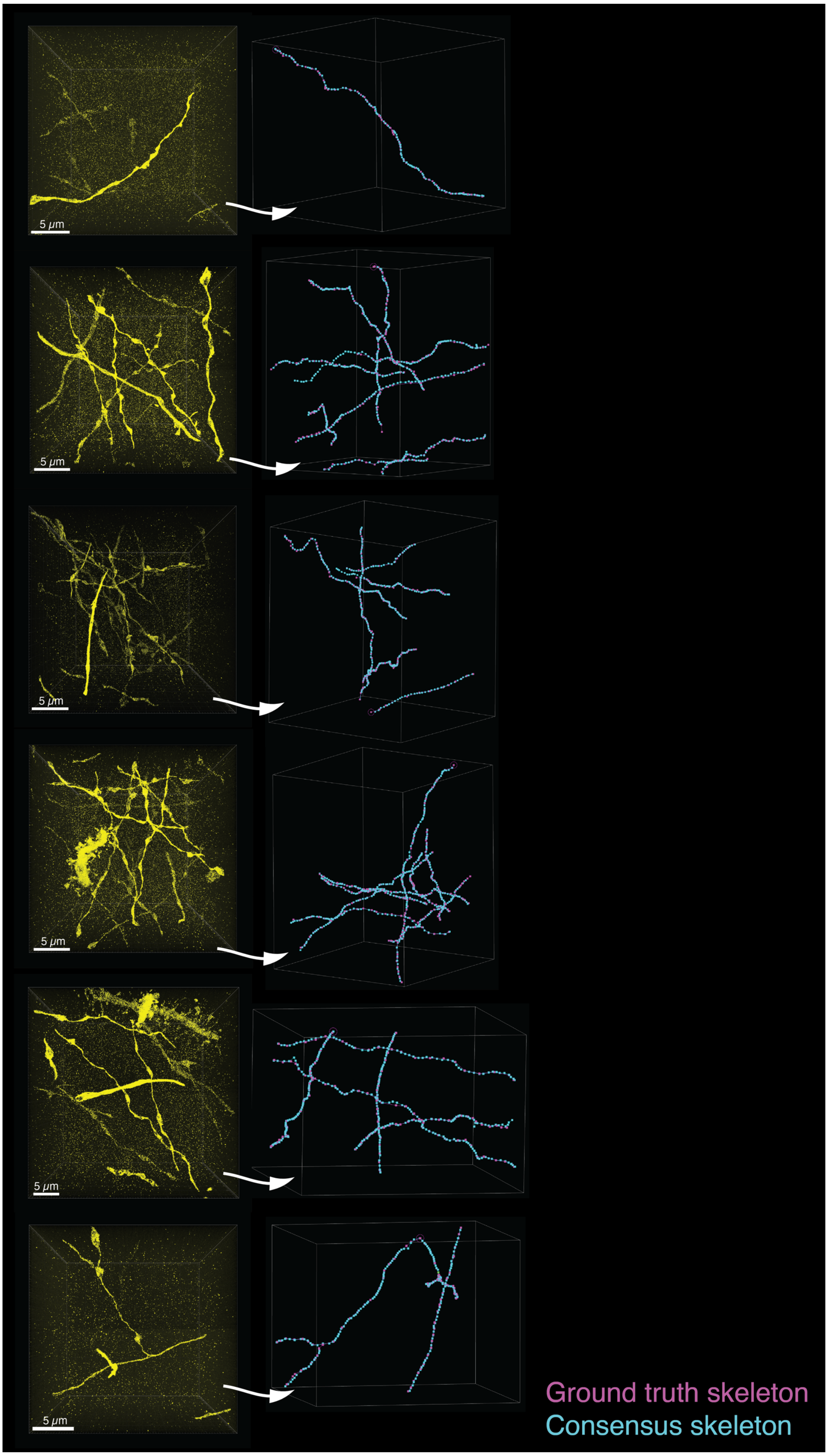

Supplementary Fig. 13| Traceability of axons evaluated against ground truth from sparse eGFP expression (part 1). *Left:* Volumetric renderings of eGFP signal (yellow) in LICONN

volumes recorded in cortex of *Thy1-eGFP* animals with cytosolic expression of eGFP in a sparse subset of neurons. *Right*: Renderings of skeletons of the same structures generated from the LICONN structural channel (consensus skeletons, cyan) by two human annotators blinded to the eGFP channel. These are overlaid with skeletons that were generated by an independent annotator, taking both the LICONN structural channel and the eGFP signal into account (ground truth skeletons, magenta). Note that volume renderings for the eGFP signal and skeletons are from different camera positions. eGFP data was visualized with Imaris software. In total, 37 axon stretches (880 µm cumulative length of eGFP-expressing axons, recorded across n=3 technical replicates across n=2 animals in cortex) were analysed.

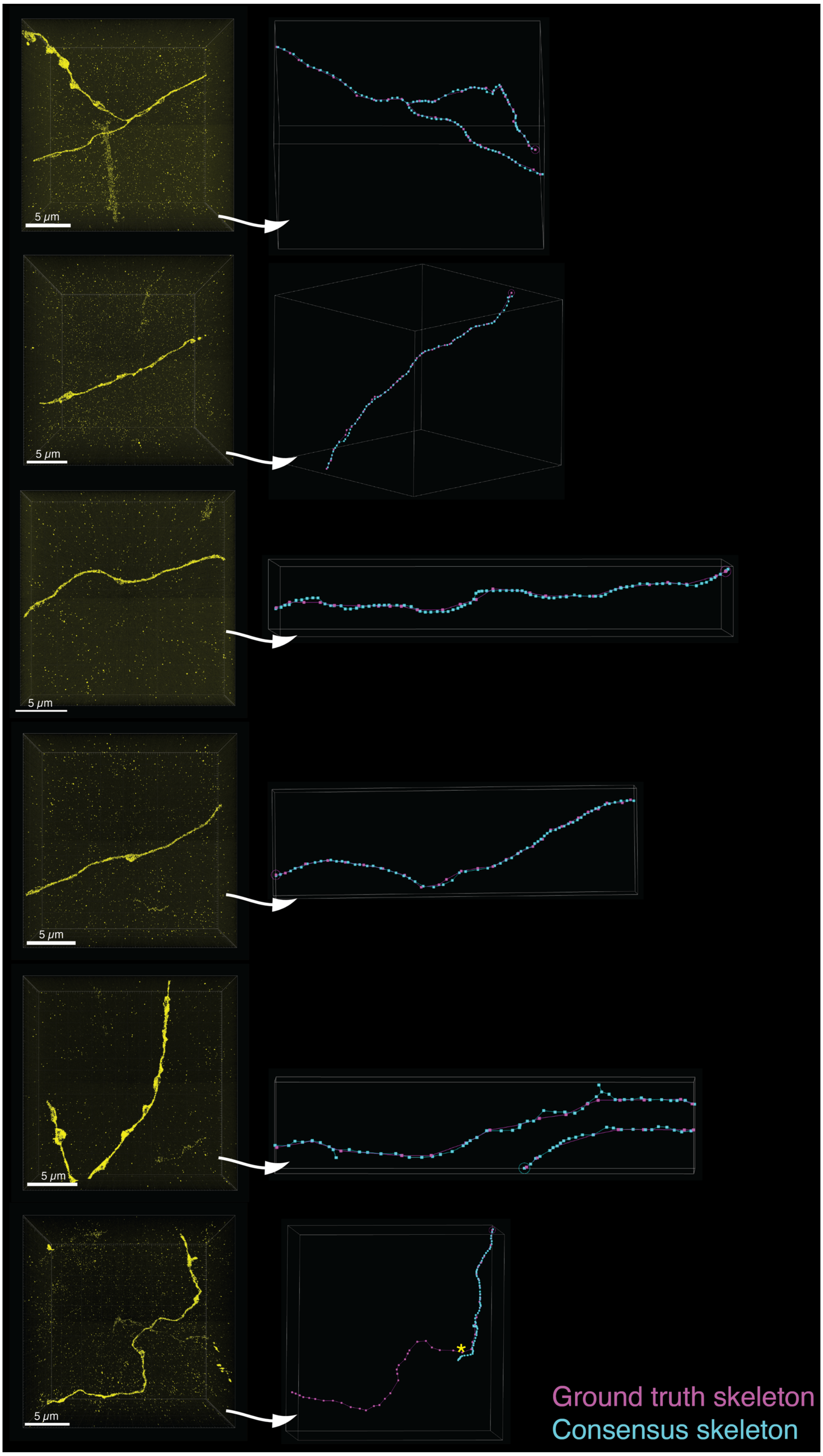

Supplementary Fig. 14| Traceability of axons evaluated against ground truth from sparse eGFP expression (part 2). *Left:* Volumetric renderings of eGFP signal (yellow) in LICONN

volumes recorded in cortex of *Thy1-eGFP* animals with cytosolic expression of eGFP in a sparse subset of neurons. *Right*: Renderings of skeletons of the same structures generated from the LICONN structural channel (consensus skeletons, cyan) by two human annotators blinded to the eGFP channel. These are overlaid with skeletons that were generated by an independent annotator, taking both the LICONN structural channel and the eGFP signal into account (ground truth skeletons, magenta). In the lowest panel, a tracing error (asterisk) occurred in the consensus skeletons generated by the blinded human annotators. Note that volume renderings for the eGFP signal and skeletons are from different camera positions. eGFP data was visualized with Imaris software. In total, 37 axon stretches (880 µm cumulative length of eGFP-expressing axons, recorded across n=3 technical replicates across n=2 animals in cortex) were analysed.

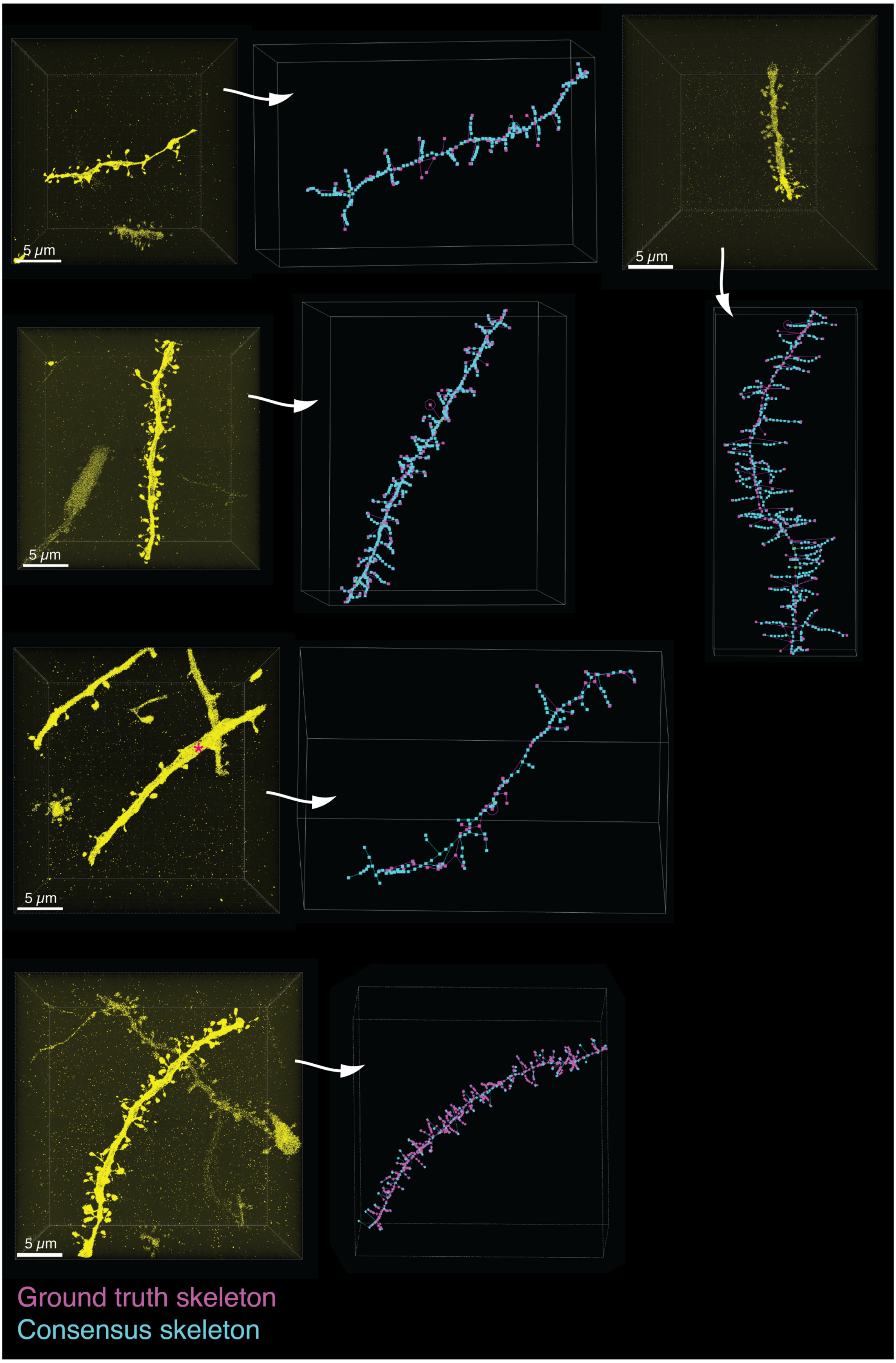

Supplementary Fig. 15| Traceability of dendritic spines evaluated against ground truth from sparse eGFP expression. Volumetric renderings of eGFP signal (yellow) in LICONN

volumes recorded in the hippocampal CA1 region (stratum oriens or stratum radiatum) of *Thy1-eGFP* animals with cytosolic expression of eGFP in a sparse subset of neurons. Renderings of skeletons of the same structures generated from the LICONN structural channel (consensus skeletons, cyan) by two human annotators blinded to the eGFP channel. These are overlaid with skeletons that were generated by an independent annotator, taking both the LICONN structural channel and the eGFP signal into account (ground truth skeletons, magenta). In the second to last volume, the dendrite stretch used for analysis is indicated by an asterisk. Note that camera positions are different in the volume renderings of the eGFP signal and skeletons. eGFP data was visualized with Imaris software. Analysis across 5 datasets from *n*=3 technical replicates across *n*=2 animals.

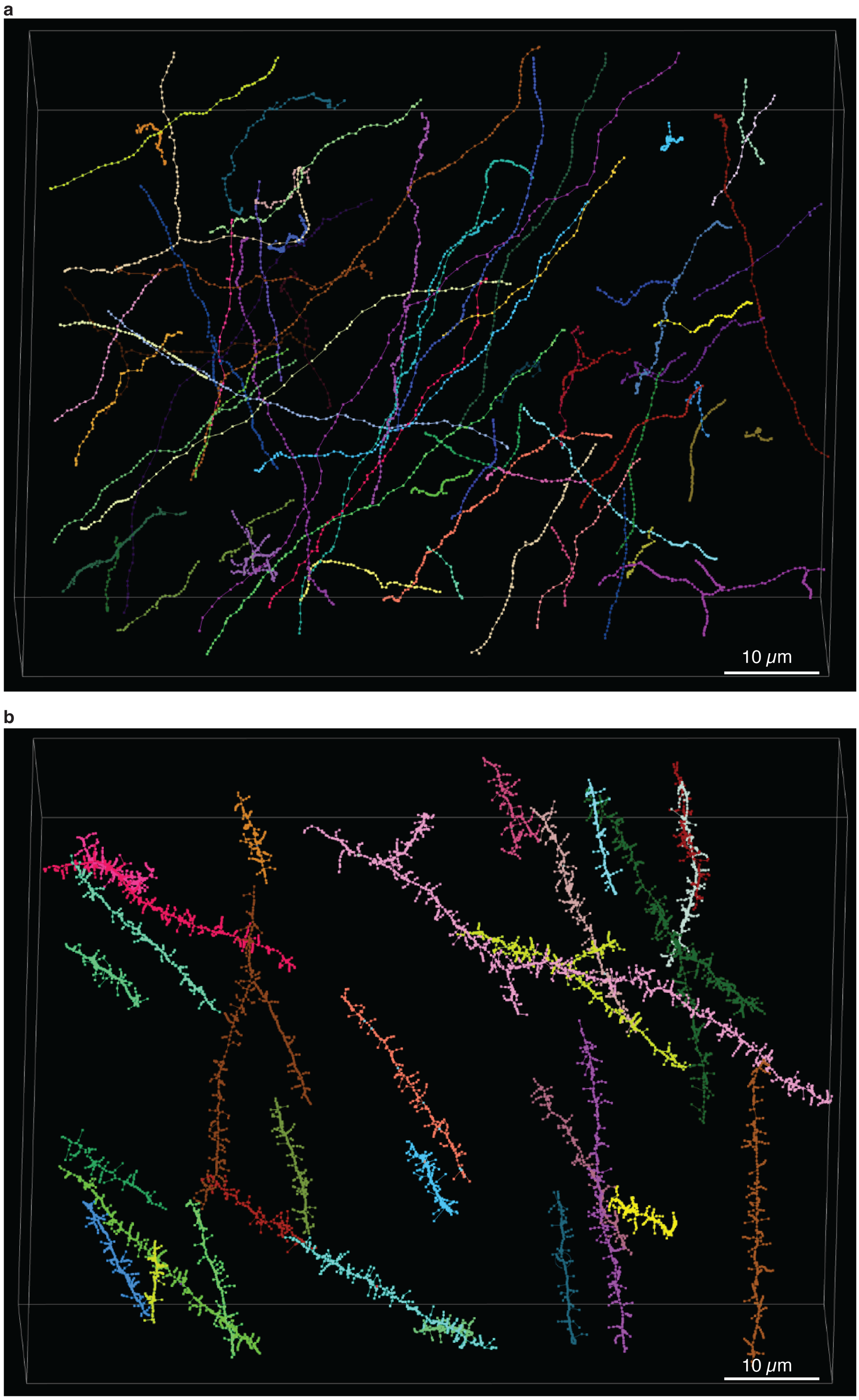

Supplementary Fig. 16| Manual tracing of axons and dendrites in hippocampus. Manually generated skeletons in the dataset in **Fig. 2a**, used for evaluation of automated segmentation

accuracy. Axons and dendrites were automatically selected at random from the output of a semantic classifier (see Methods). **a,** 3D-rendering of the 69 traced axons. **b,** 3D-rendering of the 30 traced dendrite stretches with 1,041 dendritic spines. Dendritic spines are indicated as branches to dendritic shafts.

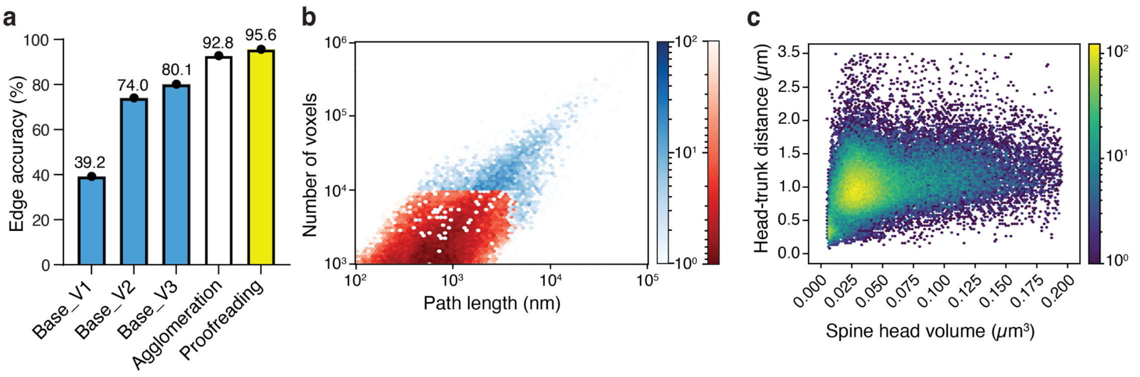

Supplementary Fig. 17| Evaluation of the performance of automated segmentation. a, Edge accuracy for the various stages of FFN segmentation in **Fig. 2**. Blue bars: Base segmentations with models V1-V3 using increasing amounts of training data. White bar: Edge accuracy after automated agglomeration of the base agglomeration using the final model (V3). Yellow bar: Edge accuracy after additional manual proofreading. **b,** Segment size (number of voxels) versus path length of individual segments for the segmentation in **Fig. 2**. Segments that entered the final agglomeration are indicated in blue. Segments below 10,000 voxels volume were disregarded in the agglomeration (red), except for a small number of longer segments with volume below 10,000 voxels. **c,** Scatter plot of head-to-trunk distance vs. spine head volume for dendritic spines in the segmentation volume in **Fig. 2a**. Segmentation with comprehensive proofreading of neuronal structures was performed in n=1 replicate.

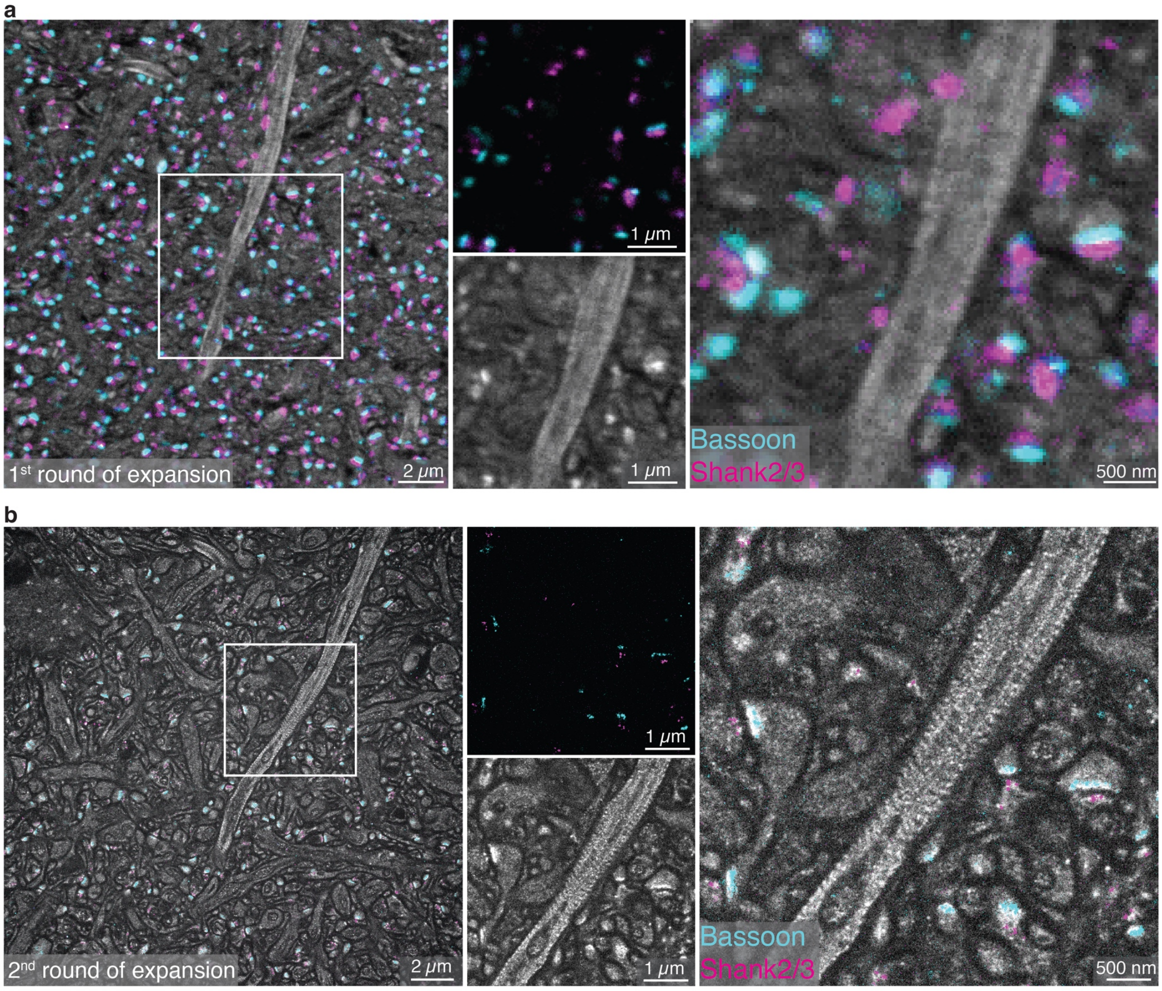

Supplementary Fig. 18| Improved resolution in structural and molecular LICONN channels. a, Confocal image in the hippocampal CA1 region (stratum oriens) after the first LICONN expansion step with immunolabelling for Bassoon (cyan) and Shank2/3 (magenta) overlaid with the structural channel (grey). *Left*: overview. *Middle, right*: Immunolabelling and structural channels shown separately for the region indicated by the box in the left panel and magnified view of the overlaid signal in the same region. **b,** Same region imaged with the same objective lens and microscope after the second expansion step in LICONN. Resolution is increased both for the structural and immunolabelling channels, revealing neurites as separate structures and the periodic protein-density modulation induced by the actin/spectrin cytoskeletal lattice in the prominent neurite in the magnified view. At this resolution, molecular signals can be clearly assigned to individual pre- and post-synapses. Same dataset as in **Fig. 1d**. Visual illustration of resolution improvement in panels a and b was performed in n=1 specimen.

a

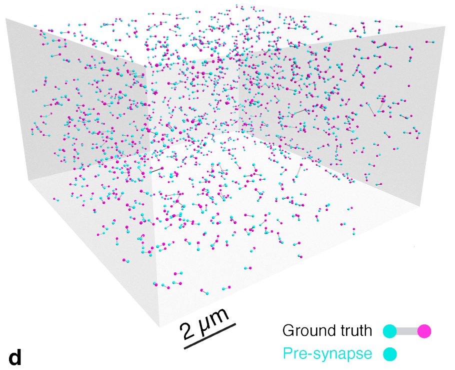

b

Immunolabeling based detection

Pre-synapse detection

	Precision	Recall	F1
Initial	0.96	0.92	0.94
Post-processed	0.95	0.94	0.94

Post-synapse detection

	Precision	Recall	F1
Initial	0.98	0.89	0.93
Post-processed	0.94	0.96	0.95

Full-synapse detection (paired)

	Precision	Recall	F1
Initial	0.93	0.82	0.87
Post-processed	0.89	0.90	0.90

c

Deep-Learning based detection

Pre-synapse detection

	Precision	Recall	F1
Initial	0.95	0.91	0.93
Post-processed	0.95	0.93	0.94

Post-synapse detection

	Precision	Recall	F1
Initial	0.97	0.86	0.92
Post-processed	0.93	0.93	0.93

Full-synapse detection (paired)

	Precision	Recall	F1
Initial	0.94	0.82	0.87
Post-processed	0.90	0.89	0.89

d

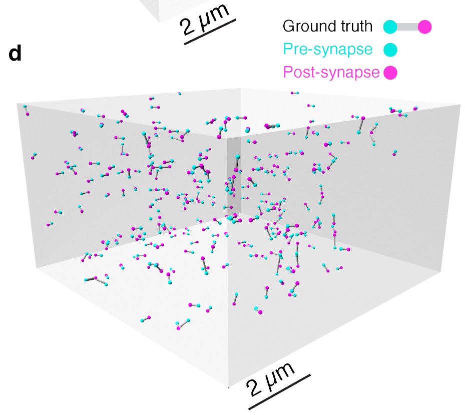

e

Immunolabeling based detection

Pre-synapse detection

	Precision	Recall	F1
Initial	0.92	0.89	0.90
Post-processed	0.92	0.93	0.93

Post-synapse detection

	Precision	Recall	F1
Initial	0.99	0.87	0.92
Post-processed	0.97	0.94	0.95

Full-synapse detection (paired)

	Precision	Recall	F1
Initial	0.94	0.83	0.88
Post-processed	0.92	0.90	0.91

f

Deep-Learning based detection

Pre-synapse detection

	Precision	Recall	F1
Initial	0.97	0.87	0.92
Post-processed	0.97	0.91	0.94

Post-synapse detection

	Precision	Recall	F1
Initial	1.00	0.86	0.93
Post-processed	0.98	0.93	0.95

Full-synapse detection (paired)

	Precision	Recall	F1
Initial	0.97	0.83	0.90
Post-processed	0.95	0.90	0.92

g

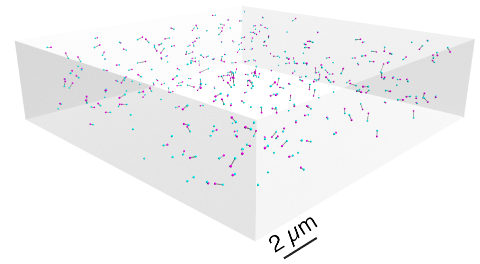

h

Immunolabeling based detection

Pre-synapse detection

	Precision	Recall	F1
Initial	0.86	0.95	0.90
Post-processed	0.84	0.95	0.89

Post-synapse detection

	Precision	Recall	F1
Initial	0.86	0.95	0.90
Post-processed	0.86	0.97	0.91

Full-synapse detection (paired)

	Precision	Recall	F1
Initial	0.89	0.90	0.90
Post-processed	0.86	0.92	0.89

i

Deep-Learning based detection

Pre-synapse detection

	Precision	Recall	F1
Initial	0.92	0.90	0.91
Post-processed	0.92	0.92	0.92

Post-synapse detection

	Precision	Recall	F1
Initial	0.95	0.87	0.90
Post-processed	0.91	0.92	0.91

Full-synapse detection (paired)

	Precision	Recall	F1
Initial	0.94	0.81	0.87
Post-processed	0.89	0.89	0.89

Supplementary Fig. 19| Immunolabelling and deep-learning based detection of excitatory synapses. a, Rendering of ground truth (proofread) immunolabelling-based excitatory synapse detections in the dataset in **Fig. 3h** (hippocampal CA1 region, stratum radiatum, *z*-step size 200 nm, 1,116 pre-synapses, 1,084 post-synapses, forming 1,059 full synapses (with one-to-many connections contributing a count of 1 with each edge), with 57 occurrences of unpaired pre-synapses and 25 unpaired post-synapses). **b,** Precision, recall and F1 score of computational immunolabelling-based detection of pre-synapses, post-synapses, and fully assembled synapses, evaluated using the manually generated ground truth displayed in panel a. Values are given both for the base detection (initial) and with post-processing (see Methods). **c,** Precision, recall and F1 score for deep-learning-based prediction of pre-synapses, post-synapses, and fully assembled synapses from the structural LICONN channel in the same dataset. The dataset was not included in training. **d,** Rendering of ground truth (proofread) immunolabelling based excitatory synapse detections in the dataset in **Fig. 4l** (hippocampal CA1 region, stratum radiatum, *z*-step size 300 nm, 261 pre-synapses, 247 post-synapses, forming 242 full synapses (with one-to-many connections contributing a count of 1 with each edge), with 25 occurrences of unpaired pre-synapses and 7 unpaired post-synapses). **e,** Precision, recall and F1 score of computational immunolabelling-based detection of pre-synapses, post-synapses, and fully assembled synapses, evaluated using the manually

generated ground truth displayed in panel d. **f,** Precision, recall and F1 score for deep-learning-based prediction of pre-synapses, post-synapses, and fully assembled synapses from the structural LICONN channel in the same dataset. The dataset was not included in training. **g,** Rendering of ground truth (proofread) immunolabelling-based excitatory synapse detections in cortex (*z*-step size 300 nm, 306 pre-synapses, 262 post-synapses, forming 261 full synapses (with one-to-many connections contributing a count of 1 with each edge), with 48 occurrences of unpaired pre-synapses and 1 unpaired post-synapse). **h,** Precision, recall and F1 score of computational immunolabelling-based detection of pre-synapses, post-synapses, and fully assembled synapses, evaluated using the manually generated ground truth displayed in panel g. **i,** Precision, recall and F1 score for deep-learning-based prediction of pre-synapses, post-synapses, and fully assembled synapses from the structural LICONN channel in the same dataset. The dataset was not included in training.

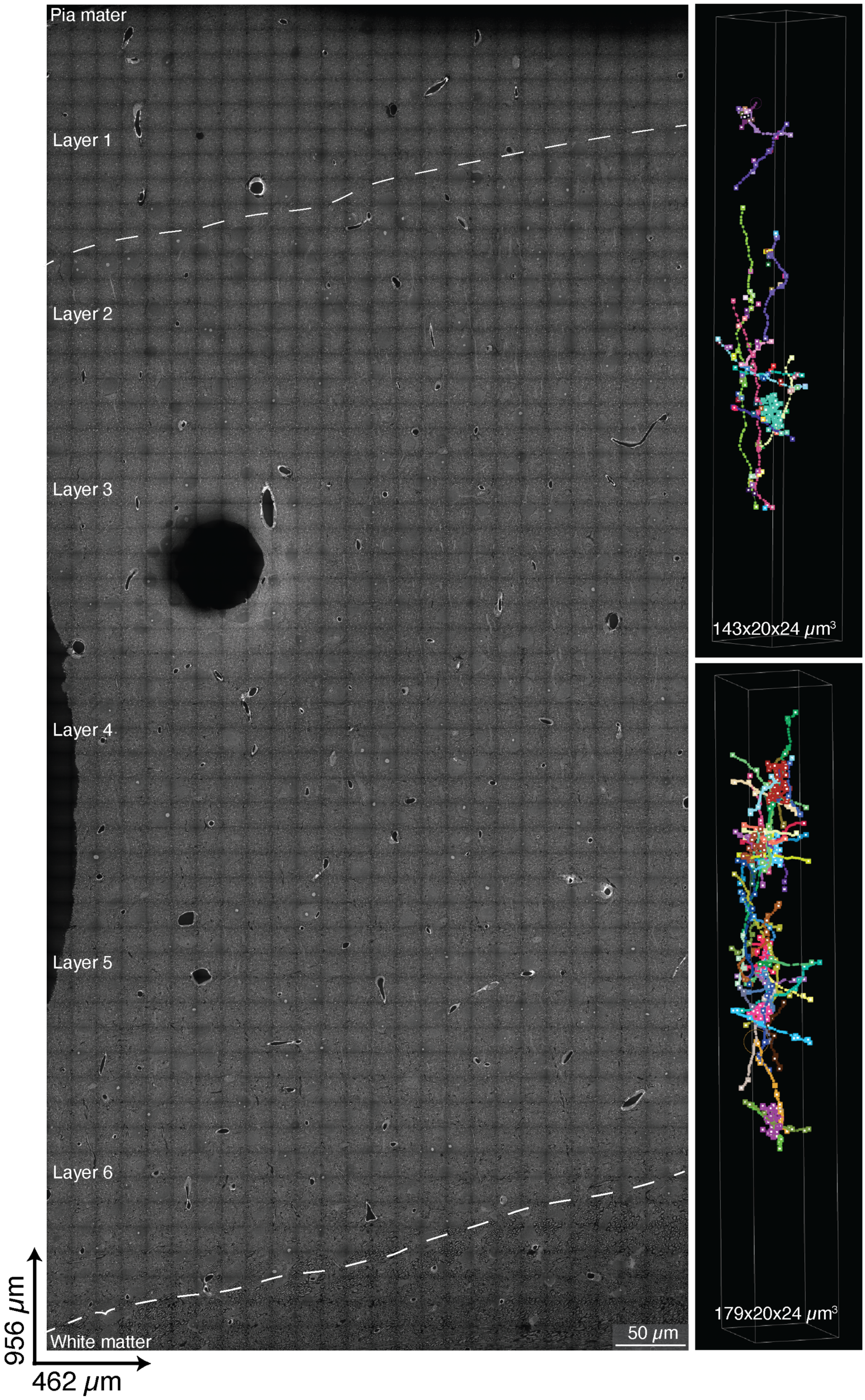

Supplementary Fig. 20| Molecularly informed connectivity analysis (manual tracing). *Left*: Overview of LICONN sample in cortex, spanning from pia mater to the white matter. The

black region likely corresponds to a defect in the hydrogel (e.g. gas bubble) or in sample mounting. *Right*: Manual tracings from the same specimen, used for analysis in **Fig. 4a-k**.

The dark circular region in the centre left represents a defect in the tissue-hydrogel hybrid, which we occasionally observed. Such defects are not specific to LICONN and did not impede our measurements, such that we did not screen samples for defects before imaging. If required for a particular application, such as scaling to much larger volumes, detecting defects would be possible before LICONN imaging by coarse overview scanning of the samples. Manual tracings were performed across 2 imaging volumes from n=1 technical replicate (n=1 animal) imaged in the high-resolution overview scan.

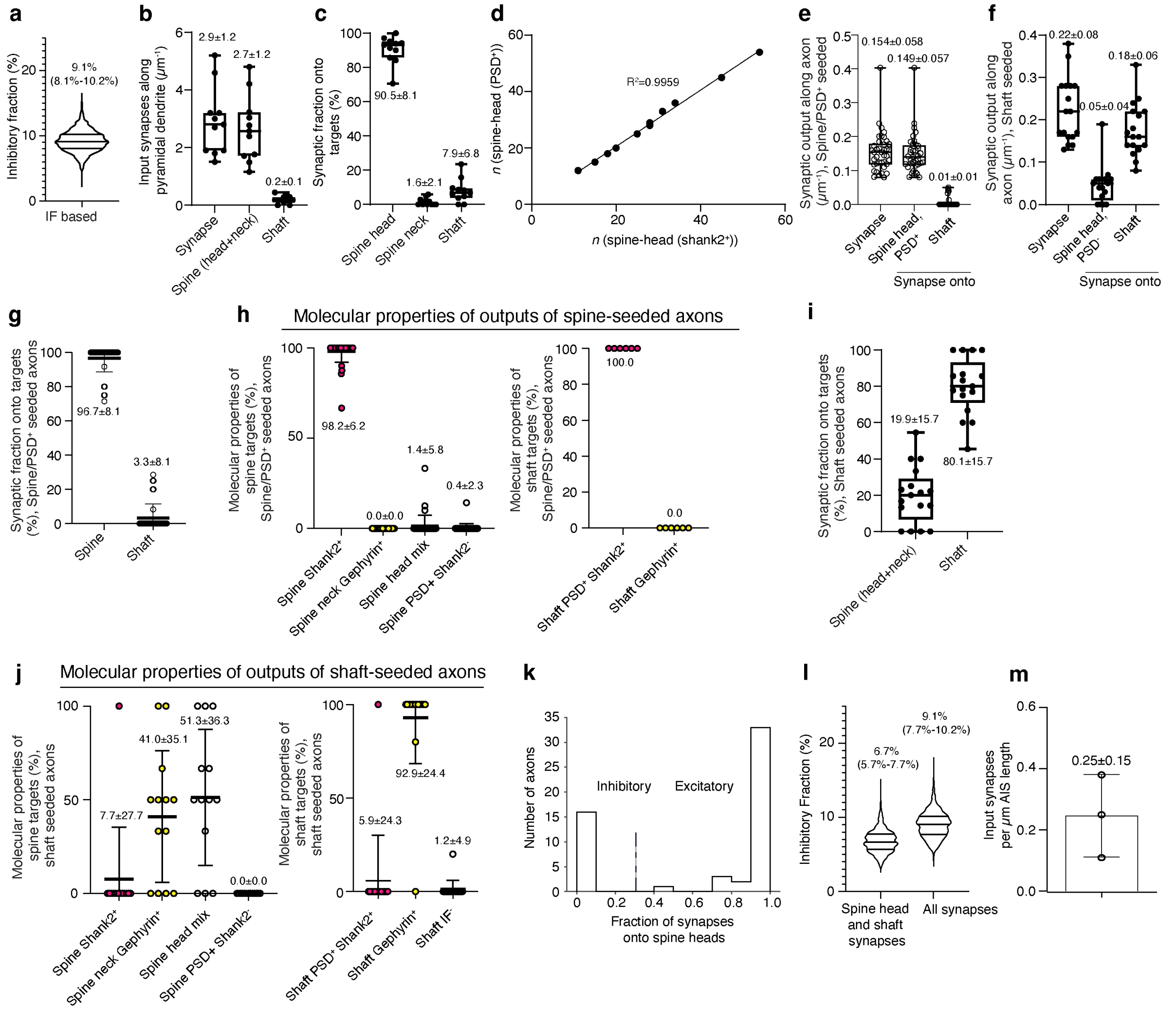

Supplementary Fig. 21| Further connectivity analysis (manual tracing) in mouse somatosensory cortex. a, Bootstrap analysis of the immunolabelling-based excitation/inhibition ratio measurement in **Fig. 4f** (351 total immunolabelling positive synapses, 11 dendrites across 2 imaging volumes in *n*=1 animal). Distribution across *n* = 1000 bootstrap samples, with median, lower and upper quartiles). **b-c,** Input properties of spiny dendrites, based on structural identification of synapses in addition to the molecular information (Shank2 and Gephyrin immunolabeling), from the same 11 dendrites across 2 imaging volumes (*n*=1 animal, same imaging volumes as in **Fig. 4b-k**). Numerical values represent mean±s.d. for all panels unless otherwise noted. Box plots: median; lower and upper quartiles; whiskers: min., max. values for all panels. Data points represent individual neurites throughout. **b,** Density of synaptic inputs onto spiny dendrites per unit length, including the total number of synapses (357), those onto spines (333), and onto shafts (24). **c,** Fraction of synaptic inputs according to target location (spine head, spine neck, shaft). **d,** Number of dendritic spine heads that feature a PSD (PSD^+) vs. number of spine heads that are positive for Shank2 ($Shank2^+$). Linear regression yields R^2 of 0.996. The 6 spine heads bearing an additional inhibitory synapse were not considered in the analysis. **e,f,** Output target locations of spine- and shaft- seeded axons (total 56 axons with ≥3 outputs (PSD^+)), including both spine-head seeded (39) and shaft-seeded (17). For spine seeding, only axons were taken that were

seeded by a spine with a single synapse (PSD positive, regardless of presence of immunolabelling signal). Shaft seeding also included one seed location where a PSD was discerned at the synapse. Same imaging volumes as in **Fig. 4b-k** (2 imaging volumes from *n*=1 animal). **e,** Density of synaptic outputs per unit length of spine-seeded axons, according to target location (total, spine head, shaft). **f,** Density of synaptic outputs per unit length of shaft-seeded axons, according to target location (total, spine head, shaft). **g,** Relative proportion of synaptic output target locations for spine-seeded axons. Mean±s.d.. **h,** Molecular properties of the outputs of spine-seeded axons. *Left*: Outputs onto spines. 98% of spine head synapses were Shank2 positive. 1.4% of Shank2 positive spine heads had an additional synapse that was Gephyrin positive. In 0.4% no immunolabelling was present but a synapse could be detected due to the presence of a PSD in the structural channel. *Right*: Output onto shafts. 100% of the shaft outputs of these particular spine-seeded axons were positive for Shank2. Shank2$^+$ synapses also had a PSD. Analyzed for the subset of axons (6) that had outputs onto shafts. Mean±s.d.. **i,** Relative proportion of synaptic output target location for shaft-seeded axons. **j,** Molecular properties of the outputs of shaft-seeded axons. *Left*: Outputs onto spines (analyzed for the 13 axons that featured outputs onto spines). 41% of these synapses were onto spine necks, which were Gephyrin positive. 51% of the outputs of these shaft-seeded axons targeted spine heads with a Gephyrin-positive connection while the same spine head was also contacted by an excitatory axon (Shank2 positive connection). We also traced one axon that was seeded at a shaft location but featured a PSD, corresponding to an excitatory axon (outlier with inverse characteristics, involved in Shank2$^+$ synapses). *Right*: Output onto shafts. These were mostly Gephyrin-positive (93%). In 1% of cases, we identified a synaptic connection based on the presence of a pre-synaptic bouton with dense projection arrangement (**Fig. 1c**), which was negative for immunolabelling (IF$^-$). Mean±s.d.. **k,l** Distribution of axons (total 55 axons selected with ≥3 outputs), including both spine-head seeded (38) and shaft-seeded (17), according to the target location of their synaptic outputs (spine head or shaft, only spine heads with single innervation (Shank2$^+$ and/or PSD$^+$ vs. Gephyrin$^+$) considered)). Synapses onto spine necks were not considered. The vast majority of axons either had a clear preference for spine heads or shafts, differentiating them into excitatory and inhibitory axons, respectively. The vertical line in the histogram (panel k) indicates the threshold for classifying them into excitatory or inhibitory according to output target location preference. Bootstrap analysis (1000 bootstrap samples, panel l) of inhibitory fraction of synaptic inputs onto the 11 dendrites analyzed above, using the excitatory/inhibitory classification of synaptic connections from axons according to panel k, yielding 6.7% inhibitory synapses. Additionally taking synapses from excitatory and inhibitory axons onto spine necks and onto spine heads that received both an excitatory and an inhibitory synapse into account, overall inhibitory fraction increased to 9.1%. Bootstrap analysis across *n* = 1000 bootstrap samples. Median with lower and upper quartiles. **m,** Density of input synapses along axonal initial segments for the 3 cells analysed in **Fig. 4k**, mean±s.d..

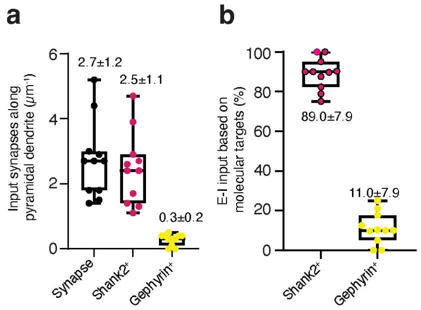

Supplementary Fig. 22| Connectivity analysis based on deep-learning prediction. a, Analysis of synaptic input density (mean±s.d.) on the same manually traced dendrites as in **Fig. 4b** (11 dendrites, across 2 imaging volumes in somatosensory cortex from *n*=1 animal), replacing immunolabeling for Shank2 with deep-learning based prediction of Shank2. Gephyrin was detected via immunolabeling as in **Fig. 4b**. Excitatory post-synapses were counted as positive if both a dense feature in the LICONN structural channel and predicted Shank2 signal were present at a synaptic location. A small number of inputs were missed, as they were outside the bounding box for the deep-learning prediction. Datapoints represent individual dendrites. Box plots: median; lower/upper quartiles; whiskers: min./max.. 4 spine heads with Shank2 prediction and an additional false positive Bassoon prediction were excluded. **b,** Excitation-inhibition balance based on predicted Shank2 signal and immunolabeling for Gephyrin, in analogy to the analysis in **Fig. 4f** (mean±s.d., 11 dendrites across 2 imaging volumes from *n*=1 animal, same imaging volumes as in Fig. **4b-k**). In both cases, connectivity results using Shank2 predicted by the deep-learning model were similar as results based on immunolabeling for Shank2. Datapoints represent individual dendrites. Box plots: median; lower/upper quartiles; whiskers: min./max..

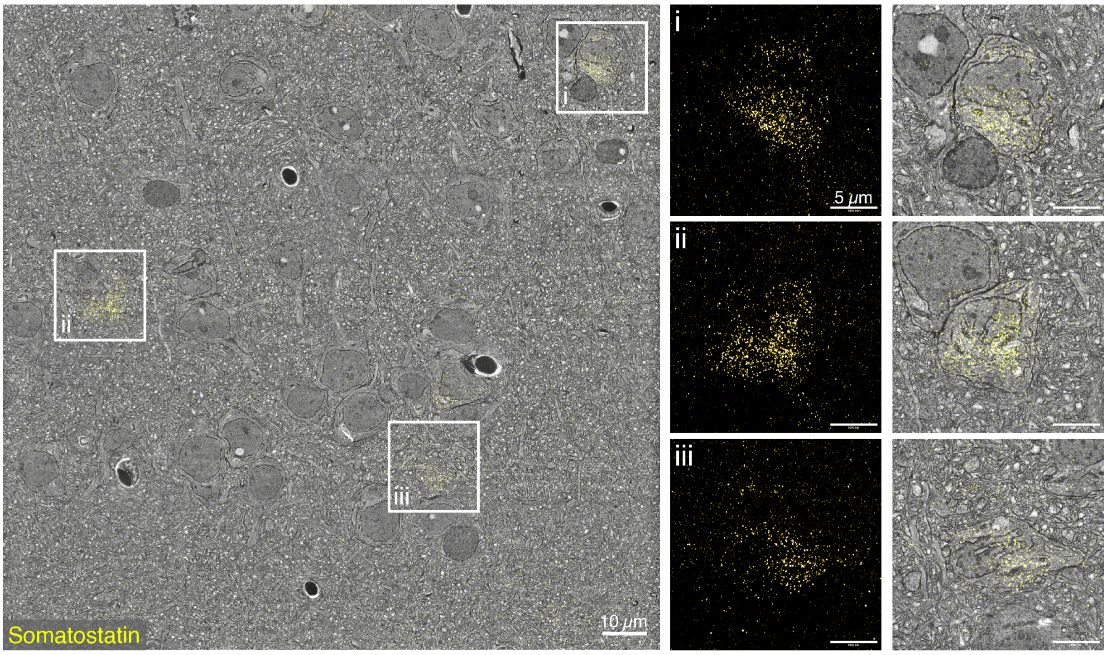

Supplementary Fig. 23| Molecular subtype labelling of neurons: somatostatin. Overview confocal image in somatosensory cortex with immunolabeling for somatostatin (yellow) overlaid with the structural channel (grey scale, single plane from a volume (same as in **Fig. 5a**) imaged in overview mode after full LICONN expansion), and enlarged views of the boxed regions. Somatostatin is expressed in a subset of GABAergic interneurons. The magnified views show individual somatostatin expressing cells as overlay and with the immunolabelling channel displayed separately. For the structural channel, a single imaging plane from a volume fused from 16 subvolumes (arranged on a 4x4) grid is displayed. The axial position of the overview imaging plane in the left panel was chosen to include the cell body displayed in magnified view (i). The other two cell bodies (magnified views (ii) and (iii)) are located at different depths (*z*-positions). For the structural channel in magnified views (ii) and (iii), imaging planes containing the respective cell bodies are shown. For visualization purposes, the immunolabeling channel is displayed as a maximum intensity projection over an axial range that spans the cell bodies located at different imaging depths (21 µm along the *z*-direction, native tissue scale). Imaging was performed after full LICONN expansion with a 20x water immersion objective lens (NA 0.95) at a voxel size of 305x305x800 nm^3. Scale bars magnified views: 5 µm. Immunolabelling was technically replicated n=2 times.

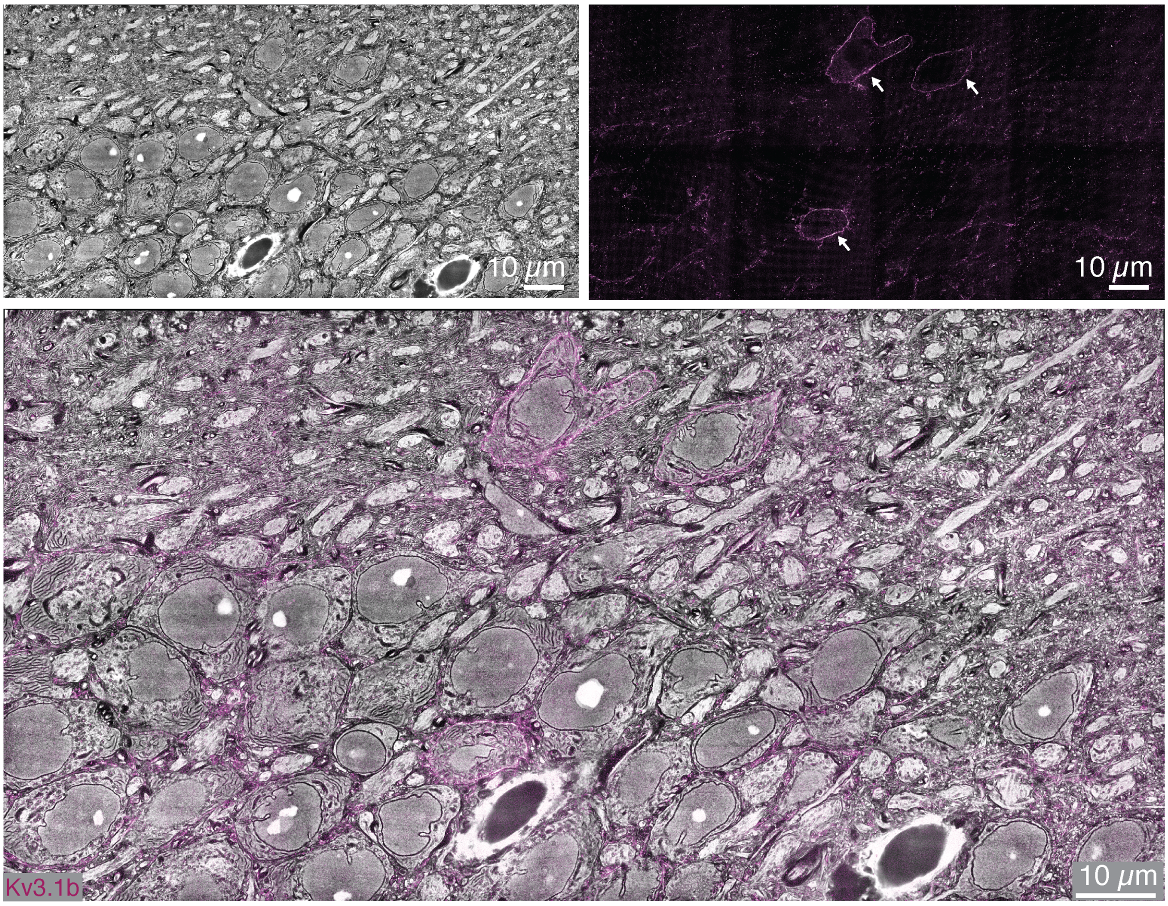

Supplementary Fig. 24| Molecular subtype labeling of neurons: Kv3.1b. Single imaging planes from a volume imaged in overview mode after full LICONN expansion in the hippocampal CA3 region, with immunolabeling for the voltage-gated potassium channel Kv3.1b (magenta). The structural (grey scale) and molecular channels are shown separately and as overlay. Kv3.1b is expressed in a subset of interneurons. Arrows point to somata of Kv3.1b expressing neurons. The volume was fused from 8 subvolumes arranged on a 4x2 grid, imaged after ~16x expansion with a 20x water immersion objective lens (NA 0.95) at a voxel size of 305x305x800 nm^3. Kv3.1b immunolabelling was technically replicated *n*=2 times.

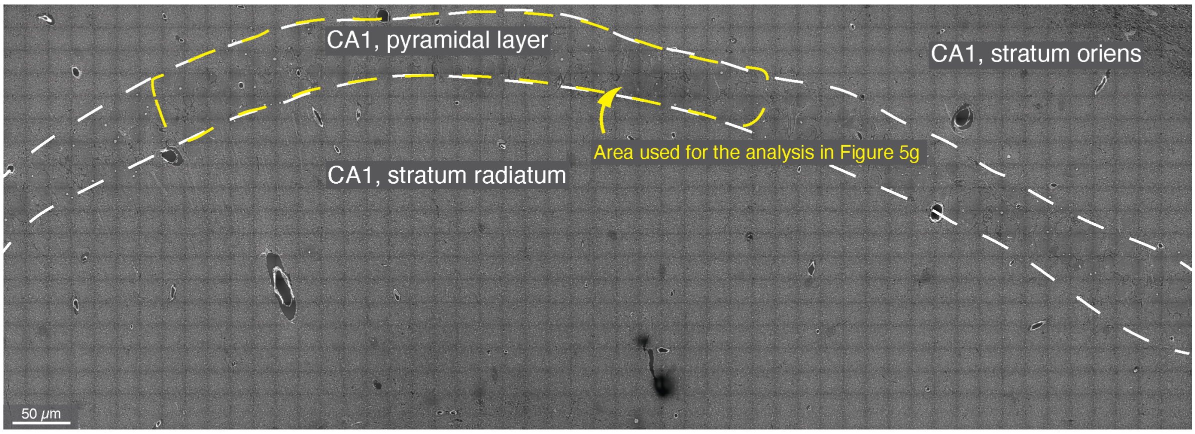

Supplementary Fig. 25| LICONN analysis in *Hnrnpu*$^{+/-}$ haploinsufficent mice. Overview in the hippocampal CA1 region, tiled from high-resolution spinning disc confocal images. The region used for analysis of cilia length in **Fig. 5** is indicated by the yellow dashed line. High-resolution overview imaging of the region analyzed in **Fig. 5g** was performed in *n*=1 specimen.
