# Supplementary material for: Light-microscopy-based connectomic reconstruction of mammalian brain tissue
Source: Nature. 2025 May 7;642(8067):398–410. doi: 10.1038/s41586-025-08985-1 (PMC12158774; doi:10.1038/s41586-025-08985-1)
Supplement: Supplementary file 2 — Reporting Summary [file 41586_2025_8985_MOESM2_ESM.pdf]

nature portfolio

Corresponding author(s): Johann G. Danzl

Last updated by author(s): Feb 19, 2025

Reporting Summary

Nature Portfolio wishes to improve the reproducibility of the work that we publish. This form provides structure for consistency and transparency in reporting. For further information on Nature Portfolio policies, see our Editorial Policies and the Editorial Policy Checklist.

Statistics

For all statistical analyses, confirm that the following items are present in the figure legend, table legend, main text, or Methods section.

n/a	Confirmed	
☐	☒	The exact sample size (*n*) for each experimental group/condition, given as a discrete number and unit of measurement
☐	☒	A statement on whether measurements were taken from distinct samples or whether the same sample was measured repeatedly
☐	☒	The statistical test(s) used AND whether they are one- or two-sided<br>*Only common tests should be described solely by name; describe more complex techniques in the Methods section.*
☒	☐	A description of all covariates tested
☒	☐	A description of any assumptions or corrections, such as tests of normality and adjustment for multiple comparisons
☐	☒	A full description of the statistical parameters including central tendency (e.g. means) or other basic estimates (e.g. regression coefficient) AND variation (e.g. standard deviation) or associated estimates of uncertainty (e.g. confidence intervals)
☐	☒	For null hypothesis testing, the test statistic (e.g. *F*, *t*, *r*) with confidence intervals, effect sizes, degrees of freedom and *P* value noted<br>*Give P values as exact values whenever suitable.*
☒	☐	For Bayesian analysis, information on the choice of priors and Markov chain Monte Carlo settings
☒	☐	For hierarchical and complex designs, identification of the appropriate level for tests and full reporting of outcomes
☐	☒	Estimates of effect sizes (e.g. Cohen's *d*, Pearson's *r*), indicating how they were calculated

Our web collection on statistics for biologists contains articles on many of the points above.

Software and code

Policy information about availability of computer code

Data collection

Imaging was performed on an Andor Dragonfly microscope based on a Nikon Ti2E inverted stand with motorized stage and an Andor Zyla 4.2 Megapixel sCMOS camera. Data was acquired using Andor Fusion software version 2.2.

Data analysis

Analysis was mostly performed with open source tools, as detailed below. Custom code is available at https://github.com/danzllab/LICONN.
Data conversion for downstream analysis was performed with custom python scripts implemented in Python v3.8 or higher, including the Imaris-ims-file-reader, zarr, webKnossos and tifffile packages. Package versions are specified in the respective configuration (.yml) files.
Imaging data were processed using Fiji v.1.54f, including the CLAHE, BigWarp and BigData viewer plugins. CLAHE was applied either in FIJI or using custom python scripts avaialble at https://github.com/danzllab/LICONN.
Overlay of immunolabellings with LICONN data was performed in Gimp version 2.10.34.
3D-renderings of cilia were done with Imaris version 9.3.
Code for analysis of distortions was adapted from (https://github.com/Yujie-S/Click-ExM_data_process_and_example). Related custom scripts are available at https://github.com/danzllab/LICONN.
Distortion vector fields were calculated using MATLAB (version R2022b, MathWorks).
We extended SOFIMA (https://github.com/google-research/sofima, git hash 64d5c7c) to support seamless stitching of 3D tiles laid out on a 2D grid and across overlapping multi-tile slabs in z-direction. Data handling was done with the tensorstore library (google.github.io/tensorstore, version 0.1.33).
For manual segmentation, we used VAST version 1.4.0 (downloaded from https://lichtman.rc.fas.harvard.edu/vast/).
For visualization in Fig. 1g, segmentation was partially proofread using VAST v1.4.1.
We used webKnossos v.22.05.1 for manual skeletonization.
We used flood-filling networks (FFNs) to automatically segment the datasets. FFNs are available at https://github.com/google/ffn. For training, the AdamW optimizer was used. Automated skeletonization of the base segmentation was done using the TEASAR algorithm (https://

github.com/seung-lab/kimimaro, version b390a9abdde60ec81472f1901e7980a962ecd847).
A Jupyter notebook for performing FFN inference on LICONN data is available at: https://github.com/google/ffn/blob/master/notebooks/jax_ffn_inference_liconn.ipynb, git hash: 12d680e.
For manual proofreading, datasets were visualized with Neuroglancer (https://zenodo.org/records/5573294, https://github.com/google/neuroglancer, c466b24).
Automated synapse detection and connectivity analysis were performed with Python scripts available at https://github.com/danzllab/LICONN.
Ground truth annotations for validating synapse detection were generated with webKnossos v.22.05.1.
We implemented deep-learning pipelines for synapse prediction with Pytorch v.1.12.1 (https://pytorch.org) and used the Gunpowder framework v 1.2.2 (https://github.com/funkelab/gunpowder) to implement our data loading, augmentation, training, and prediction pipeline.
For training, Adam's optimizer as implemented in Pytorch (torch.optim.Adam) was used.
On ISTA's HPC cluster, either single nodes or SLURM (slurm-wlm 22.05.8) were used for task allocation.
Neurite and synaptic segmentations, skeletons, and imaging volumes were visualized in 3D and video files generated with Blender versions 2.92, 3.2, or 3.51 (https:// www.blender.org/).
GraphPad Prism version 10.1.2 was used for statistical analysis and for plotting. Bootstrap analysis in Extended Data Fig. 24 was performed based on code available at https://gitlab.mpcdf.mpg.de/connectomics/human_primate. The violin plot in Fig. 2g was generated with seaborn.violinplot (https://seaborn.pydata.org/generated/seaborn.violinplot.html).
Figures were created in Adobe Illustrator version 27.5 and 27.7. The schematic in Fig. 5d and brain cross section schematics in Supplementary Fig. 4 were created in BioRender (2025, https://BioRender.com/j05y134 and https://BioRender.com/x93f652).

For manuscripts utilizing custom algorithms or software that are central to the research but not yet described in published literature, software must be made available to editors and reviewers. We strongly encourage code deposition in a community repository (e.g. GitHub). See the Nature Portfolio guidelines for submitting code & software for further information.

Data

Policy information about availability of data

All manuscripts must include a data availability statement. This statement should provide the following information, where applicable:

- Accession codes, unique identifiers, or web links for publicly available datasets
- A description of any restrictions on data availability
- For clinical datasets or third party data, please ensure that the statement adheres to our policy

Original data and/or segmentations for example datasets are available in browsable format via Neuroglancer:

Full segmentation with comprehensive proofreading of neuronal structures:
Fig. 2a-f: https://neuroglancer-demo.appspot.com/#!gs://liconn-public/ng_states/expid82.json
Fig. 2c: https://neuroglancer-demo.appspot.com/#!gs://liconn-public/ng_states/expid82_fig2_axons.json
Fig. 2d: https://neuroglancer-demo.appspot.com/#!gs://liconn-public/ng_states/expid82_fig2_dends.json

Additional datasets with lateral and/or axial fusion:
Fig. 1a-b: https://neuroglancer-demo.appspot.com/#!gs://liconn-public/ng_states/fig1a.json
Extended Data Fig. 10c-f:
https://neuroglancer-demo.appspot.com/#!gs://liconn-public/ng_states/expid146.json
Extended Data Fig. 10h:
https://neuroglancer-demo.appspot.com/#!gs://liconn-public/ng_states/multiround_fusion.json

Further source data are available at the Institute of Science and Technology Austria's data repository:
https://doi.org/10.15479/AT:ISTA:18697 (https://research-explorer.ista.ac.at/record/18697).

Research involving human participants, their data, or biological material

Policy information about studies with human participants or human data. See also policy information about sex, gender (identity/presentation), and sexual orientation and race, ethnicity and racism.

Reporting on sex and gender	n/a. No human participants or human data were used in the study.
Reporting on race, ethnicity, or other socially relevant groupings	n/a
Population characteristics	n/a
Recruitment	n/a
Ethics oversight	n/a

Note that full information on the approval of the study protocol must also be provided in the manuscript.

Field-specific reporting

Please select the one below that is the best fit for your research. If you are not sure, read the appropriate sections before making your selection.

☒ Life sciences ☐ Behavioural & social sciences ☐ Ecological, evolutionary & environmental sciences

For a reference copy of the document with all sections, see nature.com/documents/nr-reporting-summary-flat.pdf

Life sciences study design

All studies must disclose on these points even when the disclosure is negative.

Sample size

This is a proof-of-concept study focusing on the development of new technology and its applicability. Accordingly, no prior determination of sample size was performed. Once the technology was established and the data quality to our satisfaction, experiments were performed in multiple replicates to ensure and demonstrate reproducibility.
For comparison of primary cilia length in wild type vs. Hnrnpu+/- mice, we chose n=3 biological replicates in each group with 3 technical replicates each to demonstrate that a biologically meaningful number of replicates can be analysed with LICONN. No randomization between experimental groups was performed.

Data exclusions

Images typically represent views focusing on specific areas of interest from larger volumetric datasets. For high quality representation of tissue structure and traceability, optimum tissue preservation, labeling and imaging conditions are required. We discarded datasets that were of lower quality.

Replication

Stated replicates give a lower bound how many times individual experiments were performed with similar results. As this manuscript reports on a technological development, a large number of experiments with some variation of parameters have been performed, including during the development phase. For analysis, only datasets of high labeling and imaging quality were pursued.
In all images, representative data from single experiments are shown. To confirm reproducibility of the technology, we performed a series of technical replicates which were typically recorded across several biological specimens, as indicated below. For some of the procedures that we performed routinely, most notably high-resolution/fidelity tissue expansion, the stated number of replicates gives a lower bound and we did not count additional replicates beyond n=10.
Fig. 1a,b: Imaging data representative of acquisition of expansion and multi-tile imaging in n=10 replicates across n=3 animals. Fig. 1b represents subregion of dataset from Fig. 1a. Fig. 1c: 192 synapses analyzed with multiple line profiles per synapse, in n=3 technical replicates across n=2 animals. Fig. 1d: 32 distance measurements across n=2 animals. Fig. 1e, Supplementary Fig. (SF) 13,14: 37 axon stretches analysed in 12 datasets from n=3 technical replicates across n=3 animals. Fig. 1f, SF 15: 5 datasets from n=3 technical replicates across n=2 animals. Fig. 1g, SF 6: Single tile LICONN measurements were performed at a large number of technical and biological replicates (n>>20). Manual neurite annotation was performed in n=2 datasets across n=2 animals. Fig 2a-i, SF 16: Multi-tile LICONN measurements were performed in n=10 replicates. FFN based segmentation was applied to n=6 datasets. Comprehensive proofreading of all neuronal structures (Fig. 2a-g) and quantification of segmentation results using manually generated reference skeletons (Fig. 2h, SF 16,17) were performed in n=1 dataset. Fig. 3a: Immunolabeling for Bassoon and PSD-95 was performed in n=3 technical replicates across n=2 animals. Fig. 3b: Analysis of 106 synapses in n=3 technical replicates across n=2 animals. Fig. 3c: Visualization from the dataset in Fig. 3a. Bassoon/Shank2 labelling was performed in n=5 replicates across 3 animals. Fig. 3d: Synaptic immunolabellings were replicated n=2 times, with further immunolabellings for synaptic targets in various contexts. Fig. 3e: 185 synapses analysed in 3 imaging volumes from n=1 technical replicate. Fig 3f-h, SF 19: Immunolabelling based, automated synapse detection accuracy was evaluated against manually generated ground truth in n=3 technical replicates recorded across n=2 animals. Fig. 3f represents a schematic illustration of synapse detection (synapse annotations placed manually, n=1). Fig. 3i: Visualization of synapse detections was performed for n=1 dendrite. Fig. 3j,k: Immunolabelling for inhibitory synapses was performed in n=5 technical replicates. Fig. 4a-k, SF 20,21: Manual connectivity analysis was performed across 2 imaging volumes (total 154,560 µm3) in n=1 technical replicate (n=1 animal). Fig. 4b-f: Quantification of synaptic contacts of spiny dendrites was performed in n=11 dendrites. Fig. 4h,i: Output analysis and spine tracing was performed for n=19 axons. Fig. 4k: Output was analysed for n=8 AIS-seeded axons. Fig. 4 l-m, SF 19: Accuracy of deep-learning based synapse prediction was evaluated against manually generated ground truth annotations in n=3 technical replicates recorded across n=2 animals. Fig. 4n: Mapping of synaptic inputs and outputs was performed in n=1 cell and represents further analysis of the dataset in Fig. 1a. Fig. 4o: Analysis of data in Fig. 2a (n=1 dataset). Fig. 4p: Visualization of excitatory and inhibitory inputs was done for n=1 dendrite. Fig. 5a: Immunolabellings were replicated n=2 times in n=2 animals. Fig. 5b: Immunolabellings were replicated n=3 times. Fig. 5c: Imaging data representative of n=10 replicates across n=3 animals. Fig. 5e: Immunolabellings were replicated n=2 times. Fig. 5f: Analysis of 78 cells from dataset in Fig. 1a. Fig. 5g: Hnrnpu+/- haploinsufficient mice: 80 cells from n=7 technical replicates across n=3 animals; wild type: 78 cells from n=7 technical replicates across n=3 animals. Fig. 5h, Extended Data Fig. (EDF) 9: Representative of n=2 replicates in n=2 animals. Fig. 5i: Cilia diameter: 52 cross sections; Microtubule dublet distance: 54 distances measurements, analysed across n=2 technical replicates in n=1 animal (5 imaging volumes). Fig. 5j, EDF 5: Immunolabellings were reproduced in n=2 replicates. Fig. 5k: Representative of n=3 replicates. Fig. 5l: Representative of immunolabeling in n=3 replicates. Fig. 5m: Gap junction and inhibitory synapse density evaluated across n=4 imaging volumes in cortex and hippocampus from n=1 animal. EDF 2: High-resolution large-scale tiling in hippocampus was performed in n=1 replicate in n=1 animal. EDF 3: Immunolabelling for RIM1/2 and vGlut1 is representative of n=2 technical replicates and Bassoon/PSD95 immunolabelling of n=3 replicates across n=2 animals. EDF 5: The displayed combinations of immunolabellings for MBP/Cnx-43 were performed in n=1 technical replicate, RIM1/2 and vGlut1 in n=2 technical replicates, GFAP/Cnx-43 in n=2 technical replicates, Vimentin/Pmp70 in n=1 replicate. EDF 6: LICONN imaging in different brain regions was performed in n=1 animal. EDF 7: Overview imaging in CA3 stratum lucidum was performed in n=2 technical replicates, single tile measurements were performed in multiple technical replicates. EDF 8: Imaging of the various layers was performed in n=1 animal. EDF 9: Data representative of replicates in n=2 animals. EDF 10 a: Imaging data representative of LICONN in 300 µm thick slices in n=3 animals. EDF 10 c-f: Post-expansion hydrogel sectioning was performed in n=3 technical replicates. EDF10 h: Block-face imaging and sectioning over 12 rounds was performed in n=1 specimen. SF 1: Data points correspond to individual technical replicates. SF1a: n=1 replicate for 0.25%, 0.15%, 0.005%, n=2 replicates otherwise. SF1b: Different hydrogel recipes were tested in the following technical replicates: #1-#4,#15: n=2; #5,#9,#11,#13: n=3; #6-#8,#10,#12,#14: n=4. SF1c: Experiments were performed in the follow technical replicates: 0.05% APS/TEMED: n=3 replicates. 0.15% APS/TEMED: #7,#8,#10,#11,#14: n=3 replicates; #13: n=2 replicates. SF1d: n=2 technical replicates. SF1e: Technical replicates: 1st hydrogel: #11,#7,#14,#8,#10: n=3; #13: n=2. Final hydrogel: n=4. SF1f: A single hydrogel was subdivided after the first gelation step. Expansion factors for the second expandable hydrogel were determined in the following technical replicates: #11: n=3; #13,#7,#14,#1: n=4. SF1g: n=4 technical replicates. SF 2: Data representative of

n=3 technical replicates. SF 3: Each condition was technically replicated n=2 times. Hydrogels #14 and #15 were replicated n=5 times. SF 4: a-c: Individual data points represent individual animals. Number of animals analyzed: SF4a-c: 20% AA: n=3; 0% AA, 5% AA, 10% AA, 12.5% AA: n=4; 15% AA: n=6. SF4f: 0% AA: n=15 cells across n=1 technical replicate; 5% AA: n=32 cells across n=3 technical replicates; 10% AA: n=98 cells across n=3 technical replicates; 12.5% AA: n=49 cells across n=2 technical replicates; 15% AA: n=74 cells across n=3 technical replicates; 20% AA: n=20 cells across n=2 technical replicates; 25% AA: n=20 cells across n=2 technical replicates; 30% AA: n=17 cells across n=2 technical replicates. SF 5: Data representative of n=3 technical replicates. SF6: Manual segmentation in datasets with NAS anchoring was performed in n=1 replicate. SF 7: Data representative of n=3 technical replicates. SF 8: Data representative of n=2 technical replicates. SF 9: Data representative of n=3 technical replicates for each condition. SF 10a: Measurement error represents mean+/-s.d. at given measurement length from sampling 2*105 pairs of points in pre- and post-expansion images (see Methods) from the specific single measurement (n=1 imaging volume) shown in the figure panel. Data representative of n=4 technical replicates from n=3 animals. SF 10b,c: Root mean square (RMS) measurement error over different measurement lengths for the first and second expansion steps and for the overall LICONN procedure were evaluated as mean+/-s.d. across n=4 technical replicates (using a total of 14 imaging volumes), recorded in cortex across n=3 animals. SF 10d: 21 imaging volumes analysed across n=6 technical replicates from n=3 animals. SF 11: Overview image in region corresponding to dataset in Fig. 1a (n=1 dataset). SF 12a,b: Data representative of application of CLAHE in n=10 technical replicates. Illustration of visual appearance of CLAHE processed data at different slopes (SF 12b) was performed in n=1 dataset. SF 18: Data representative of n=10 technical replicates. Visual illustration of resolution improvement by iterative expansion as shown in the figure was performed in n=1 specimen. SF 20, SF21: Manual tracings were performed across 2 imaging volumes from n=1 specimen (n=1 technical replicate) imaged in the high-resolution overview scan. SF22: Tracing with deep-learning prediction of synapses was performed in n=1 technical replicate. SF 23, 24: Immunolabellings were replicated n=2 times. SF 25: High-resolution overview imaging of the region analyzed in Fig. 5g was performed in n=1 specimen.

Randomization

We do not compare subjects according to experimental groups. Accordingly, no randomization was performed.

Blinding

Measurements of cellular parameters (e.g. distance measurements within synapses, periodicity, connectivity) were performed to demonstrate that quantitative information that is consistent with previous measurements can be extracted from LICONN data. Researchers were aware of the source of the data and the purpose of the study. Quantification of segmentation accuracy and synapse detection fidelity were performed to evaluate how well computational algorithms could reproduce human ground truth annotations. In these cases, blinding was not possible or appropriate. For determining accuracy of human tracings relative to independent ground-truth information from sparse eGFP expression, blinding to that independent ground-truth information was performed. Human tracers were blinded to the eGFP channel for the analysis in Fig. 1e,f and Supplementary Fig. 13, 14. Blinding was implemented via the permissions settings in webKnossos.

Reporting for specific materials, systems and methods

We require information from authors about some types of materials, experimental systems and methods used in many studies. Here, indicate whether each material, system or method listed is relevant to your study. If you are not sure if a list item applies to your research, read the appropriate section before selecting a response.

Materials & experimental systems

n/a	Involved in the study	
☐	☒	Antibodies
☒	☐	Eukaryotic cell lines
☒	☐	Palaeontology and archaeology
☐	☒	Animals and other organisms
☒	☐	Clinical data
☒	☐	Dual use research of concern
☒	☐	Plants

Methods

n/a	Involved in the study	
☒	☐	ChIP-seq
☒	☐	Flow cytometry
☒	☐	MRI-based neuroimaging

Antibodies

Antibodies used

Primary Antibodies:
Target, host species, Abbr., Vendor, Identifier, Clonality, Working dilution
Anti-Bassoon antibody, Mouse, Anti-Bsn, Synaptic Systems, 141 011, Monoclonal, 1:300
Anti-Bassoon antibody, Rabbit, Anti-Bsn, Synaptic Systems, 141 003, Polyclonal, 1:300
Anti-RIM-1/2 antibody, Guinea pig, Anti-RIM-1/2, Synaptic Systems, 140 205, Polyclonal, 1:300
Anti-Munc13-1 antibody, Anti-Munc13-1, Synaptic Systems, 126 103, Polyclonal, 1:300
Anti-vesicular glutamate transporter 1 antibody, Rabbit, Anti-vGlut1, Synaptic Systems, 135 302, Polyclonal, 1:300
Anti-vesicular gamma-aminobutyric acid transporter antibody, Rabbit, Anti-vGAT, Synaptic Systems, 131 003, Polyclonal, 1:200
Anti-Ca2+ P/Q antibody, Guinea pig, Anti- Ca2+ P/Q, Synaptic Systems, 152 205, Polyclonal, 1:300
Anti-NMDA type glutamate receptor antibody, Mouse, Anti-GluN1, Synaptic Systems, 114 011, Monoclonal, 1:300
Anti-Postsynaptic density-95 antibody, Mouse, Anti-PSD95, Thermo Fisher, MA1-046, Monoclonal, 1:400
Anti-Shank2 antibody, Guinea pig, Anti-Shank2, Synaptic Systems, 162 204, Polyclonal, 1:300
Anti-Shank3 antibody, Guinea pig, Anti-Shank3, Synaptic Systems, 162 304, Polyclonal, 1:300
Anti-Shank1/2/3 antibody, Mouse, Anti-Shank1/2/3, Santa Cruz Biotechnology, Sc-393963 AC, Monoclonal, 1:100
Anti-Gephyrin antibody, Mouse, Anti-Gephyrin, Synaptic Systems, 147 111, Monoclonal, 1:300
Anti-Gephyrin antibody, Mouse, Anti-Gephyrin, Santa Cruz Biotechnology, sc-25311, Monoclonal, 1:200
Anti-glial fibrillary acidic protein antibody, Mouse, Anti-GFAP, Synaptic Systems, 173 011, Monoclonal, 1:300
Anti-glial fibrillary acidic protein antibody, Rabbit, Anti-GFAP, ThermoFisher, PA1-10019 ,Polyclonal, 1:400
Anti-Vimentin antibody, Guinea pig, Anti-Vimentin, Synaptic Systems, 172 004, Polyclonal, 1:300
Anti-Somatostatin antibody, Mouse, Anti-SST, Sanza Cruz Biotechnology, sc-74556, Monoclonal, 1:100
Anti-Somatostatin antibody, Rat, Anti-SST, Sanza Cruz Biotechnology, sc-47706, Monoclonal, 1:100

Anti-Somatostatin antibody, Chicken, Anti-SST, Synaptic Systems, 366 006, Polyclonal, 1:300
Anti-Somatostatin antibody, Rat, Anti-SST, Sanza Cruz Biotechnology, sc-47706, Monoclonal, 1:100
Anti-Somatostatin antibody, Rat, Anti-SST, Sigma-Aldrich/Merck, MAB354, Monoclonal, 1:100
Anti-Somatostatin antibody, Mouse, Anti-SST, Sanza Cruz Biotechnology, sc-55565, Monoclonal, 1:100
Anti-KV3.1b, Rabbit, Anti-KV3.1b, Synaptic Systems, 242003, Polyclonal, 1:300
Anti-Acetylated tubulin antibody, Mouse, Anti-a-Tubulin, Sigma-Aldrich, T7451, Monoclonal, 1:200
Anti-Adenylate cyclase-3 antibody, Rabbit, Anti-AC3, NOVUS Biologicals, NBP1-92683, Polyclonal ,1:200
Anti-Ankyrin G antibody, Guinea pig, Anti-Ankyrin G, Synaptic Systems, 386 005, Polyclonal, 1:300
Anti-Green fluorescent protein antibody, Rabbit, Anti-GFP, Invitrogen, A11120, Monoclonal, 1:300
Anti-Myelin basic protein antibody, Mouse, Anti-MBP, BioLegend, 808403, Monoclonal, 1:200
Anti-Connexin43 antibody, Rabbit, Anti-CnX-43, Sigma-Aldrich, C6219, Polyclonal, 1:200
Anti-Pmp70 antibody, Rabbit, Anti-Pmp70, Abcam, ab85550, Polyclonal, 1:400

Secondary Antibodies:
Target, Species, fluorophore, Vendor, Identifier, Working dilution
Mouse IgG (H+L), Goat, Alexa Fluor 488, Sigma, A11001, 1:400
Mouse IgG (H+L), Goat, Alexa Fluor 546, Sigma, A11030, 1:400
Mouse IgG (H+L), Goat, STAR RED, Abberior, STRED-1001, 1:200
Rabbit IgG (H+L), Goat, Alexa Fluor Plus 488, Sigma, A32731, 1:400
Rabbit IgG (H+L), Goat, Alexa Fluor 546, Sigma, A11035, 1:400
Rabbit IgG, Goat, STAR RED, Abberior, STRED-1002, 1:200
Guinea pig IgG, Goat, STAR RED, Abberior, STRED-1006, 1:200
Rat IgG, Goat, STAR 635 P, Abberior, ST635p-1007, 1:100
Rat IgG, Goat, STAR 580, Abberior ST580-1007, 1:100
Chicken IgG, Goat, Alexa Fluor 594, Thermo Fisher/Invitrogen, A-11042, 1:300

Additional antibodies tested:
Anti-alpha/beta SNAP-25, Rabbit, Synaptic Systems, 111 002, Polyclonal, 1:300
Anti-bFos, Mouse, Santa Cruz Biotech., sc-398595, Monoclonal, 1:100
Anti-cFos, Rabbit, Synaptic Systems, 226 008, Monoclonal, 1:50
Anti-cFos, Rabbit, Cell Signaling Technology, 2250, Monoclonal, 1:50
Anti-Connexin-35/36, Mouse, Merck, MAB3045, Monoclonal, 1:100
Anti-Connexin-36, Rabbit, Thermo Fisher, 51-6200, Polyclonal, 1:200
Anti-Connexin-36, Mouse, Santa Cruz Biotech., sc-398063, Monoclonal, 1:100
Anti-Connexin-45, Rabbit, Thermo Fisher, 40-7000, Polyclonal, 1:200
Anti-Dynamin-1/2/3, Rabbit, Synaptic Systems, 115 002 , Polyclonal, 1:300
Anti-glial fibrillary acidic protein, Mouse, Santa Cruz Biotech., sc-33673, Monoclonal, 1:200
Anti-Glutamic acid decarboxylase 1/67, Rabbit, Synaptic Systems, 198 208, Monoclonal, 1:300
Anti-Glutamic acid decarboxylase 65/67, Mouse, Santa Cruz Biotech., sc-365180 , Monoclonal, 1:100
Anti-Green fluorescent protein, Mouse, Thermo Fisher, A11120, Monoclonal, 1:300
Anti-IBA1, Rabbit, Synaptic Systems, 234 013, Polyclonal, 1:300
Anti-IBA1, Chicken, Synaptic Systems, 234 009 , Monoclonal, 1:300
Anti-Myelin basic protein, Mouse, Santa Cruz Biotech., sc-271524, Monoclonal, 1:100
Anti-Nucleoporin-98, Rabbit, Cell Signaling Technology, 2598, Monoclonal, 1:200
Anti-Parvalbumin, Guinea pig, Synaptic Systems, 195 004, Polyclonal, 1:200
Anti-Parvalbumin, Guinea pig, Synaptic Systems, 195 308, Monoclonal, 1:300
Anti-Parvalbumin, Goat, Abcam, ab32895, Polyclonal, 1:300
Anti-Parvalbumin, Rat, Abcam, ab11427, Polyclonal, 1:100
Anti-Parvalbumin, Rabbit, Synaptic Systems, 195 002, Polyclonal, 1:200
Anti-Piccolo, Guinea pig, Synaptic Systems, 142 104, Polyclonal , 1:300
Anti-S100B, Guinea pig , Synaptic Systems, 287 004, Polyclonal, 1:300
Anti-Synapatophysin 1, Guinea pig, Synaptic Systems, 101 004, Polyclonal, 1:300
Anti-Synaptic vesicle 2A protein, Guinea pig, Synaptic Systems, 119 004 , Polyclonal, 1:300
Anti-Synaptophysin, Rabbit, Cell Signaling Technology, 36406, Monoclonal, 1:100
Anti-Synaptotagmin1, Guinea pig, Synaptic Systems, 105 015, Polyclonal, 1:300
Anti-vesicular GABA transporter, Synaptic Systems, 131 006, Polyclonal, 1:200

Validation

Not all antibodies are expected to work with post-expansion immunolabeling, as the expansion procedure chemically modifies and denatures proteins. Stainings with the antibodies included in the study yielded the expected staining patterns for their target structures within the super-resolved tissue context.
In addition, the following statements of validation or specificity were available from the manufacturers of the primary antibodies:

Anti-Bsn (monoclonal): Specific for Bassoon. Reacts with: rat, mouse. No further validation statement available from manufacturer.
Anti-Bsn (polyclonal): Specific for Bassoon. Reacts with: rat, mouse. No signal: chicken. ExM: This antibody has been successfully used for the epitope-preserving magnified analysis of the proteome (eMAP) expansion microscopy method (Park et al. 2021. PMID: 34767453).
Anti-RIM-1/2: RIM 2 including splice variants, cross reacts to RIM 1. Reacts with: rat, mouse. ExM: This antibody has been successfully used for the magnified analysis of the proteome (MAP) expansion microscopy method (MAP; Ku et al. 2016. Nature Biotechnology 34:973-981)
Anti-Munc13-1: K.O. validated. Reacts with human, rat, mouse, zebrafish.
Anti-vGlut1: K.O. validated. Reacts with human, rat, mouse, cow, goat, dog, sheep, ape.
Anti-vGAT: K.O. validated. Reacts with human, rat, mouse, monkey.
Anti- Ca2+ P/Q: Reacts with: rat, mouse. No further validation statement available from manufacturer.
Anti-GluN1: K.O. validated. Reacts with human, rat, mouse, zebrafish.
Anti-PSD95: K.O. validated. Reacts with human, mouse, rat, xenopus.

Anti-Shank2: K.O. validated. Reacts with mouse. Specific for Shank2.
Anti-Shank3: K.O. validated. Antigen used for immunization is present in all Shank3 isoforms described for rat and in all isoforms described for mouse except Shank3-B and Shank3-C4. Reacts with rat and mouse.
Anti-Shank1/2/3: specific for an epitope mapping between amino acids 2124-2153 at the C-terminus of Shank 1 of human origin. Reacts with mouse, rat, human. No further validation statement available from manufacturer.
Anti-Gephyrin (Synaptic Systems): K.O. validated. Detects all splice variants that contain a complete E-domain including the C6 domain. Reacts with human, rat, mouse, zebrafish.
Anti-Gephyrin (Santa Cruz Biotechnology): Gephyrin (G-6) is a mouse monoclonal antibody raised against amino acids 437-736 of Gephyrin of human origin. Reacts with mouse, rat and human. No further validation statement available from manufacturer.
Anti-GFAP (Synaptic Systems): K.O. validated. Specific for GFAP isoform 1 (alpha). Reacts with human, rat, mouse, cow.
Anti-GFAP (Thermo Fisher): This Antibody was verified by Cell treatment to ensure that the antibody binds to the antigen stated. Reacts with mouse, human, rat, bovine, horse, pig.
Anti-Vimentin: Reacts with mouse and rat. No further validation statement available from manufacturer.
Anti-SST (sc-74556): Anti-Somatostatin Antibody (H-11) is recommended for detection of Somatostatin of mouse, rat and human origin by WB, IP, IF, IHC(P) and ELISA; also reactive with additional species, including and equine, bovine, porcine and canine. No further validation statement available from manufacturer.
Anti-SST (366 006): Reacts with: rat (P60042), mouse (P60041).This antibody preferentially recognizes somatostatin-28. It only shows minor cross-reactivity to the unprocessed precursor protein and does not detect somatostatin-14. No further validation statement available from manufacturer.
Anti-SST (sc-47706): Anti-Somatostatin Antibody (YC7) is recommended for detection of Somatostatin of mouse, rat and human origin by WB, IP, IF and IHC(P). No further validation statement available from manufacturer.
Anti-SST (MAB354): Anti-SST, clone YC7 recognizes Somatostatin. Shows no cross-reactivity to enkephalins, other endorphins, substance P or CGRP. Partially cross-reacts with somatostatin fragments. No further validation statement available from manufacturer.
Anti-SST (sc-55565): Somatostatin Antibody (G-10) is recommended for detection of Somatostatin of human origin by WB, IP, IF, IHC(P) and ELISA. No further validation statement available from manufacturer.
Anti-KV3.1b: Reacts with: human (P48547), rat (P25122), mouse (P15388), cow. Knock-out validated.
Anti-a-Tubulin: The antibody has been used to detect acetylated α-tubulins from many organisms including protista, plants, invertebrates, and vertebrates. The antibody recognizes an epitope located on the α3 isoform of Chlamydomonas axonemal α-tubulin, within four residues of Lys40 when this amino acid is acetylated. Reacts with bovine, frog, invertebrates, human, hamster, mouse, protista, pig, monkey, chicken, rat, plant. No further validation statement available from manufacturer.
Anti-AC3: Reacts with human, mouse, rat. Immunogen affinity purified. No further validation statement available from manufacturer.
Anti-Ankyrin G: Specific for Ankyrin G, detects all described splice variants. Reacts with rat, mouse. No further validation statement available from manufacturer.
Anti-GFP: This Antibody was verified by relative expression to ensure that the antibody binds to the antigen stated.
Anti-MBP: Each lot of this antibody is quality control tested by formalin-fixed paraffin-embedded immunohistochemical staining. Affinity purified. Reacts with human, mouse, rat. No further validation statement available from manufacturer.
Anti-CnX-43: Anti-Connexin 43 reacts specifically with connexin 43. By immunoblotting, the antibody detects a single band or 2-3 bands at 43 kDa region. Staining of connexin 43 band(s) by immunoblotting is specifically inhibited with the connexin 43 peptide. Reacts with human, bovine, rat, mouse, hamster and chicken connexin 43.
Anti-Pmp70: Knock-out validated. Replenishment batches of the polyclonal antibody, ab85550 are tested in WB. Reacts with mouse, rat and human.

Further antibodies:
Anti-alpha/beta SNAP-25, Synaptic Systems, 111 002: Synthetic peptide corresponding to AA 192 to 206 from human SNAP25. Recognizes the Botulinum neurotoxin A cleavage product with reduced affinity. Does not detect the neurotoxin E cleavage product. Recognizes splice variants SNAP 25A and B. No further validation statement available from manufacturer.
Anti-bFos, Santa Cruz Biotech., sc-398595: No further validation statement available from manufacturer.
Anti-cFos, Synaptic Systems, 226 008: Reacts with: mouse (P01101), rat (P12841), human (P01100). No further validation statement available from manufacturer.
Anti-cFos, Cell Signaling Technology, 2250: This antibody detects endogenous levels of total c-Fos protein. The antibody does not cross-react with other Fos proteins, including FosB, FRA1 and FRA2. c-Fos (9F6) Rabbit mAb #2250 non-specifically stains fixed frozen mouse spleen and liver by immunofluorescence. No further validation statement available from manufacturer.
Anti-Connexin-35/36, Merck, MAB3045: Detect Connexin 35/36 using this Anti-Connexin 35/36 Antibody, clone 8F6.2 validated for use in IH. No further validation statement available from manufacturer.
Anti-Connexin-36, Thermo Fisher, 51-6200: No further validation statement available from manufacturer.
Anti-Connexin-36, Santa Cruz Biotech., sc-398063: connexin 36 (H-9) is recommended for detection of connexin 36 of mouse, rat and human origin by Western Blotting, immunoprecipitation, immunofluorescence and solid phase ELISA. No further validation statement available from manufacturer.
Anti-Connexin-45, Thermo Fisher, 40-7000: No further validation statement available from manufacturer.
Anti-Dynamin-1/2/3, Synaptic Systems, 115 002 : K.D. validated
Anti-glial fibrillary acidic protein, Santa Cruz Biotech., sc-33673: validated by overexpression.
Anti-Glutamic acid decarboxylase 1/67, Synaptic Systems, 198 208: No further validation statement available from manufacturer.
Anti-Glutamic acid decarboxylase 65/67, Santa Cruz Biotech., sc-365180 : No further validation statement available from manufacturer.
Anti-Green fluorescent protein, Thermo Fisher, A11120: Validated by transfection.
Anti-IBA1, Synaptic Systems, 234 013: K.O. validated.
Anti-IBA1, Synaptic Systems, 234 009: No further validation statement available from manufacturer.
Anti-Myelin basic protein, Santa Cruz Biotech., sc-271524: No further validation statement available from manufacturer.
Anti-Nucleoporin-98, Cell Signaling Technology, 2598: No further validation statement available from manufacturer.
Anti-Parvalbumin, Synaptic Systems, 195 004: No further validation statement available from manufacturer.
Anti-Parvalbumin, Synaptic Systems, 195 308: No further validation statement available from manufacturer.
Anti-Parvalbumin, Abcam, ab32895: Purified from goat serum by ammonium sulphate precipitation followed by antigen affinity chromatography using the immunizing peptide. No further validation statement available from manufacturer.
Anti-Parvalbumin, Abcam, ab11427: Affinity purification Immunogen. No further validation statement available from manufacturer.

Anti-Parvalbumin, Synaptic Systems, 195 002: Reacts with: human (P20472), rat (P02625), mouse (P32848), grashopper. No further validation statement available from manufacturer.
Anti-Piccolo, Synaptic Systems, 142 104: K.O. validated.
Anti-S100B, Synaptic Systems, 287 004: Reacts with: rat (P04631), mouse (P50114), human (P04271). No further validation statement available from manufacturer.
Anti-Synapatophysin 1, Synaptic Systems, 101 004: Product discontinued. No further validation statement available from manufacturer.
Anti-Synaptic vesicle 2A protein, Synaptic Systems, 119 004: Reacts with: human (Q7L0J3), rat (Q02563), mouse (Q9JIS5). No further validation statement available from manufacturer.
Anti-Synaptophysin, Cell Signaling Technology, 36406: No further validation statement available from manufacturer.
Anti-Synaptotagmin1, Synaptic Systems, 105 015: K.O. validated.
Anti-vesicular GABA transporter, Synaptic Systems, 131 006: Product discontinued. No further validation statement available from manufacturer.

Animals and other research organisms

Policy information about studies involving animals; ARRIVE guidelines recommended for reporting animal research, and Sex and Gender in Research

Laboratory animals	Adult mice (aged typically 2-3 months, unless otherwise noted) were used as indicated with the following genotypes: C57BL/6J wild type mice, Thy1-eGFP (STOCK Tg(Thy1-eGFP)MJrs/J mice, #007788, RRID:IMSR_JAX:007788 hemizygous), and haploinsufficient Hnrnpu+/- mice (deletion of one allele of Hnrnpu spanning axons 4 to 14, generated by crossing the HnrnpUwt/flox line (Hnrnpu<tm1.1Tman>/J, Strain:#032187, RRID:IMSR_JAX:032187) with the CMV-CreCre/Cre line (B6.C-Tg(CMV-cre)1Cgn/J, Strain #:006054, RRID:IMSR_JAX:006054).
Wild animals	No wild animals were used in the study.
Reporting on sex	For demonstrating the technology, both male and female mice were used interchangeably.
Field-collected samples	The study did not include field-collected samples.
Ethics oversight	Animal procedures were performed in accordance with national law (BGBLA 114 and Directive 522), European Directive 2010/63/EU and institutional guidelines for animal experimentation and were approved by the Austrian Federal Ministry for Education, Science and Research (authorizations BMBWF-V/Sb: 2020-0.363.126, 2021-0.550.199, 2021-0.842.237, 2022-0.121.445, and 2023-0.930.355).

Note that full information on the approval of the study protocol must also be provided in the manuscript.

Plants

Seed stocks	n/a. No plants were used in the study.
Novel plant genotypes	n/a
Authentication	n/a
